# Li^+^ intercalation chemistry on 2D transition metal dichalcogenides towards phase evolution, scalable production, and application

**DOI:** 10.1093/nsr/nwag165

**Published:** 2026-03-17

**Authors:** Qingyong Zhang, Jian Jiang, Ruixin Yan, Ruijie Yang, Xiaodong Wang, Qi Wang, Zhen Zhang, Qinghua Zhang, Qutong Yang, Qingyu Dong, Yu Tang, Ting Ying, Long Zheng, Shuaihang Hou, Yanbin Shen, Furong Chen, Ye Chen, Qi Liu, M Danny Gu, Lin Gu, Lain-Jong Li, Qian Zhang, Xiao Cheng Zeng, Kian Ping Loh, Zhiyuan Zeng

**Affiliations:** Department of Materials Science and Engineering, and State Key Laboratory of Marine Environmental Health, City University of Hong Kong, Hong Kong 999077, China; Department of Materials Science and Engineering, and State Key Laboratory of Marine Environmental Health, City University of Hong Kong, Hong Kong 999077, China; Shenzhen Research Institute, City University of Hong Kong, Shenzhen 518057, China; Department of Materials Science and Engineering, and State Key Laboratory of Marine Environmental Health, City University of Hong Kong, Hong Kong 999077, China; Department of Materials Science and Engineering, and State Key Laboratory of Marine Environmental Health, City University of Hong Kong, Hong Kong 999077, China; Department of Chemical and Petroleum Engineering, University of Calgary, Calgary T2N 1N4, Canada; School of Materials Science and Engineering, Institute of Materials Genome & Big Data, Harbin Institute of Technology, Shenzhen 518055, China; Department of Materials Science and Engineering, and State Key Laboratory of Marine Environmental Health, City University of Hong Kong, Hong Kong 999077, China; Department of Materials Science and Engineering, and State Key Laboratory of Marine Environmental Health, City University of Hong Kong, Hong Kong 999077, China; Department of Materials Science and Engineering, Southern University of Science and Technology, Shenzhen 518055, China; Institute of Physics, Chinese Academy of Sciences, Beijing 100190, China; Department of Materials Science and Engineering, and State Key Laboratory of Marine Environmental Health, City University of Hong Kong, Hong Kong 999077, China; i-Lab, CAS Center for Excellence in Nanoscience, Suzhou Institute of Nano-Tech and Nano-Bionics, Chinese Academy of Sciences, Suzhou 215123, China; Department of Physics, City University of Hong Kong, Hong Kong 999077, China; Department of Materials Science and Engineering, and State Key Laboratory of Marine Environmental Health, City University of Hong Kong, Hong Kong 999077, China; Department of Chemistry, The Chinese University of Hong Kong, Hong Kong 999077, China; Hebei Key Lab of Optic-electronic Information and Materials, The College of Physics Science and Technology, Hebei University, Baoding 071002, China; i-Lab, CAS Center for Excellence in Nanoscience, Suzhou Institute of Nano-Tech and Nano-Bionics, Chinese Academy of Sciences, Suzhou 215123, China; Department of Materials Science and Engineering, and State Key Laboratory of Marine Environmental Health, City University of Hong Kong, Hong Kong 999077, China; Department of Chemistry, The Chinese University of Hong Kong, Hong Kong 999077, China; Department of Physics, City University of Hong Kong, Hong Kong 999077, China; Eastern Institute for Advanced Study, Eastern Institute of Technology, Ningbo 315200, China; Institute of Physics, Chinese Academy of Sciences, Beijing 100190, China; Department of Mechanical Engineering, University of Hong Kong, Hong Kong 999077, China; School of Materials Science and Engineering, Institute of Materials Genome & Big Data, Harbin Institute of Technology, Shenzhen 518055, China; Department of Materials Science and Engineering, and State Key Laboratory of Marine Environmental Health, City University of Hong Kong, Hong Kong 999077, China; Department of Chemistry, National University of Singapore, Singapore 117543, Singapore; Department of Materials Science and Engineering, and State Key Laboratory of Marine Environmental Health, City University of Hong Kong, Hong Kong 999077, China; Shenzhen Research Institute, City University of Hong Kong, Shenzhen 518057, China

**Keywords:** structural phase transitions, transition metal dichalcogenides, intercalation, exfoliation, thermoelectricity

## Abstract

Li^+^ intercalation chemistry is a powerful tool to induce phase transitions in transition metal dichalcogenides (TMDs), but only the transition of 2H-to-1T/1T’ in group Ⅵ TMDs (MoS_2_ and WS_2_) is well-known and widely explored for applications in areas such as transistors, memristors, catalysis, and batteries. Here, we develop a fully documented landscape of phase evolution in group IV-Ⅵ TMDs induced by electrochemical Li^+^ intercalation through *in-situ* X-ray diffraction (XRD) and Raman techniques. We found emerging structural phase evolutions that had never been noticed before, including 1T-to-1T (transition-free) in group IV TMDs (TiS_2_ and ZrS_2_), 2H-to-3R in group Ⅴ TMDs (NbS_2_), as well as 1T-to-2H in group Ⅴ TMDs (VS_2_ and TaS_2_). Theoretical calculations uncovered the crucial role played by lithium intercalation in facilitating electron transfer from the *s* orbital of lithium to the *d* orbital of the transition metal center and clarified the reasons of the difference of phase transitions for different families of TMDs. Furthermore, we discovered that phase transitions also occur in the subsequent exfoliation process for scalable preparation of TMD atomically thin sheets, embodying 1T-to-1T (transition-free) in TiS_2_, 1T-to-amorphous in ZrS_2_, 3R-to-H in NbS_2_, and 2H-to-1T in VS_2_ and TaS_2_. Our developed Li^+^ intercalation chemistry enriches phase transition nanotechnology, which facilitates not only the understanding of the mechanism of phase transition but also its control, opening up new possibilities for phase-dependent TMD-based nanoelectronic, photonic, and thermoelectric devices. As a proof-of-concept application, we developed a thermoelectric device using our exfoliated TiS_2_ nanosheets, achieving a maximum power density of 458.6 W·m^−2^ at a 53 K temperature difference.

## Teaser

This study reveals several novel phase evolutions in group IV-VI transition metal dichalcogenides (TMDs) during electrochemical lithium intercalation and exfoliation, which advance TMD phase transition nanotechnology for diverse applications.

## INTRODUCTION

**Figure 1.**
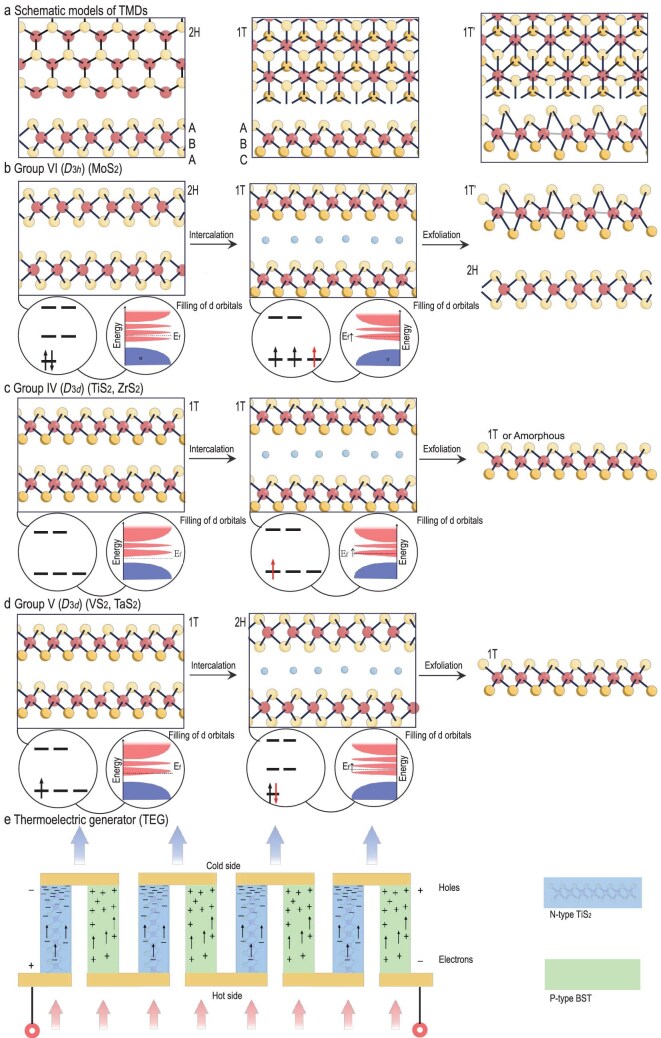
Schematic illustrations of structural phase evolutions in group IV-VI transition metal dichalcogenides (TMDs) as well as corresponding energy mechanisms and applications. (a) Schematic models of the different phases of TMDs. The 2H phase is trigonal prismatic, 1T phase is octahedral, and 1T’ phase is a distorted octahedral. The stacking order of the atomic planes for the 2H phase is Bernal stacking (ABA), and that for the 1T and 1T' phases is rhombohedral stacking (ABC). (b) Structural phase evolution in group VI TMDs induced by electrochemical lithium intercalation and exfoliation. (c) Structural phase evolution in group IV TMDs induced by electrochemical lithium intercalation and exfoliation. (d) Structural phase evolution in group V TMDs induced by electrochemical lithium intercalation and exfoliation. (e) Schematic of our developed TiS_2_ nanosheet based thermoelectric generator.

Two-dimensional (2D) transition metal dichalcogenides (TMDs) have emerged as a captivating material library with promising applications, characterized by the chemical formula MX_2_, where M represents a transition metal atom and X denotes a chalcogen atom (S, Se, or Te) [1]. This class of materials exhibits diverse crystal phases, with the most common phases being 2H, 1T, and 1T’ (see Fig. 1a, Note S1) [2].

The properties of 2D TMDs exhibit phase dependence [3]. For instance, the 2H MoS_2_ monolayer is a direct bandgap semiconductor with strong photoluminescence [4], showing great application potential in electronics and optoelectronics [5–7]. In contrast, 1T and 1T’ 2D MS_2_ exhibit metallic and semi-metallic natures, respectively, making them promising candidates for applications in energy conversion and energy storage [8–12].

Transitions between the different phases of 2D TMDs thus enable switching of material properties and applications [13]. Lithium intercalation is a powerful tool to induce such phase transitions and assist peeling of TMDs, resulting in solution-processable mono- or few-layers with changed phases [14–16]. These produced phase-engineered 2D TMDs are fascinating building blocks for supercapacitors [8,10], transistors [17], memristors [18], filter membranes [19], and so on. However, only the 2H-to-1T/1T’ phase transition is well-known and practically applied.

Here, by developing Li^+^ intercalation chemistry, beyond the well-known 2H-to-1T/1T’ phase transition in MoS_2_ (Fig. 1b), we discovered several emerging structural phase transitions during electrochemical lithium intercalations (Fig. 1c and d) by analyzing the fully documented landscapes of phase evolution in group IV-Ⅵ TMDs captured by *in-situ* X-ray diffraction (XRD) and Raman techniques (see Fig. S1 for detailed discharge curves, see Fig. S2 and Note S2 for detailed properties of the starting bulk TMDs, see Figs S3 and S4 for detailed *in-situ* testing cell). Then, we used density functional theory (DFT) calculations to reveal the underlying mechanism of these phase transitions and clarify the energy reasons of the difference of phase transitions for different families of TMDs. New phase evolutions during exfoliations for the scalable production of atomically thin sheets also appeared and were probed by us. Further, the thermoelectric application of the exfoliated nanosheets was demonstrated (Fig. 1e). Our results fill the knowledge gap of the phase transition diversity in TMDs, facilitating phase-dependent TMD applications.

## PHASE TRANSITIONS OF TMDS DURING LITHIUM INTERCALATION

### Transition-free in TiS_2_

**Figure 2.**
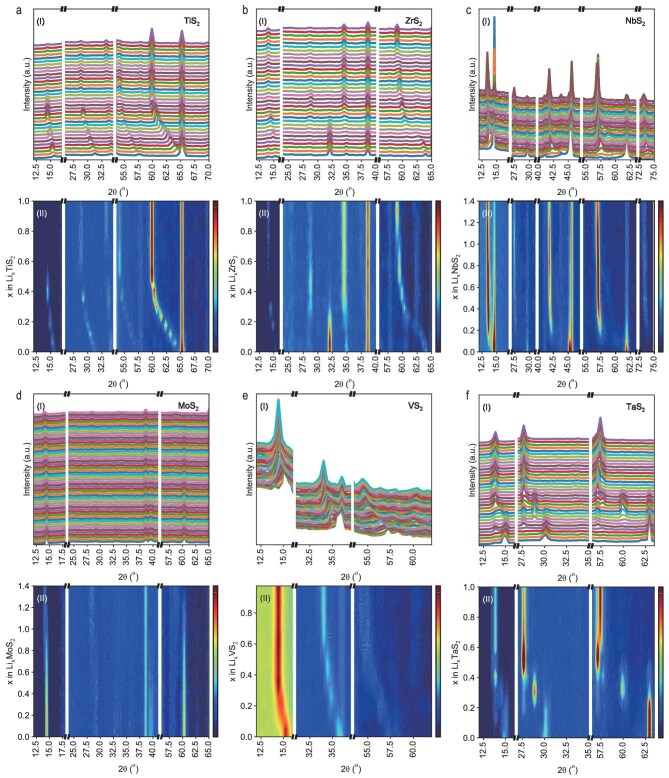
*In-situ* X-ray diffraction (XRD) patterns of TMDs. (a) Stacked *in situ* XRD patterns (I), and two-dimensional contour plots (II) of TiS_2_. The diffraction peaks shift gradually to lower angles, indicating a transition-free phase. (b) Stacked *in situ* XRD patterns (I), and two-dimensional contour plots (II) of ZrS_2_. The diffraction peaks shift gradually to a lower angle, indicating a transition-free phase. (c) Stacked *in situ* XRD patterns (I), and two-dimensional contour plots (II) of NbS_2_. A series of peaks ascribed to 3R LiNbS_2_ emerge, meaning a 2H-to-3R transition. (d) Stacked *in situ* XRD patterns (I), and two-dimensional contour plots (II) of MoS_2_. A series of peaks ascribed to 1T LiMoS_2_ emerge, meaning a 2H-to-1T transition. (e) Stacked *in situ* XRD patterns (I), and two-dimensional contour plots (II) of VS_2_. Two series of peaks emerge, meaning a 1T-to-2H transition with the appearance of an intermediate phase. (f) Stacked *in situ* XRD patterns (I), and two-dimensional contour plots (II) of TaS_2_. Several series of peaks emerge, meaning a 1T-to-2H transition with the appearance of three intermediate phases.

**Figure 3.**
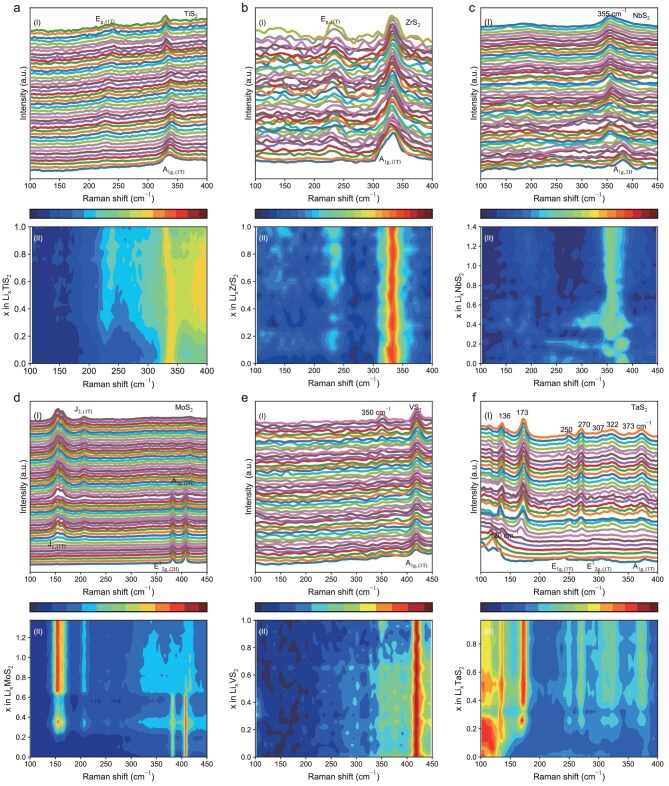
*In-situ* Raman patterns of TMDs. (a) Stacked *in-situ* Raman patterns (I), and two-dimensional contour plots (II) of TiS_2_. The vibrational modes of 1T TiS_2_ remain, indicating a transition-free phase without symmetry breaking. (b) Stacked *in-situ* Raman patterns (I), and two-dimensional contour plots (II) of ZrS_2_. The vibrational modes of 1T ZrS_2_ remain, indicating a transition-free phase without symmetry breaking. (c) Stacked *in-situ* Raman patterns (I), and two-dimensional contour plots (II) of NbS_2_. A new peak ascribed to 3R LiNbS_2_ emerges, meaning a 2H-to-3R transition. (d) Stacked *in situ* Raman patterns (I), and two-dimensional contour plots (II) of MoS_2_. The peaks ascribed to 1T LiMoS_2_ and 2H MoS_2_ coexist, meaning a 2H-to-1T transition. (e) Stacked *in-situ* Raman patterns (I), and two-dimensional contour plots (II) of VS_2_. A new peak emerges, meaning breaking of the symmetry. (f) Stacked *in-situ* Raman patterns (I), and two-dimensional contour plots (II) of TaS_2_. Several peaks emerge, meaning that phase transition occurs. For the sake of observation, we have normalized the Raman intensity.

For TiS_2_, no new peaks appear in the *in-situ* XRD spectrum (Fig. 2a and Fig. S5), signifying that no phase transition occurs during electrochemical lithium intercalation. A continuous shift to lower angles of the diffraction peaks (Fig. S6) indicates an incessant c-axis expansion (Fig. S7) caused by lithium intercalation; for instance, the c-axis expands from 0.569 nm of bulk TiS_2_ to 0.617 nm of Li_0.4_TiS_2_. A comparative analysis of the *in-situ* and *ex-situ* XRD patterns for Li_*x*_TiS_2_ is shown in Fig. S5b. The disappearance of peaks in the grey region, leaving primarily the *(00l)* series, indicates a structural relaxation during the resting period. These intermediate phases are kinetically stable under electrochemical load but thermodynamically metastable at equilibrium. *In-situ* Raman spectroscopy (Fig. 3a) of TiS_2_ reveals that the characteristic$\ {A}_{1g}$(334 cm^−1^) and ${E}_g$(237 cm^−1^) vibrational modes of the 1T phase [20] persist throughout the entire lithiation process. Both peaks shift slightly during lithium intercalation without peak splitting or the emergence of any new vibrational features, underscoring the structural stability of the 1T phase, confirming that no phase transition occurs during electrochemical lithium intercalation. This result is consistent with computational calculations, showing the lower total energy (more stable) of 1T LiTiS_2_ compared to 2H LiTiS_2_ (Fig. S8).

### Transition-free in ZrS_2_

A similar result (transition-free of the phase and the maintenance of the 1T phase during lithium intercalation) appears in ZrS_2_, evidenced by the combined *in-situ* XRD and Raman analyses. *In-situ* XRD patterns (Fig. 2b and Figs S9 and S10) show that the characteristic (001), (002), and (004) Bragg reflections undergo a continuous and monotonic shift toward lower 2θ angles throughout the lithiation process. The absence of any nascent diffraction peaks or peak splitting signifies a gradual expansion of the c-axis interlayer spacing without the formation of a secondary phase. This long-range structural integrity is further corroborated by *in-situ* Raman spectroscopy (Fig. 3b). The vibrational fingerprints of the 1T phase, namely the ${A}_{1g}$ (332 cm^−1^) and ${E}_g$ (245 cm^−1^) modes, persist as the lithium content (*x*) increases from 0.0 to 1.0. No new peaks or splitting of existing modes are observed, indicating that the ${D}_{3d}$ point group symmetry and the octahedral coordination environment remain intact. A subtle redshift of the ${A}_{1g}$ peak is detected, which is physically consistent with the lattice expansion (Fig. S11) observed in the XRD data. Collectively, these results provide robust, mutually reinforcing evidence that ZrS_2_ maintains its 1T phase during electrochemical lithium intercalation.

### 2H-to-3R transition in NbS_2_

For NbS_2_, diffraction peaks of the 3R phase (such as (003) peak at 14.56^o^, (006) peak at 27.55^o^) appear in the *in-situ* XRD patterns (Fig. 2c, Figs S12–S14) when more than 0.1 mol of lithium are intercalated in NbS_2_, implying the 2H-to-3R phase transformation during electrochemical lithium intercalation. An emerging peak in the *in-situ* Raman spectrum located at ∼355 cm^−1^ (attributed to the 3R phase ${A}_g$ mode [21] Fig. 3c) also appears, further evidencing the 2H-to-3R phase transition. This result is consistent with the energetically favorable principle (Fig. S8). Notably, the monolayer (H phase) of the 3R phase is identical to that of the 2H phase [2,3]. Therefore, lithium intercalation solely alters the stacking sequence of the H phase in NbS_2_ without affecting the plane of the sulfur atoms.

### 2H-to-1T transition in MoS_2_

The phase transition of 2H-to-1T in MoS_2_ induced by lithium intercalation is storied. Such a transition is also verified by our *in-situ* XRD (Fig. 2d and Figs S15–S17) and Raman (Fig. 3d) analyses. The (001)_1T_ diffraction peak of the 1T phase (located at 14.07^o^) appears at a lithiation degree of ∼0.3 (*x* = 0.3), signifying that the 2H-to-1T phase transition starts here. The comparison between the *in-situ* and *ex-situ* XRD patterns in Fig. S15b reveals a distinct discrepancy in structural evolution during the MoS_2_ lithiation process. Notably, certain diffraction peaks within the grey shaded region (∼32^o^ to 35^o^), disappear in the corresponding *ex-situ* patterns. This phenomenon suggests a sophisticated rearrangement of Li ions within the MoS_2_ host lattice and a subsequent structural relaxation. The *in-situ* Raman displays $J1$ (152.8 cm^−1^) and $J2$ (205.5 cm^−1^) peaks of the 1T phase, indicating the 2H-to-1T phase transition [22]. While the starting point of phase transition detected by *in-situ* Raman is *x* = 0.2 or less, which is distincted with the result of *in-situ* XRD. This is because Raman is a surface-sensitive test that can immediately detect slight changes of crystalline phases on the surface once they occur, while XRD is a bulk test that can effectively monitor the phase changes of large amounts of material and is not able to detect surface changes. XRD results better reflect the overall changes of the electrode. The (002)_2H_ diffraction peak of the 2H phase (located at 14.40^o^) completely disappears, when the amount of inserted lithium exceeds 1.4 mol per mole of MoS_2_, marking a complete phase transition. The discovery of these phase transition milestone points (starting point and completion point) provides a basis for the electrochemical control of phase transitions.

### 1T-to-2H transition in VS_2_

During electrochemical lithium intercalation, VS_2_ undergoes a complex phase transition, starting from 1T phase of bulk VS_2_, changing to an intermediate phase (IP) of Li_*x*_VS_2_ (*x* is approximately between 0.1 and 0.3) (as the new XRD peaks located at 14.6°, 34.9°, and 56.3°–56.5° appear, see Fig. 2e, Figs S18–S21), and ending with the 2H phase of Li_*x*_VS_2_ (*x* > 0.3) (as evidenced by the appearance of the 2H diffraction peaks located at 14.4°, 33.9–34.5°, and 54.3–55.8°, see Fig. 2e, Figs S18–S21). The comparison of the Li_*x*_VS_2_  *in-situ* and *ex-situ* XRD displays peaks in the *ex-situ* XRD that match their counterparts in *in-situ* XRD almost perfectly (Fig. S18). This high degree of consistency demonstrates that the phase transitions and intermediate phases observed during our *in-situ* testing are thermodynamically stable states rather than transient artifacts of the electrochemical current. In the *in-situ* Raman spectroscopy (Fig. 3e), the peak located around 415 cm^−1^ can be ascribed to the ${A}_{1g}$ vibrational mode of 1T VS_2_ [23]. After lithium intercalation, a new peak around 350 cm^−1^ appears, indicating the intercalation of lithium and symmetry breaking.

### 1T-to-2H transition in TaS_2_

Similarly, 1T-to-2H transition also occurs in TaS_2_ during lithiation with the appearance of three intermediate phases, evidenced by *in-situ* XRD patterns (Fig. 2f, Figs S22 and S23). In the overall process, TaS_2_ has gone through five phases, namely the starting 1T phase (bulk TaS_2_), the first intermediate phase (IP-I) (Li_*x*_TaS_2_, 0.1 < *x* < 0.4), the second intermediate phase (IP-II) (Li_*x*_TaS_2_, 0.25 < *x* < 0.35), the third intermediate phase (IP-III) (Li_*x*_TaS_2_, 0.4 < *x* < 0.75), and the final 2H phase (Li_*x*_TaS_2_, 0.55 < *x*) (Fig. S23). Expansions of interlayer spacing caused by intercalation are confirmed by calculations based on XRD (Fig. S24) and focused ion beam scanning transmission electron microscopy (FIB-STEM) images (Fig. S25). The diffraction peak positions of Li_*x*_TaS_2_ in *ex-situ* XRD match the *in-situ* patterns very well (Fig. S22), which demonstrates that the phase transitions and intermediate phases observed during our *in-situ* testing are thermodynamically stable states rather than transient artifacts of the electrochemical current. *In-situ* Raman spectra (Fig. 3f) doubly confirm the 1T-to-2H phase transition of TaS_2_ during lithium intercalation (see Note S3).

## GENERAL RULES AND MECHANISM FOR PHASE TRANSITIONS

We analyze the general rules and mechanism for phase transitions induced by electrochemical lithium intercalation from the following three perspectives: structure, energy, and electron distribution.

### Structure perspective

To get the general rules of phase transitions, we calculated the transition metal-sulfur (M-S) bonding length and interlayer spacing using density functional theory (DFT) calculations. By comparing the calculation results (Fig. S26, Tables S1 and S2) with the actual phase transitions detected through *in situ* characterizations, we conclude that TMDs tend to transfer to the phase with a shorter M-S bond length as well as a smaller change of M-S bond length; if the two phases have the same metal-sulfur bond length (such as LiNbS_2_), it tends to form a phase with smaller interlayer spacing.

### Energy perspective

**Figure 4.**
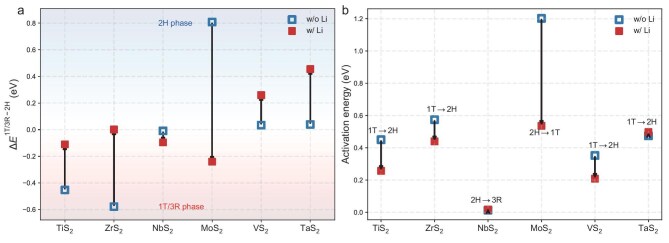
The effect of lithium intercalation on the structural phase transition of TMDs. (a) The relative energy difference of phases of MS_2_ or LiMS_2_ (M = Mo, Nb, V, Ta, Ti, Zr). $\Delta {E}^{1{\rm T/3R} - 2{\rm H}} = {E}^{1{\rm T/3R}} - {E}^{2{\rm H}}$, where ${E}^{1{\rm T/3R}}$ denotes the energy of 1T phases of MoS_2_, VS_2_, TaS_2_, TiS_2_, ZrS_2_, or 3R phase of NbS_2_, and ${E}^{2{\rm H}}$ denotes the energy of 2H phases. The legend ‘w/o Li’ means the property ($\Delta E$, activation energy) belongs to MS_2_ (without lithium intercalation), and legend ‘w/Li’ means the property ($\Delta E$, activation energy) belongs to LiMS_2_ (with lithium intercalation). (b) The activation energy barrier of the corresponding phase transition from ss-NEB calculations.

Energy differences between the 1T/3R phase and 2H phase (Δ*E*) of bulk TMDs and intercalated TMDs were also calculated. Thermodynamically, matter tends to exist at lower energies at low temperature. After lithium intercalation, TiS_2_, ZrS_2_, NbS_2_, MoS_2_, VS_2_, and TaS_2_ demonstrate preferred phases of 1T, 1T, 3R, 1T, 2H, and 2H, respectively, as the Δ*E* is negative, negative, negative, negative, positive, and positive, respectively (red box in Fig. 4a). The calculation results are consistent with *in situ* characterizations, signifying that TMDs tend to transfer to the phase with a lower energy. To investigate the kinetic influence of lithium intercalation for phase transitions, the solid-state nudged elastic band (ss-NEB) method was employed. After lithium intercalation, the phase transition energy barriers of TMDs significantly decrease (for MoS_2_ and VS_2_) or remain largely unchanged (for NbS_2_ and TaS_2_) (Fig. 4b), indicating that lithium intercalation can effectively reduce the activation energy for TMD phase transitions.

### Electron distribution perspective

To gain insights into the physical causes of phase transitions, we also performed electronic structure analyses (Figs S27–S31, Note S4). Results show that the antibonding *d* orbital will be occupied by the additional electron donated by the lithium atom after lithium intercalation, which results in changes of the M-S bond length (Table S2), thereby affecting the stability of TMDs in different phases and causing possible phase transitions. To quantitatively evaluate the extent of M-S bonding changes, we calculated the integrated crystal orbital Hamilton population (ICOHP) (Fig. S32). Taking MoS_2_ as an example, with the incorporation of lithium in MoS_2_, the bond strength in 2H-LiMoS_2_ was greatly reduced, which is weaker than 1T-LiMoS_2_, resulting in the 1T phase being a more stable phase than the 2H phase in this case and therefore leading to phase transition from 2H to 1T.

## FURTHER PHASE TRANSITIONS OF TMDS DURING EXFOLIATION

**Figure 5.**
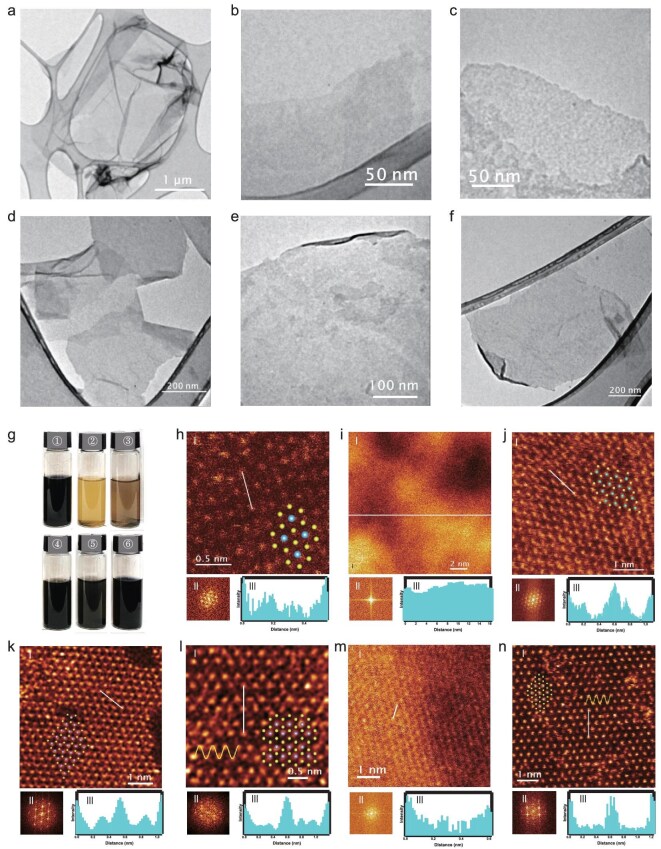
TEM and HAADF-STEM images of single/few-layer MS_2_. (a-f) TEM image of (a) TiS_2_, (b) ZrS_2_, (c) NbS_2_, (d) MoS_2_, (e) VS_2_, and (f) TaS_2_, (g) Dispersions of exfoliated TMDs (①-⑥ corresponds to TiS_2_, ZrS_2_, NbS_2_, MoS_2_, VS_2_ and TaS_2_). (h-n) HAADF-STEM images (I), corresponding FFT (II) and intensity profile (III) of the selected line (white) in (I) for (h) TiS_2_, (i) ZrS_2_, (j) NbS_2_, (k) 2H-MoS_2_, (l) 1T′-MoS_2_, (m) VS_2_ and (n) TaS_2_. Bright spots in HAADF-STEM images correspond to the positions of transition metal (M) atoms due to their higher atomic number compared to S.

The intercalated TMDs have expanded interlayer spacing and weakened interlayer adhesion, making their atomic layers easily isolated. In this work, we employed a mild sonication treatment (5–15 min) to achieve exfoliations. After sonication, mono- or few-layer TMD nanosheets were produced, as evidenced by transmission electron microscopy (TEM, Fig. 5a–f), scanning electron microscopy (SEM, Fig. S33), and the formed opaque suspension (Fig. 5g). We also illustrate the potential to produce single-/few-layer TMDs on a large scale using large area electrodes (Figs S34 and S35).

Aberration-corrected high-angle annular dark-field scanning transmission electron microscopy (HAADF-STEM) images demonstrate that the phases of the final exfoliated TiS_2_, ZrS_2_, NbS_2_, MoS_2_, VS_2_, and TaS_2_ nanosheets are 1T, amorphous, H, 1T’ and 2H, 1T, and 1T, respectively (Fig. 5h–n, Fig. S36). XPS and Raman (Fig. S37) further confirmed the results (see Note S5 for details). These results indicate that phase transitions also appear in the exfoliation process (from LiMS_2_ to MS_2_ nanosheets), including 1T-to-1T (transition-free) in TiS_2_, 1T-to-amorphous in ZrS_2_, 3R-to-H in NbS_2_, 1T-to-1T’ in MoS_2_, 2H-to-1T in VS_2_ and TaS_2_. To further confirm the amorphous ZrS_2_, SAED patterns (Fig. S36g) were collected from three regions of the ZrS_2_ nanosheets, all of which show a characteristic diffuse ring, which is a hallmark of amorphous. This provides a more statistically significant observation compared to a single HRTEM image.

Exfoliation involves a chemical reaction [24] and an ultrasonication. Chemical reactions couple electron transfer with H_2_ generation, where the produced H_2_ exerts a driving force within the TMDs interlayers, and separates adjacent layers. Ultrasonication generates intense mechanical stress and shear forces, which can disrupt the crystal lattice of the material, potentially leading to a phase change (strain effects) [25,26]. Sonication can also cause local heating due to the absorption of sound energy by the material, thus providing the energy needed for a phase transition in the material (thermal effects) [27]. In addition, the energy imparted by ultrasonication can create defects and alter the microstructure of the material, which can destabilize the existing phase and promote the formation of a new phase (defect effects) [28]. For TMDs with obvious phase transitions (such as MoS_2_ and TaS_2_), we can further distinguish the contribution from chemical reaction or ultrasonication by conducting control experiments, see Note S6 and Fig. S38.

## APPLICATIONS OF EXFOLIATED TMD NANOSHEETS

**Figure 6.**
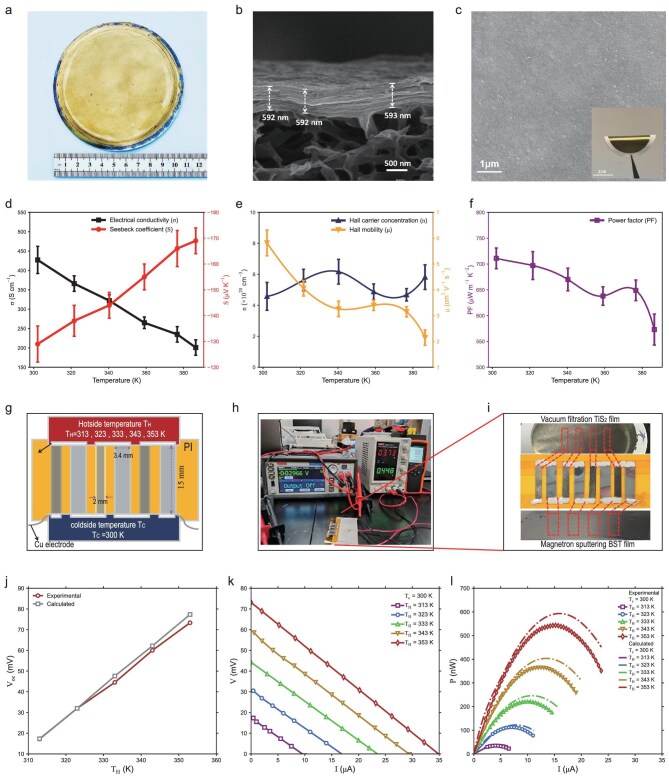
TiS_2_ nanosheet-based flexible thermoelectric generator (FTEG). (a) Picture of flexible TiS_2_ film. (b) Cross-sectional SEM image of the TiS_2_ film. (c) SEM image of the TiS_2_ film, the inset of which is a picture of the flexible TiS_2_ film. Temperature-dependent (d) electrical conductivity (σ) and Seebeck coefficient (*S*), (e) Hall carrier concentration (n) and Hall mobility (μ), as well as (f) power factor (PF) of the fabricated TiS_2_ film. (g) Schematic of the FTEG. (h) The homemade system for testing output performance of the FTEG. (i) Enlarged photo of the films, showing four pairs of p-type Bi_0.4_Sb_1.6_Te_3_ (BST) film and n-type TiS_2_ film as legs. (j) Open-circuit voltage (Voc) versus hotside temperature (*T_H_*). (k) Current-voltage (I-V) and (l) Current-power (I-P) curves of the flexible p-type BST and n-type TiS_2_ FTEG under various temperature differences (coldside temperature (*T_C_*), *T_C_* = 300 K; *T_H_* = 313 K, 323 K, 333 K, 343 K, and 353 K). *T_H_*: hotside temperature. *T_C_*: coldside temperature. PI: polyimide.

Our exfoliated atomically thin TMD nanosheets with diverse phases are compatible with solution-based deposition techniques—drop-casting, spin-coating, roll-to-roll coating, vacuum filtration, and so on—which make them easy for scalable manufacture of various electronics, photonics, optoelectronics, magnetic, and thermoelectric devices (see Table S3 for detailed application outlooks) [16,29–31]. As a proof-of-concept application, we presented the development of a thermoelectric device using of our exfoliated TiS_2_ nanosheets. We first prepared TiS_2_ nanosheet-based film on a flexible nylon or PVDF substrate using a vacuum filtration method (see Methods for details). The film exhibited a distinct layered structure with no apparent particle impurities, showing a flat surface with a pronounced metallic luster, good flexibility, and large size (with diameter of 10 cm) (Fig. 6a–c). Compared with the organic-intercalated layered TiS_2_, which is limited by costly synthesis, intercalant residue, and a rigid superlattice structure that caps its thermoelectric performance [32,33], our method can enable gram-scale production of the TiS_2_, which displays excellent stability as shown in Fig. S39 (also see Note S7 for the stability of TiS_2_ films) and high electrical conductivity.

Next, we measured the temperature-dependent thermoelectric properties of the prepared TiS_2_ nanosheet-based film from 300 to ∼380 K (Fig. 6d–f). Figure 6d illustrates its temperature-dependent electrical conductivity (*σ*) and Seebeck coefficient (*S*). The measured room temperature *σ* is 427 S·cm^−1^, which is two or four times higher than that of other reported TiS_2_-based film (Table S4) [34,35]. = *σ* increases with the increasing temperature, showing a typical metal behavior [36]. All the *S* are negative, indicating n-type conduction. While the absolute values of *S* present an increasing trend from –129 to –169 μV·K^−1^ with increasing temperature. Temperature-dependent Hall measurements (Fig. 6e) reveal a high room temperature carrier concentration of 4.6 × 10^20^ cm^−3^. It should be noted that at such high carrier concentrations, the material likely operates in a strongly degenerate state, where the Fermi level resides deep within a region of high density of states. Under these conditions, carrier transport is primarily governed by energy-dependent scattering within extended states, rather than by mobility-edge hopping or the non-degenerate diffusion described by Mott’s theory [37]. The observed reduction in conductivity arises entirely from mobility degradation dominated by acoustic phonon scattering (μ ∝ *T*⁻^2.1^), as the carrier concentration n remains invariant with temperature. This behavior is comparable to that observed in TiS_2_ single crystals [38]. Simultaneously, the enhancement in the Seebeck coefficient (*S*) is consistent with the generalized Mott formulation for degenerate systems [39]. In this regime, the energy derivative of conductivity is amplified due to spectral broadening induced by phonon scattering, which intensifies with increasing temperature. This mechanism—validated through Hall-effect measurements confirming the temperature stability of n, analysis of scattering rates, and agreement with degenerate semiconductor theory—reveals a fundamental transport characteristic of high-density carriers under inelastic scattering. The deviation from classical Mott behavior therefore underscores the departure from disordered or hopping-dominated transport, highlighting instead the dominant role of phonon-mediated energy-filtering effects in degenerate conductors [40]. Additionally, Fig. 6f shows a peak power factor of ∼710 μW·m^−1^·K^−2^ at 300 K in the TiS_2_ film, which is comparable with that of many other reports (Table S4) [32,41].

Further, we fabricated an eight leg flexible thermoelectric generator (FTEG) (see Fig. 6g for its scheme) using our developed TiS_2_ nanosheet-based flexible film with the help of high-performance *p*-type Bi_0.4_Sb_1.6_Te_3_ (BST) film (see Fig. S40 for the cross-section SEM image of BST film) deposited via the magnetron sputtering method [42,43]. The length, width, and thickness of BST and TiS_2_ films were selected as 15 mm × 3.4 mm × 447 nm and 15 mm × 2 mm × 592 nm (the thicknesses were measured by cross-section SEM images as shown in Fig. S40 and Fig. 6b). It is difficult to measure the in-plane thermal conductivity of the flexible film correctly, as we have tried Time-domain Thermoreflectance (TDTR) and Transient Electrothermal (TET) techniques, but both methods failed. Hence, we assume that our film has similar in-plane thermal conductivity as TiS_2_ and BST films as reported in Refs [44,45].

We measured the output performance—including open-circuit voltage (*V_oc_*) and maximum output power (*P*_max_)—of our fabricated FTEG on a homemade system (Fig. 6h, the enlarged photo is shown in Fig. 6i with physical photos of the corresponding films). The *V_oc_* is calculated by Eq. (1)


(1) \begin{eqnarray*}
{V}_{oc} = \frac{N}{2}\left(|{S}_p| + |{S}_n|\right)\Delta T,
\end{eqnarray*}

where *S_p_* is the *S* of *p*-type BST film, *S_n_* is the *S* of *n*-type TiS_2_ film, ∆*T* is temperature difference, and *N* is numbers of *p*-*n* pairs of legs [46]. Results show that a high voltage of 17.4, 30.8, 44.1, 58.7, and 73.4 mV were observed at *T*_H_ of 313, 323, 333, 343, and 353 K (Fig. 6j). The measured *V_oc_* of the flexible FTEG increases linearly with increasing hotside temperature (*T*_H_), consistent with the calculated results (black squares) based on the absolute *S* of TiS_2_ film (shown in Fig. 6d), the number of legs, and temperature difference [47]. Figure 6k and l, respectively, depicts the device working voltage and output power as a function of electrical current measured at ∆*T* from 13 K to 53 K. The *P*_max_ of the FTEG is determined by Eq. (2) [48]:


(2) \begin{eqnarray*}
P = {\left(\frac{{{V}_{oc}}}{{{R}_{{\mathop{\mathrm{int}}} } + {R}_{\textit{load}}}}\right)}^2{R}_{\textit{load}},
\end{eqnarray*}

in which *R*_int_ is the interface resistance of contacts, metal electrode, and legs on single unit of the device; *R_load_* is the load resistance of the device. A *P*_max_ of the FTEG was achieved at 543 nW which can be obtained at the ∆*T* of 53 K. The output voltage (*V*) and output power (*P*) under different ∆*T* of our FTEG were simulated by the finite element method [49]. Due to the presence of interface resistance, the actual effective resistances become higher and decreased the output power of our samples [50]. Still, there is only ∼8% loss in the measured *P*_max_ compared to the calculated value, indicating that our developed TiS_2_ flexible film possesses high thermoelectric performance and has the potential to be used in wearable electronics.

Furthermore, we fabricated and tested thermoelectric devices comprising five pairs of n-type TiS_2_ and p-type Bi_0.4_Sb_1.6_Te_3_ (fabricated via magnetron sputtering). Using hot water as a heat source, we investigated the output power with the temperature gradients at 10 K, 20 K, 30 K, 40 K, and 50 K (see Fig. S41a). Under a sustained temperature difference of ∼30 K, the devices exhibited excellent voltage stability during a continuous 6-hour test, with no significant degradation in output voltage (see Fig. S41b). These experiments collectively validate the operational reliability of the TiS_2_-based devices under controlled working conditions, highlighting their potential for practical applications.

## CONCLUSION

This Li^+^ intercalation chemistry work discovers rich emerging structural phase transitions beyond 2H-to-1T/1T’ in TMDs during electrochemical lithium intercalation (transition-free in TiS_2_ and ZrS_2_, 2H-to-3R in NbS_2_, as well as 1T-to-2H in VS_2_ and TaS_2_) and exfoliation (transition-free in TiS_2_, 1T-to-amorphous in ZrS_2_, 3R-to-H in NbS_2_, 2H-to-1T in VS_2_ and TaS_2_) (see Table S3) through state-of-the-art *in-situ*/*ex-situ* multimodal characterization techniques. Our work also reveals the general rules and mechanism for these phase transitions from the perspectives of structure, energy, and electron distribution (see Fig. S42 and Note S8). For Group IV (TiS_2_ and ZrS_2_) and Group Ⅴ (VS_2_ and TaS_2_) TMDs, the electron transfer resulted in the formation of half-filled and fully-filled LiMS_2_ states. This electron transfer reduced the free energy of the 2H phase. With Group IV TMDs, it reduced the free energy gap between the 1T and 2H phases without causing a phase change. In contrast, Group Ⅴ TMDs switched the stable phase from 1T to 2H. For Group Ⅵ (MoS_2_) TMDs, after lithium intercalation, the 1T-LiMS_2_ phase became the favored energy state, with the ${{\mathrm{d}}}_{yz, xz, xy}$ (t_2g_) orbital achieving a half-filled d^3^ configuration. Subsequently, a stable phase switch from 2H to 1T occurred.

Potential applications of the exfoliated TMD (exampled by TiS_2_) nanosheets in flexible devices (instanced by a thermoelectric generator) are also demonstrated; the fabrication of flexible, large-sized (with diameter of 10 cm) TiS_2_ nanosheet-based film (exhibiting an exceptional power factor of ∼710 μW·m^−1^·K^−2^ at 300 K) and the development of a high performance TiS_2_ thermoelectric generator (achieving a maximum power density of 458.6 W·m^−2^ at a 53 K temperature difference) are presented. Overall, our study contributes to the advancement of knowledge and nanotechnology regarding the nanoscale phase transitions occurring during electrochemical lithium intercalation, and it offers valuable guidance for predicting, understanding, and controlling these transitions. Moreover, our results provide a valuable library of information for the design and development of electrochemical lithium intercalation-assisted exfoliation processes in TMDs for the scalable preparation of solution-processable 2D nanosheets.

## METHODS

The detailed methods can be found in the online Supplementary file.

## Supplementary Material

nwag165_Supplemental_File
