# Supplementary material for: Li+ intercalation chemistry on 2D transition metal dichalcogenides towards phase evolution, scalable production, and application
Source: Natl Sci Rev. 2026 Mar 17;13(13):nwag165. doi: 10.1093/nsr/nwag165 (PMC13343487; doi:10.1093/nsr/nwag165)
Supplement: nwag165_Supplemental_File [file nwag165_supplemental_file.pdf]

Supporting information

for

Li^+ intercalation chemistry on 2D transition metal dichalcogenides towards phase evolution, scalable production and application

Qingyong Zhang[1]†, Jian Jiang[1,2]†, Ruixin Yan[1]†, Ruijie Yang[1,3]†, Xiaodong Wang[4]*, Qi Wang[1], Zhen Zhang[1,5], Qinghua Zhang[6], Qutong Yang[1],Qingyu Dong[7], Yu Tang[8], Ting Ying [1], Long Zheng [9], Shuaihang Hou[10], Yanbin Shen[7], Furong Chen[1], Ye Chen[9], Qi Liu[8], M. Danny Gu[10], Lin Gu[6], Lain-Jong Li[11], Qian Zhang[4], Xiao Cheng Zeng[1]*, Kian Ping Loh[12]*, Zhiyuan Zeng[1,2]*

[1]Department of Materials Science and Engineering, and State Key Laboratory of Marine Environmental Health, City University of Hong Kong, 83 Tat Chee Avenue, Kowloon, Hong Kong 999077, P. R. China

[2]Shenzhen Research Institute, City University of Hong Kong, Shenzhen 518057, China

[3]Department of Chemical and Petroleum Engineering, University of Calgary, 2500 University Drive, NW, Calgary, Alberta, T2N 1N4, Canada

[4]School of Materials Science and Engineering Institute of Materials Genome & Big Data Harbin Institute of Technology Shenzhen 518055, P. R. China

[5]Department of Materials Science and Engineering, Southern University of Science and Technology, Shenzhen, 518055, China

[6]Institute of Physics, Chinese Academy of Sciences, Beijing 100190, China

[7]i-Lab, CAS Center for Excellence in Nanoscience, Suzhou Institute of Nano-Tech and Nano-Bionics, Chinese Academy of Sciences, Jiangsu, Suzhou, 215123, China

[8]Department of Physics, City University of Hong Kong, 83 Tat Chee Avenue, Kowloon, Hong Kong 999077, P. R. China

[9]Department of Chemistry, The Chinese University of Hong Kong, Shatin, New Territories, Hong Kong 999077, P. R. China

[10]Hebei Key Lab of Optic-electronic Information and Materials, The College of Physics Science and Technology, Hebei University, Baoding 071002, P. R. China

[11]Eastern Institute for Advanced Study, Eastern Institute of Technology, Ningbo, Zhejiang 315200, P. R. China

[12]Department of Mechanical Engineering, University of Hong Kong, Hong Kong, P. R. China

[13]Department of Chemistry, National University of Singapore, Singapore 117543, Singapore

†Q. Y. Zhang, J. Jiang, R. X. Yan, and R. J. Yang contributed equally to this work.

*Corresponding Authors: zhiyzeng@cityu.edu.hk (Z. Zeng); chmlohkp@nus.edu.sg (K. LOH); xzeng26@cityu.edu.hk (X. C. Zeng); wangxiaodongjlu@163.com (X. Wang).

This file includes:

Methods

Supplementary Note 1 to 8

Supplementary Figures 1 to 42

Supplementary Tables 1 to 4

References 1-62

Table of Content

Fig. S1 | Lithium intercalation into bulk TMDs during a discharge process

Fig. S2 | Morphologies and structures of bulk TMDs

Fig. S3 | In-situ XRD cell

Fig. S4 | In-situ Raman cell

Fig. S5 | Selected XRD pattern of Li_xTiS_2 during in-situ and ex-situ test

Fig. S6 | In situ XRD patterns of TiS_2 during the electrochemical lithium intercalation

Fig. S7 | Lattice parameters evolution of Li_xTiS_2 during electrochemical lithium intercalation

Fig. S8 | Total energy of TMDs and lithium intercalated TMDs

Fig. S9 | Selected XRD pattern of Li_xZrS_2 during in-situ test

Fig. S10 | In situ XRD patterns of ZrS_2 during the electrochemical lithium intercalation

Fig. S11 | Lattice parameters evolution of Li_xZrS_2 during electrochemical lithium intercalation

Fig. S12 | Selected XRD pattern of Li_xNbS_2 during in-situ test

Fig. S13 | In situ XRD patterns of NbS_2 during the electrochemical lithium intercalation

Fig. S14 | Lattice parameters evolution of Li_xNbS_2 during electrochemical lithium intercalation

Fig. S15 | Selected XRD pattern of Li_xMoS_2 during in-situ and ex-situ test

Fig. S16 | 2D in situ XRD patterns of MoS_2 during the electrochemical lithium intercalation

Fig. S17 | Lattice parameters evolution of Li_xMoS_2 during electrochemical lithium intercalation

Fig. S18 | Selected XRD pattern of Li_xVS_2 during in-situ test and ex-situ test

Fig. S19 | In situ XRD patterns of VS_2 during the electrochemical lithium intercalation

Fig. S20 | Evolution of (001)/(002), (101)/(102), and (110) reflections of the selected in-situ XRD

patterns during the electrochemical lithium intercalation

Fig. S21 | Interlayer spacing evolution of Li_xVS_2 during electrochemical lithium intercalation

Fig. S22 | Selected XRD pattern of Li_xTaS_2 during in-situ test and ex-situ test

Fig. S23 | Evolution of (00l)(l = 1,2,4,8) reflections corresponding to the typical discharge profile

Fig. S24 | Interlayer spacing evolution of Li_xTaS_2 during electrochemical lithium intercalation

Fig. S25 | Structural characterization of $Li_{0.3}TaS_2$ and $LiTaS_2$

Fig. S26 | Structure change before and after electrochemical lithium intercalation

Fig. S27 |The projected density of state (PDOS) of atoms and the crystal orbital Hamilton population (–COHP) of Metal-S bonds in 2H MS_2

Fig. S28 | The projected density of state (PDOS) of atoms and the crystal orbital Hamilton population (–COHP) of Metal-S bonds in 1T/3R MS_2

Fig. S29 | The projected density of state (PDOS) of atoms and the crystal orbital Hamilton population (–COHP) of Metal-S bonds in 2H $LiMS_2$

Fig. S30 | The projected density of state (PDOS) of atoms and the crystal orbital Hamilton population (–COHP) of Metal-S bonds in 1T/3R $LiMS_2$

Fig. S31 | The d-orbitals for trigonal prismatic coordination (D3h symmetry) and octahedrally coordinated (D3d symmetry) in d-metal dichalcogenides

Fig. S32 | The effect of lithium intercalation on the metal-S and Li-S bond strength in TMDs

Fig. S33 | SEM images of exfoliated TMDs

Fig. S34 | Scalable production of TMDs

Fig. S35 | Dispersion of MS_2 nanosheets prepared by electrochemical lithium intercalation-assisted exfoliation of TMDs

Fig. S36 | Structures of single/few-layer MS_2

Fig. S37 | Raman and XPS of single/few-layer MS_2

Fig. S38 | Raman of 2D MoS_2 and TaS_2 exfoliation from $LiMoS_2$ and $LiTaS_2$ in H_2O with or without ultrasonication

Fig. S39 | The time-dependent stability of TiS_2 films

Fig. S40 | BST film

Fig. S41 | Performance of flexible thermoelectric devices

Fig. S42 | Schematic of the phase transition mechanism of TMDs

Table S1 | Lattice parameters of TMDs with and without Li intercalation obtained from DFT calculations.

Table S2 | Bond lengths of TMDs with and without Li intercalation obtained from DFT calculations.

Table S3 | Phase of MS_2 before electrochemical intercalation (Bulk MS_2), after intercalation ($LiMS_2$), after exfoliation (Exfoliated MS_2), and the potential applications of the atomically thin MS_2

Table S4 | Comparison of the Room Thermoelectric Performance of the TMDs based composites.

METHODS

Electrochemical lithium intercalation in TMDs

The TiS_2 and MoS_2 powders were purchased from Aladdin (Aladdin, China), while the ZrS_2 and TaS_2 powders were obtained from 6 Carbon Technology (Shenzhen, China). The VS_2 and NbS_2 powders were prepared in-house. To prepare the electrode, a slurry was created by combining 80 wt% of the bulk TMDs, 10 wt% Super P (Imerys, France), and 10 wt% polyvinylidene difluoride (PVDF) (Sigma, China). The slurry was then coated onto a current collector, specifically a 15 μm aluminum foil (Anaqua Global International, Hong Kong, China), and dried under vacuum at 100°C for over 8 hours. The resulting dried electrode was punched into a 12 mm diameter disc with a bulk TMDs loading of approximately 2.0 mg. The CR2025 cells (Guangdong Canrd New Energy Technology Co., Ltd., China) were assembled in an argon-filled glovebox. The working electrode consisted of the TMDs electrode, while lithium foil (China Energy Lithium Co., Ltd) served as the counter electrode and reference electrode. The electrolyte used was 1 M LiPF6 in a mixture of ethylene carbonate (EC), ethyl methyl carbonate (EMC), and dimethyl carbonate (DMC) in a 1:1:1 volume ratio, sourced from Suzhou Duo Duo Chemical Technology. A polypropylene (pp) film (Celgard 2300) was used as the separator. Galvanostatic discharge was performed at room temperature using the Neware battery testing system (Neware, China) with a current of 0.1C (238.87 mA/g for TiS_2, 172.52 mA/g for ZrS_2, 170.67 mA/g for NbS_2, 167.4 mA/g for MoS_2, 232.89 mAh/g for VS_2, and 109.38 mA/g for TaS_2). The cut-off voltage values were set at 0.9V for TiS_2, ZrS_2, MoS_2, and TaS_2, 0.2V for NbS_2, and 1.6V for VS_2. Finally, the lithium intercalated TMDs were obtained.

Exfoliation for the preparation of TMD mono- or few-layers

A dispersion containing single/few-layer TMDs is prepared by subjecting lithium-intercalated TMDs

to water (approximately 20 mL) and simultaneous sonication for approximately 15 minutes using an ultrasonic device (elmasonic, Elma Schmidbauer GmbH, Germany). To separate the single/few-layer TMDs from impurities and unexfoliated TMDs, a two-step centrifugation process is employed. In the first step, the dispersion is centrifuged at 3000 rpm for 15 minutes, repeated twice, using a centrifuge (Sorvall, Thermo Scientific, Germany). This step allows the TMDs particles and impurities, such as conductive materials and PVDF, to settle at the bottom of the centrifuge tube and be discarded. Subsequently, the remaining supernatant is centrifuged at 8000 rpm for 15 minutes to collect the single/few-layer TMDs. The single/few-layer TMDs deposit at the bottom of the centrifuge tube. Prior to using the single/few-layer TMDs, it is necessary to wash the deposited material.

XPS tests

X-ray photoelectron spectroscopy (XPS) measurements were performed using the Thermo Fisher Scientific K-Alpha+ instrument (Thermo Fisher Scientific, USA) equipped with an Al Kα X-ray source (hυ = 1486.6 eV). Both bulk TMDs and exfoliated single/few-layer TMDs were directly transferred into the analysis chamber. The pressure within the analysis chamber was maintained below 2×10^{-7} mBar. The binding energy scale was calibrated using the C 1s peak at 284.8 eV, which served as a reference for hydrocarbon contamination.

Raman tests

Raman spectra of bulk TMDs and exfoliated TMDs were collected at room temperature using a Renishaw Raman microscope (Renishaw, UK) equipped with a ×50 objective lens, providing a lateral spatial resolution of approximately 1 μm. The excitation wavelength of 532 nm was employed. For exfoliated TMDs, a purified dispersion of TMDs nanosheets was deposited onto a SiO_2/Si substrate and allowed to dry naturally. In-situ Raman spectra measurements were conducted with the same

parameters as the ex-situ Raman test using a two-electrode Raman cell (Gauss Union, China) during galvanostatic discharge. The discharge was carried out at a rate of 0.2C, completing the test in approximately 5 hours. The Raman measurements were performed using an Ivium electrochemical workstation (Ivium Technologies, Netherlands).

XRD tests

Ex-situ XRD data of bulk TMDs were collected using a Bruker D8 diffractometer (Bruker, USA) with Cu-Kα radiation in the 2θ range of 10° to 80°. The generator was operated at 30 kV and 10 mA. In-situ XRD measurements were performed during galvanostatic discharge at a rate of 0.1 C using a two-electrode in-situ XRD cell (Beijing scistar technology, China). The measurements were conducted with the same parameters as the ex-situ XRD test. The galvanostatic discharge was carried out using the Neware battery testing system.

SEM, TEM and HAADF-STEM tests

SEM images of the bulk TMDs were acquired using a Scanning Electron Microscope (SEM) model FEI/Philips XL30 Esem-FEG (USA). Transmission Electron Microscopy (TEM) images were captured using JEOL-2100F transmission electron microscopes (JEOL Ltd., Japan). High-angle annular Dark-Field Scanning Transmission Electron Microscopy (HAADF-STEM) images were obtained using a JEOL JEM-ARM200F instrument (JEOL Ltd., Japan).

Preparation of VS_2

VS_2 was synthesized using a hydrothermal method. In a typical procedure, 2 mL of ammonium hydroxide ($NH_3 \cdot H_2O$) was dispersed in 40 mL of deionized water and stirred for 5 minutes to obtain a homogeneous solution. Then, 0.234 g of ammonium metavanadate (NH_4VO_3) powder was added to the solution and stirred for 10 minutes until a uniform, transparent solution was formed. Subsequently,

1.5 g of thioacetamide (C_2H_5NS) powder was added and stirred for 1 hour at room temperature. The resulting brown dispersion was transferred into a Teflon-lined autoclave and maintained at 180 °C for 24 hours. Afterward, the product was collected, washed multiple times with deionized water and ethanol, and dried at 80 °C under vacuum. Finally, the VS_2 nanosheets were obtained by subjecting the dried product to heat treatment under an argon atmosphere at 300 °C for 2 hours.

Preparation of NbS_2

NbS_2 was prepared by mixing and grinding stoichiometric amounts of niobium and sulfur powders, which were then placed and sealed under high vacuum conditions. Subsequently, the sample was placed in a tube furnace and heated to 900 °C for 10 hours with a heating rate of 5 °C/min. Once the sample had cooled to room temperature, bulk NbS_2 was obtained.[1]

Fabrication of flexible thermoelectric generator

The TiS_2 nanosheet-based film was fabricated through vacuum filtration. The suspension of exfoliated TiS_2 nanosheets was filtered over flexible nylon or PVDF with 220 nm pore sizes, and dried in Vacuum for 12 h at 60 ºC. An eight-leg flexible TE generator was assembled by connecting four *n*-type TiS_2 film and four *p*-type $Bi_{0.4}Sb_{1.6}Te_3$ (BST) film in series with silver paste. The BST films were deposited via megnetron sputtering method on polyimide (PI) according to the reference.[2] The size of each leg is 15 mm × 3.4 mm × 447 nm for BST film and 15 mm × 2 mm × 592 nm for TiS_2 film. The hotside temperature (T_H) was set at 313, 323, 333, 343, and 353 K, while the coldsite temperture (T_C) was almost stayed at 300 K controlled by ambient temperature.

Measurement of flexible thermoelectric generator

The electrical conductivity and Seebeck coefficient were simultaneously measured from 300 to 383 K on a commercial ZEM-3 setup (Advance-Riko, Inc. Japan) based on the four-point DC current-

switching method and the static temperature difference method. The temperature dependent Hall coefficients (R_H) were measured using the van-der-Pauw technique under a reversible magnetic field of 1.5 T. The Hall carrier concentration (n) and Hall mobility (μ) were calculated from the relationship $n = 1/(e \cdot R_H)$ and $\mu = \sigma \cdot R_H$, respectively. The performance of flexible TE generator was characterized on a homemade system equipped with a commercial Peltier device for establishing the temperature difference and a digital source meter (Keithley 2400) for the voltage measurement. All simulations were performed using finite element analysis (Workbench platform).

Calculation methods

Spin-polarized density functional theory (DFT) calculations were performed using the Vienna Ab Initio Simulation Package (VASP), version 6.3.2, with the Projector-Augmented Wave (PAW) pseudopotential implementation.[3-5] The exchange and correlation effects of electrons were described by the generalized gradient approximation (GGA) functional of PBE.[6] To account for van der Waals interactions, Grimme's DFT-D3 method was applied.[7] A plane-wave basis set with a kinetic energy cutoff of 550 eV was utilized. The Brillouin zone was sampled using a Monkhorst-Pack k-point grid with dimensions of (13×13×3).[8] Convergence criteria were set at 10^{-5} eV and 10^{-3} eV/Å for electronic and ionic relaxations, respectively. The optimized structures of MS_2 and $LiMS_2$, with Li atoms placed at the octahedral site in $LiMS_2$, were used for electronic structure analysis and determination of the transition pathway. The solid-state nudged elastic band (ss-NEB) method was employed to determine the transition pathway.[9] Crystal orbital Hamilton population (COHP) and integrated COHP (ICOHP) analyses were performed using the LOBSTER program.[10-12]

The relative energy differences of various phases are calculated by the following equation:

$$\Delta E^{phase1-phase2} = E^{phase1} - E^{phase2}$$

Where E^{phase1} and E^{phase2} are the relative energy of the phase1 and phase2.

Supplementary Note 1: overall introduction of the phases of TMDs

TMDs exhibit diverse polytypes, with the most common bulk phases being 1T, 2H, and 3R. These polytypes arise from the stacking of S-M-S monolayers in different sequences,[13] with interlayer cohesion primarily governed by van der Waals forces.[14, 15] Among the thermodynamically stable monolayer TMDs, the T and H phases predominate. The T phase monolayer consists of MS_6 octahedra linked through edge-sharing interactions, while the H phase monolayer is composed of MS_6 trigonal prisms connected via edge-sharing interactions.[16] Specifically, the 1T phase corresponds to a single layer of T phase stacking in the AbC AbC sequence, the 2H phase comprises two layers of H phase stacking in the AbA BaB sequence, and the 3R phase consists of three layers of H phase stacking in the AbA CaC BcB sequence.[17]

Supplementary Note 2*:* properties of bulk TMDs

Properties of bulk TiS_2. For bulk TiS_2, XPS measurements (**Fig. S2a (II)**) exhibit doublet peaks in the Ti 2p spectra of 1T TiS_2 and TiO_2, indicative of surface oxidation of the bulk 1T TiS_2. XRD analysis (**Fig. S2a (III)**) demonstrates the high crystallinity of bulk TiS_2 in an octahedral symmetry, corresponding to the 1T polytype (space group $P\bar{3}m1$). However, the trace amount of oxidation does not impact the results. Raman spectra (**Fig. S2a (IV)**) confirm the 1T polytype of bulk TiS_2, exhibiting two active modes of A_{1g} and E_g located at approximately 332 and 230 cm^{-1}, respectively.[18, 19]

Properties of bulk ZrS_2. For bulk ZrS_2, the XPS analysis (**Fig. S2b (II)**) of ZrS_2 exhibits the presence of impurities in the 1T phase, which is consistent with the XRD pattern (**Fig. S2b (III)**). Raman spectroscopy (**Fig. S2b (IV)**) only displays the characteristic features of ZrS_2, indicating the predominance of the 1T phase in ZrS_2.

Properties of bulk NbS_2. For bulk NbS_2, the deconvolution of the XPS spectrum for bulk NbS_2 (**Fig. S2c (II)**) demonstrates the coexistence of the 2H and 3R phases. The XRD analysis of bulk NbS_2 (**Fig. S2C (III)**) reveals the presence of the 2H phase (space group $P6_3/mmc$) with trigonal prismatic symmetry. Raman spectroscopy (**Fig. S2c (IV)**) shows three vibrational modes of E_{1g}, E_{2g}^1 and A_{1g} located at approximately 262, 305, and 378 cm^{-1}, respectively, confirming the 2H phase of bulk NbS_2. The 3R NbS_2 is difficult to detect using XRD and Raman techniques due to its small quantity.

Properties of bulk MoS_2. For bulk MoS_2, XPS (**Fig. S2d (II)**) of MoS_2 illustrates the 2H features. XRD in **Fig. S2d (III)** exhibits bulk MoS_2 in the 2H phase, displaying trigonal prismatic symmetry with a space group of $P6_3/\,mmc$. The confirmation of the 2H phase is also supported by Raman spectroscopy, which identifies the active modes of A_{1g}, E_{2g}^1 and E_{1g} at 408, 381, and 286 cm^{-1}, respectively (**Fig. S2d (IV)**).[20, 21]

Properties of bulk VS_2. Bulk VS_2 exhibits a flower-like morphology (**Fig. S2e (I)**), and the deconvolution of V 2p spectra (**Fig. S2e (II)**) reveals two doublets, indicating V^{4+} in VS_2 and low-valence V^{2+} species attributed to S-vacancies on the surface.[22] Its XRD (**Fig. S2e (III)**) and Raman (**Fig. S2e (IV)**) analyses confirm the presence of the 1T phase.[23, 24]

Properties of bulk TaS_2. The SEM image of layered bulk TaS_2 is shown in **Fig. S2f (I)**. The deconvolution of Ta 4f spectra in XPS (**Fig. S2f (II)**) implies the slight oxidation of 1T TaS_2.[25] The bulk TaS_2 is the 1T phase in the trigonal space group $P\bar{3}m1$, which is confirmed by XRD (**Fig. S2f (III)**). Raman spectroscopy shows three active vibrational modes, namely E_{1g} (241 cm^{-1}), E_{2g}^1 (307 cm^{-1}), and A_{1g} (380 cm^{-1}),[26, 27] indicating the 1T phase of TaS_2, which is consistent with the XRD results.

Supplementary Note 3: in-situ Raman analysis of TaS_2 during lithiation

In the *in-situ* Raman spectra of TaS_2 (**Fig. 3f)** during lithium intercalation, a peak at 120 cm^{-1} emerges, which can be assigned to the out-of-plane excitation of the lithium monolayer. Subsequently, new emerging peaks at 136 cm^{-1} and 173 cm^{-1} may arise from the in-plane and out-of-plane excitation of the lithium monolayer. Besides, the E_{1g}peak at 242 cm^{-1} develops into a broad peak that may come from two-phonon mode and then splits into two peaks located at 250 and 270 cm^{-1}. The E_{2g}^1 peak splits into two peaks at 307 and 322 cm^{-1}, which are attributed to the TaS_2 in-plane bending mode. The peak position of the A_{1g}mode shifts to a lower Raman shift and is finally located at 373 cm^{-1}. The Raman evolution aligns with 1T-to-2H phase transformation induced by Fe intercalation into TaS_2,[28] Thus, the Raman double confirms the 1T-to-2H phase transition of TaS_2 during lithium intercalation.

Supplementary Note 4*:* electronic structure analyses

The electronic structure of TMDs is strongly influenced by the coordination environment of the transition metal and its *d*-electron count. We mainly discuss TiS_2, MoS_2 and TaS_2 in (**Fig. S27-S31**), since their transition metals belong to Group 4, 6, and 5, respectively, and possess different numbers of *d*-electrons. Additionally, a crystal orbital Hamilton population (COHP) analysis was performed to examine the chemical bonding in MS_2 and $LiMS_2$ systems. For bulk TiS_2 (d^0 configuration), the projected density of state (PDOS) and the crystal orbital Hamilton population (–COHP) show that its antibonding *d* orbital remained unoccupied, and the HOMO of both 1T-TiS_2 and 2H-TiS_2 is the bonding *d* orbital (**Fig. S28a**, **Fig. S27a**). Upon the lithium intercalation, the energy gap between 1T-$LiTiS_2$ and 2H-$LiTiS_2$ is reduced (**Fig. 4a**), as compared with the energy gap of 1T-TiS_2 and 2H-TiS_2, indicating a lower energy state for the d_{z^2} (a_1) orbitals to be half-filled with electron transferred from lithium in the 2H phase and a metallic characteristic of 2H-$LiTiS_2$ (**Fig. S29a)**. However, the energy

gap between 1T-$LiTiS_2$ and 2H-$LiTiS_2$ is still negative (**Fig. 4a**), meaning that the lithium intercalation TiS_2 is transition-free. In the case of bulk MoS_2 (Group 6) with d^2 configuration, the fully occupied d_{z^2} (a_1) orbital in the 2H phase was found to be energetically favorable compared to the partially occupied degenerate $d_{yz,xz,xy}$ (t_{2g}) orbital (**Fig. S27d**). However, upon Li intercalation, the additional electron donated by Li atoms occupied the highest occupied molecular orbital (HOMO), which is $d_{x^2-y^2,xy}$ (e) for 2H-$LiMoS_2$, and $d_{yz,xz,xy}$ (t_{2g}) for 1T-$LiMoS_2$, while half filled $d_{yz,xz,xy}$ (t_{2g}) in 1T phase $LiMoS_2$ is more favorable than 2H-$LiMoS_2$ (**Fig. S30d, Fig. S29d**), indicating a switch of the stable phase from 2H to 1T phase. For TaS_2 (Group 5 transition metal) with d^1 configuration, the energy levels of the 1T and 2H phases were found to be comparable (**Fig. 4a**). Upon the introduction of an electron by the lithium atom, the 2H phase became even more energetically favorable (free energy reduced) as the d_{z^2} (a_1) orbital became fully occupied, which is consistent with the presence of occupation of d_{z^2} (a_1) orbital near the Fermi level in PDOS (**Fig. S29f**).

Supplementary Note 5: properties of exfoliated TMD nanosheets

Single or few-layer TiS_2 retained the 1T phase with relatively decreased crystallinity (**Fig. S36a**) and slight oxidation (**Fig. S37a (I)**). The 1T phase is also determined by XPS and Raman in **Fig. S37a** and STEM-HAADF image in **Fig. 5h**, but. XPS (**Fig. S37b (I)**) implies the slight oxidation of the exfoliated ZrS_2 nanosheets, and Raman spectroscopy of exfoliated ZrS_2 (**Fig. S37b (II)**) differed from that of bulk ZrS_2 (**Fig. S2b (IV)**), due to the amorphous structure of exfoliated ZrS_2 (**Fig. 5i, Fig. S37b**), which indicates the destruction of long-range order structure during exfoliation. XPS and Raman analyses of exfoliated NbS_2 (**Fig. S37c**) show characteristics of the 2H and 3R phases, which consist of monolayer H phase NbS_2. The STEM-HAADF image reveals the presence of the H phase of NbS_2 (**Fig. 5j, Fig. S36c**). Thus the STEM-HAADF, XPS and Raman agree well with each other.

Exfoliated MoS_2 consists of single or double layers (**Fig. S37d**) and exhibits a co-existence of 1T' and 2H phases (**Fig. 5k-l, Fig. S36d**). The feature of a zigzag chain of Mo atoms in the 1T' phase is illustrated in **Fig. 5l (I)**. The deconvolution of XPS (**Fig. S37e (I)**)) demonstrates that the spectra with the V 2p3/2 and V 2p1/2 in 1T VS_2 located at 516.75 eV and 524.45 eV, respectively (**Fig. S37e (I)**).[29] Raman (**Fig. S37e (II)**) also identifies the presence of 1T VS_2 nanosheets.[30] STEM-HAADF imaging in **Fig. 5m** double confirms the 1T phase of exfoliated VS_2. The 1T phase of exfoliated TaS_2 is determined by XPS and Raman in **Fig. S36f**. The exfoliated TaS_2 consists of single or double layers (**Fig. S37f**) and its 1T phase is double-confirmed by the STEM-HAADF image (**Fig. 5n**). The feature of a zigzag chain of Ta atoms in the 1T phase is illustrated in **Fig. 5n(I)**.

Supplementary Note 6: phase transition during exfoliation

$LiMoS_2$ and $LiTaS_2$ show obvious phase transition after exfoliation. We took them as examples to separate the contributions of chemical reaction and ultrasonication via exfoliating them in H_2O with or without ultrasonication in our work. The Raman of $LiMoS_2$ and the corresponding exfoliated MoS_2 are shown in **Fig. S38a**. It demonstrates that from $LiMoS_2$ to MoS_2 nanosheets, the phase transition is triggered by ultrasonication. Because the Raman data of MoS_2 nanosheets exfoliated from $LiMoS_2$ without ultrasonication didn’t show 1T phase. While **Fig. S38b** and **Fig. S38c** demonstrate Raman that from $LiTaS_2$ to TaS_2 nanosheets, the phase transition is triggered by the chemical reaction. Because the TaS_2 nanosheets exfoliated from $LiTaS_2$ with or without sonication both show phase transition to 1T phase. Thus, both chemical reactions and ultrasonic forces can induce phase transition during exfoliation, but their contributions differ across different TMDs. But for the ZrS_2, which exhibits a phase transition from 1T to amorphous, it is challenging to differentiate what factor contributed to its phase transition from 1T to amorphous, since ultrasonication is needed to obtain the 2D ZrS_2.

Supplementary Note 7: stability of TiS_2 films and the thermoelectric devices

Here, we measured the time-dependent resistance evolution of TiS_2 films under ambient atmospheric conditions. The result shows that the resistance variation is below 2% over a 6-hour period, see **Supplementary Fig. 39a**, which indicates reasonable short-term stability in air. Furthermore, the mechanical stability was assessed by attaching the films on glass tubes (radius = 5 mm). The resistance ratio of R/R_0 of TiS_2 film depends on the bending times shown in **Supplementary Fig. 39b**. We can see that after 1,000 bending cycles (radius = 5 mm), the resistance increases by only ~15%, with no observable TiS_2 peeled off from the film, which indicates excellent mechanical stability of the TiS_2 film.

Supplementary Note 8: phase evolution mechanism during lithium intercalation

The phase evolution mechanism upon lithium intercalation-assisted exfoliation is illustrated in **Fig. S42**. Lithium intercalates into TMDs via galvanostatic discharge to the cut-off voltage. The intercalated lithium expands the interlayer spacing of TMDs and facilitates the following exfoliation. The exfoliation of lithium intercalated TMDs can produce single/few-layer TMDs nanosheets on a large scale (**Fig. S34, Fig. S35** and **Fig. S42a**). Besides, electrochemical lithium intercalation also induces phase transition. The process of Li intercalation initiates an increase of Fermi-level in the electronic structure of $LiMS_2$ (Left panel of **Fig. S42b-d**), which influences the energy disparity of $LiMS_2$, thereby promoting the phase transition. For TMDs with Group 4 or Group 5 metals in **Fig. S42b, d**, the electrons contributed by lithium occupy the d_{z^2} orbital in the 2H phase of $LiMS_2$, resulting in a half-filled or fully-filled states. This redistribution of electrons motivates the energy reduction of 2H phase $LiMS_2$ into lower energy state, which in turn reduces the energy gap between 1T-$LiMS_2$ and 2H-$LiMS_2$ in Group 4 metals and decreases the energy gap, and which also enhances the relative stability

of 2H phase rather than 1T phase for Group 5 metal. In contrast, for TMDs with Group 6 metals in **Fig. S42c**, with the electron transfer from the valence orbital of Li to the d orbital of the transition metal center, 1T-$LiMS_2$ phase becomes the favored energy state when the $d_{yz,xz,xy}$ (t_{2g}) orbital becomes half-filled with a d^3 configuration, in this case, there is a switch of the stable phase from 2H to 1T phase.

Supplementary Figures and Tables

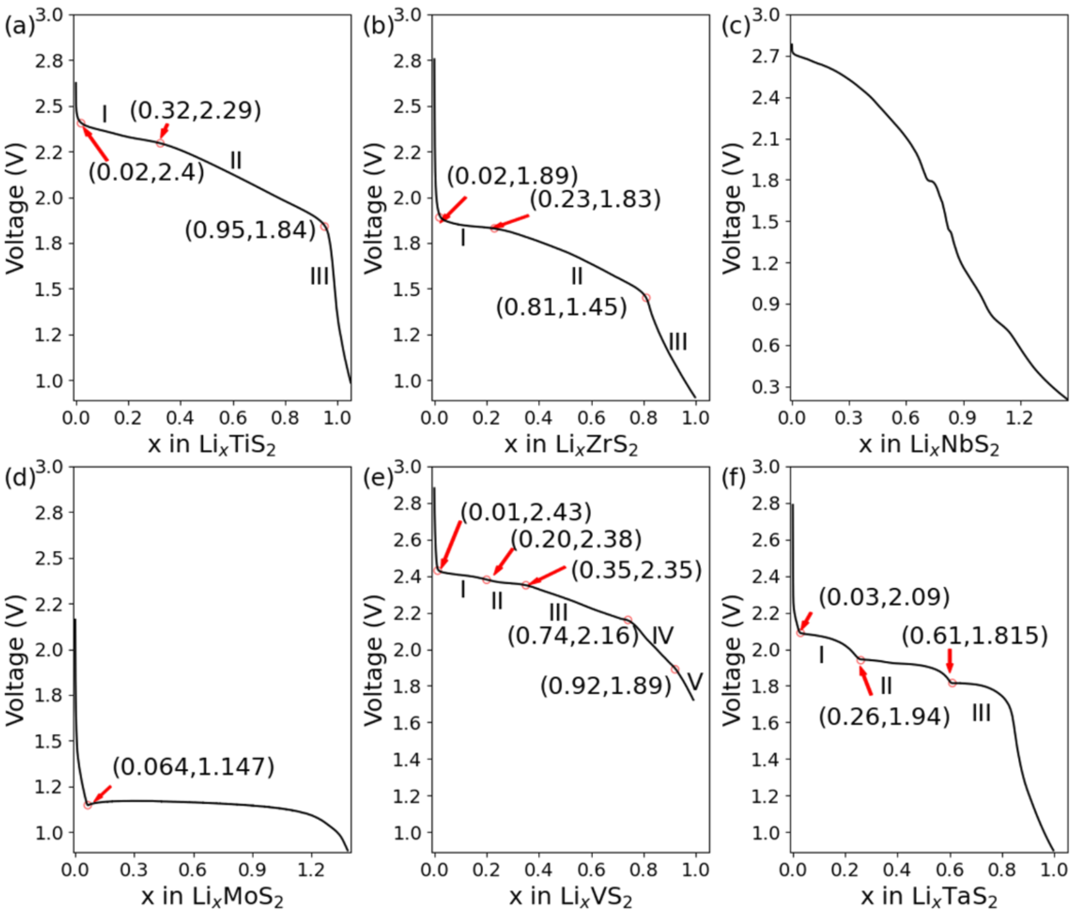

Fig. S1 | Lithium intercalation into bulk TMDs during a discharge process. (a) Discharge curve of TiS_2. (b) Discharge curve of ZrS_2. (c) Discharge curve of NbS_2. (d) Discharge curve of MoS_2. (e) Discharge curve of VS_2. and (f) Discharge curve of TaS_2. A plateau in the discharge curve (especially the OCV-x curve) means a two-phase region.[31, 32]

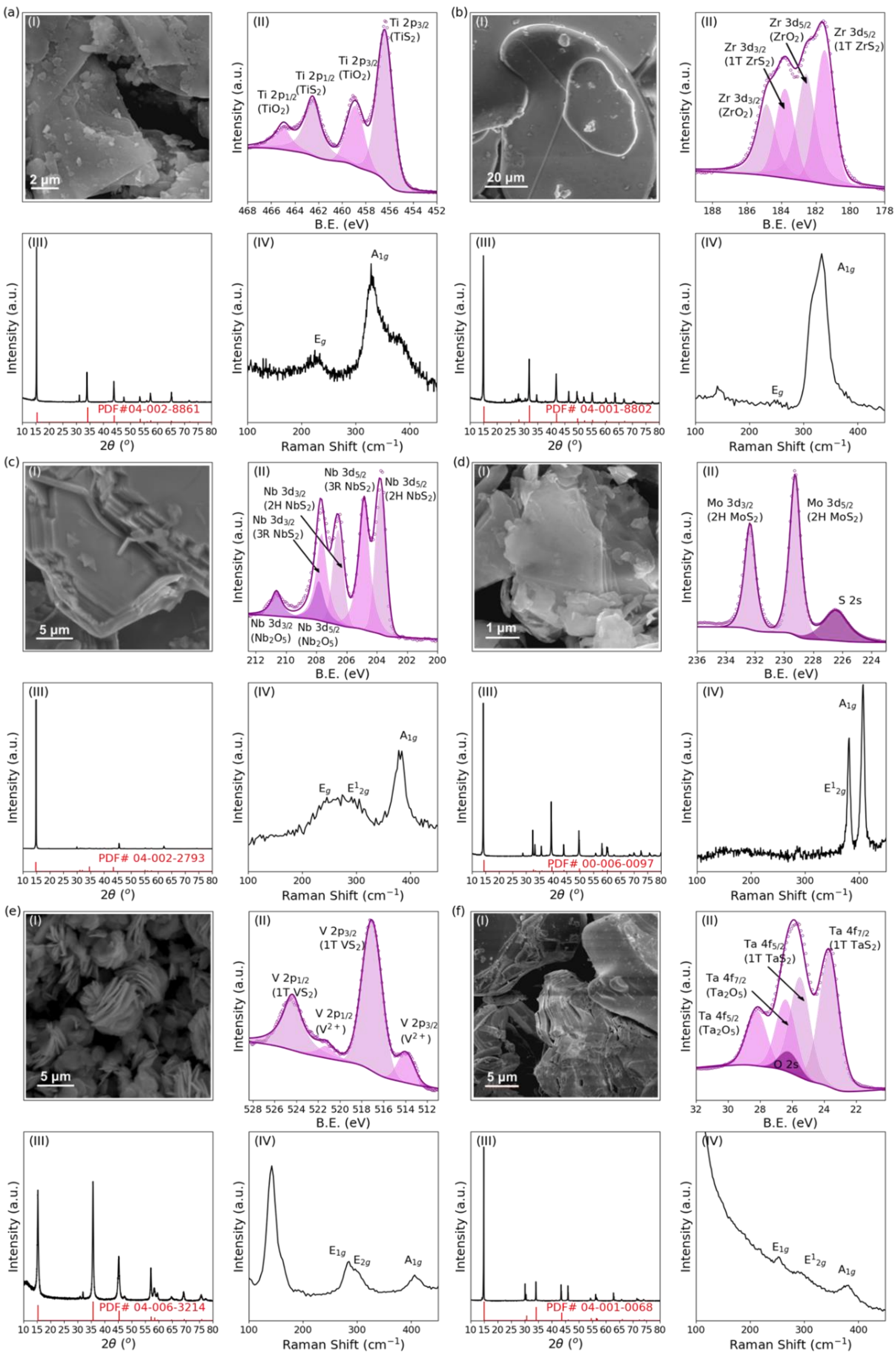

Fig. S2 | Morphologies and structures of bulk TMDs. (a) SEM image (I), XPS of transition metal (II), XRD pattern (III), and Raman spectra (IV) of TiS_2, (b) SEM image (I), XPS of transition metal (II), XRD pattern (III), and Raman spectra (IV) of ZrS_2, (c) SEM image (I), XPS of transition metal (II), XRD pattern (III), and Raman spectra (IV) of NbS_2, (d) SEM image (I), XPS of transition metal (II), XRD pattern (III), and Raman spectra (IV) of MoS_2, (e) SEM image (I), XPS of transition metal

(II), XRD pattern (III), and Raman spectra (IV) of VS_2, and (f) SEM image (I), XPS of transition metal (II), XRD pattern (III), and Raman spectra (IV) of TaS_2. All the TMDs have a layered structure. Bulk TiS_2 is the 1T phase. Bulk ZrS_2 is the 1T phase with some impurities. The presence of impurities does not interfere with the structural evolution of ZrS_2 during lithium intercalation. Bulk NbS_2 is the 2H phase. Bulk MoS_2 is the 2H phase. Bulk VS_2 is the 1T phase. And bulk TaS_2 is the 1T phase.

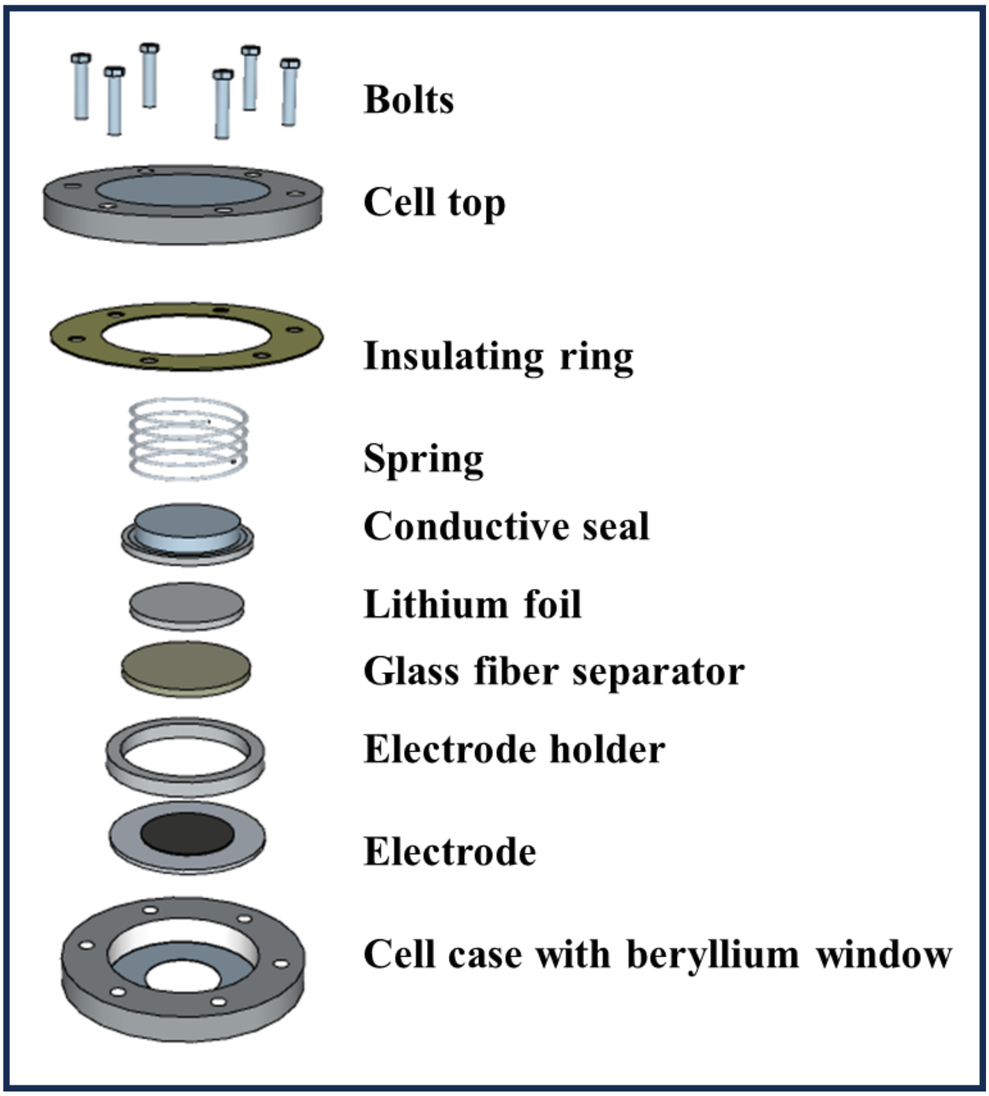

Fig. S3 | In-situ XRD cell.

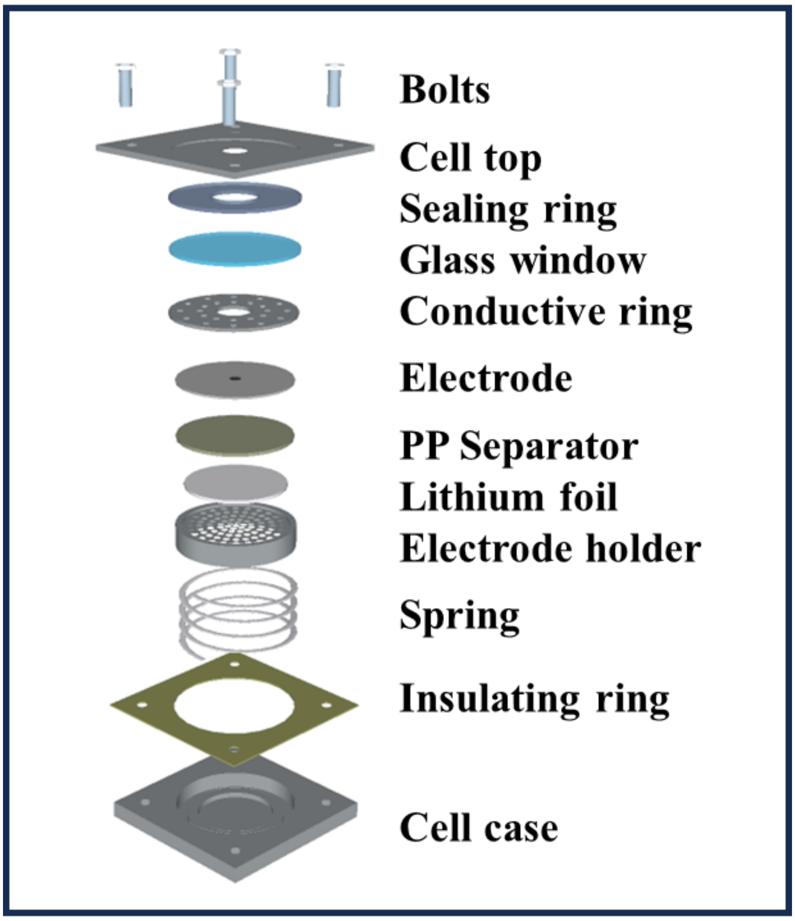

Fig. S4 | In-situ Raman cell.

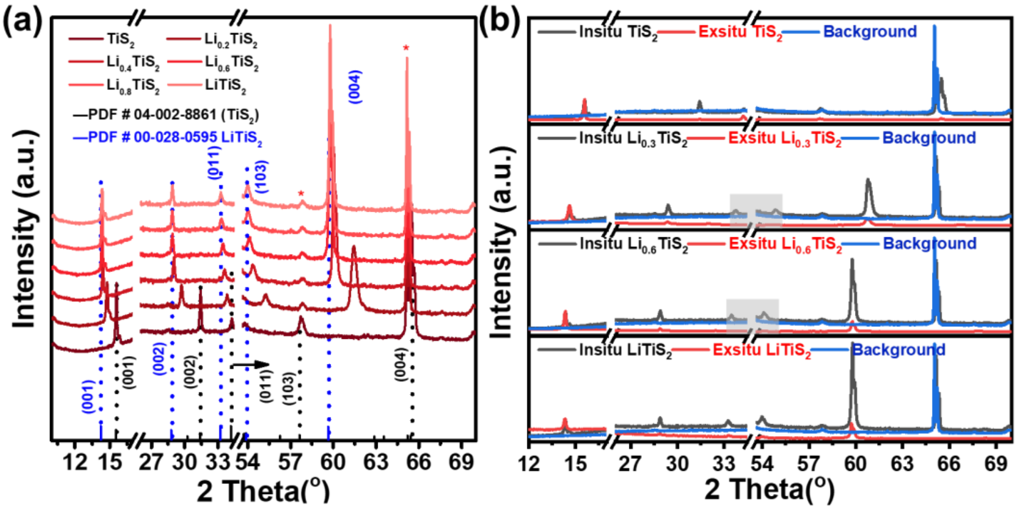

Fig. S5 | Selected XRD pattern of Li_xTiS_2 during in-situ and ex-situ test. (a) Selected in-situ XRD with index. (b) Comparison of in-situ and ex-situ XRD. During electrochemical lithium intercalation, 1T phase TiS_2 changes to 1T $LiTiS_2$.

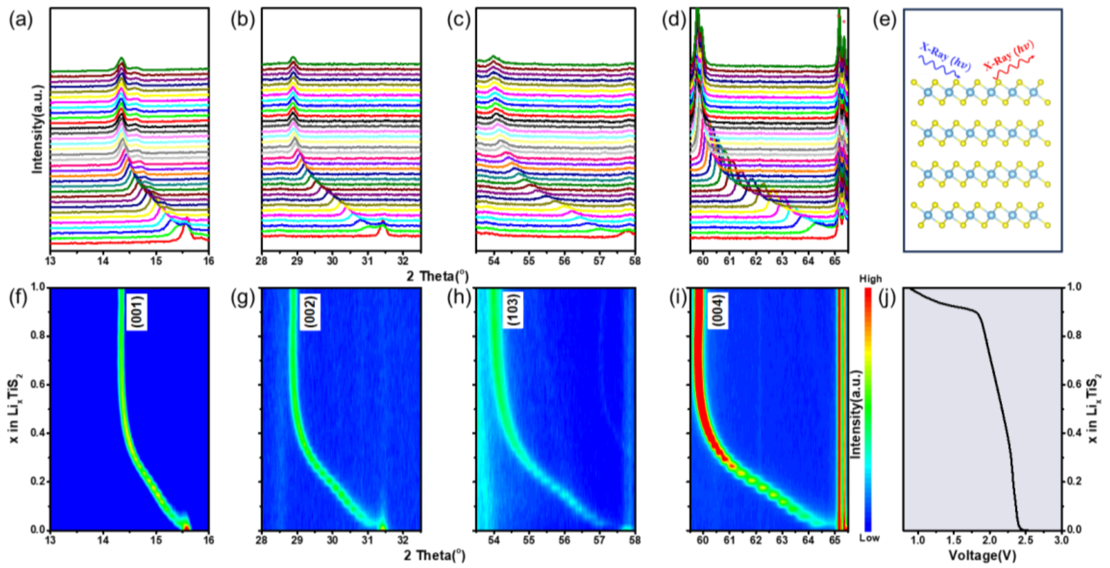

Fig. S6 | In situ XRD patterns of TiS_2 during the electrochemical lithium intercalation. (a) Stacked XRD pattern of (*001*) diffraction peak. (b) Stacked XRD pattern of (*002*) diffraction peak. (c) Stacked XRD pattern of (*103*) diffraction peak. (d) Stacked XRD pattern of (*004*) diffraction peak. (e) Schematic of diffraction of X-rays in TiS_2. (f)-(i) Countour plots corresponding to (a)-(d). The (00l) peaks shift to a lower angle, indicating the increase of the interlayer spacing. (j) Typical x-voltage profile.

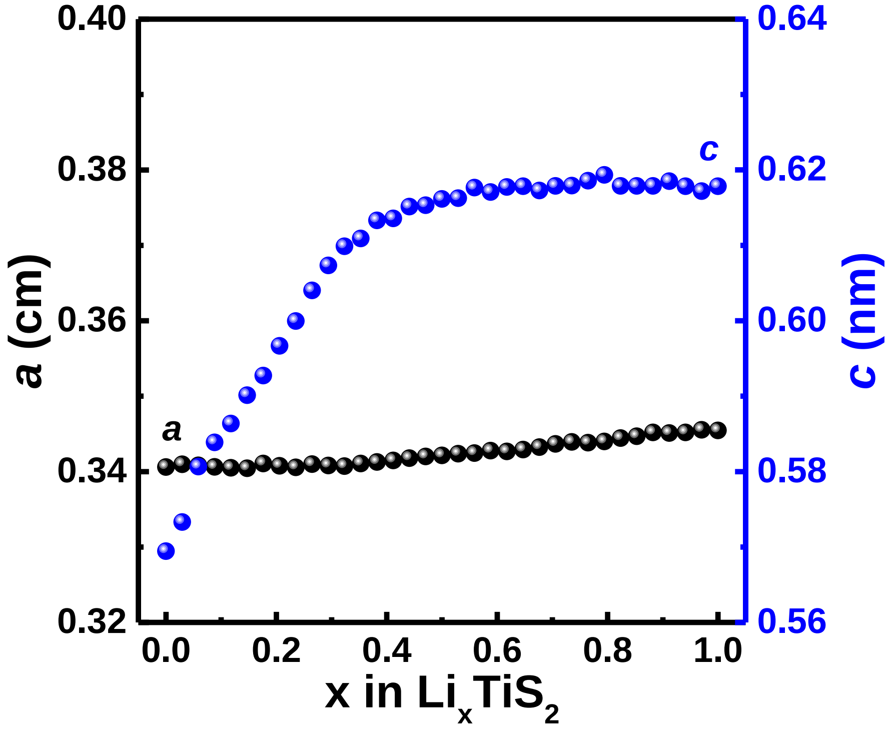

Fig. S7 | Lattice parameters evolution of Li_xTiS_2 during electrochemical lithium intercalation.

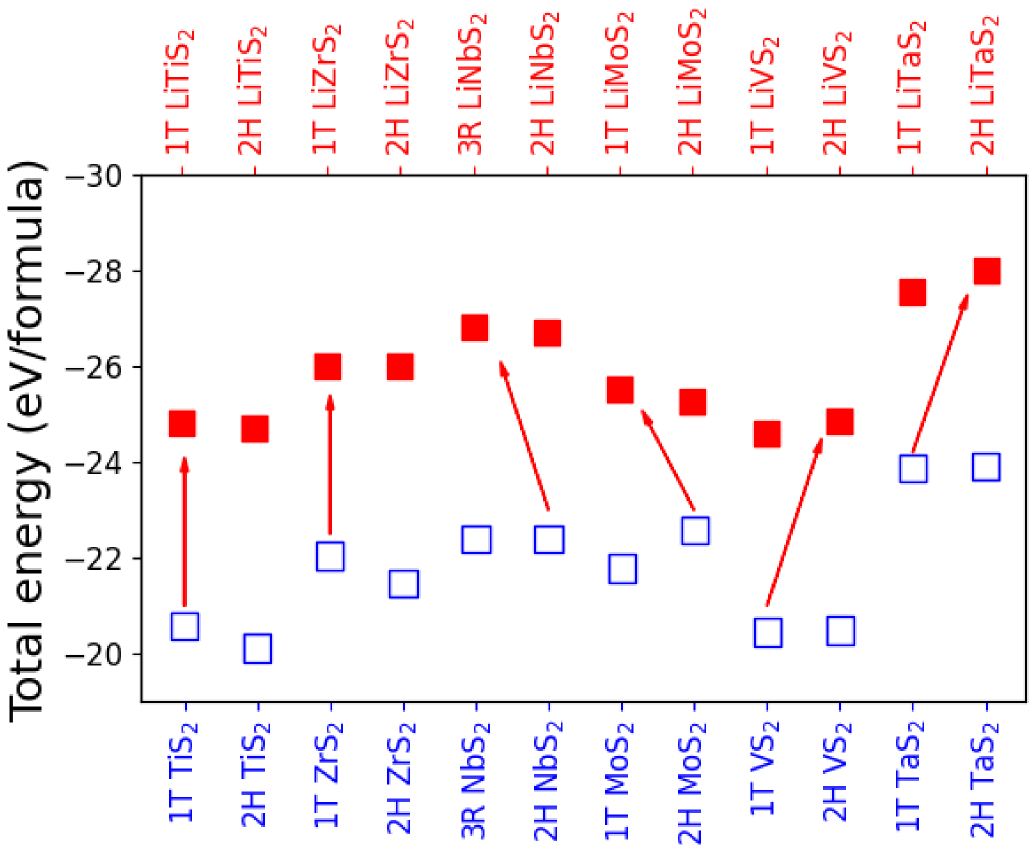

Fig. S8 | Total energy of TMDs and lithium intercalated TMDs. A chemical with lower total energy is thermodynamically favorable.

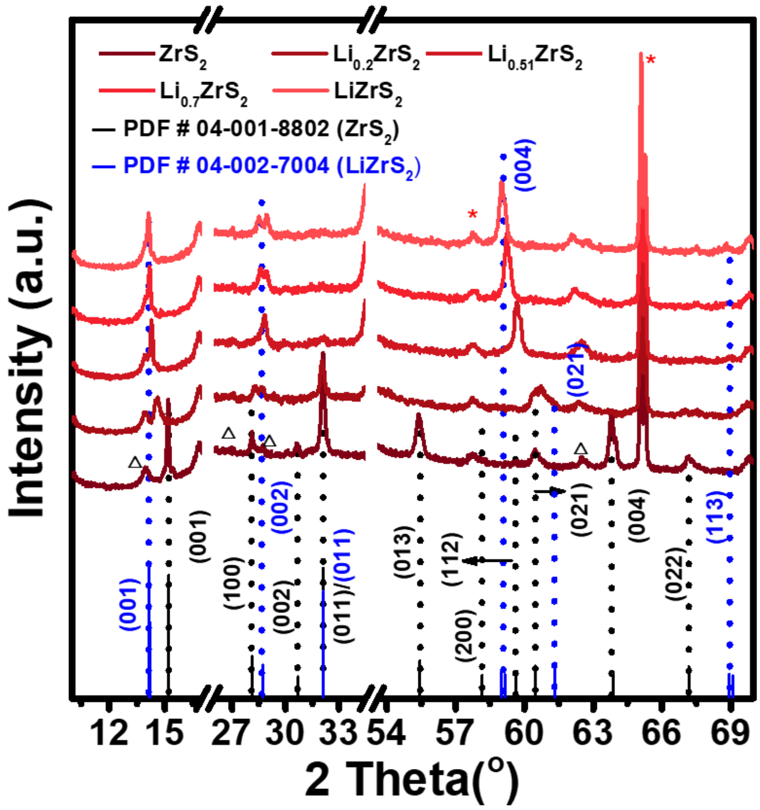

Fig. S9 | Selected XRD pattern of Li_xZrS_2 during in-situ test. During electrochemical lithium intercalation, 1T phase ZrS_2 changes to 1T $LiZrS_2$.

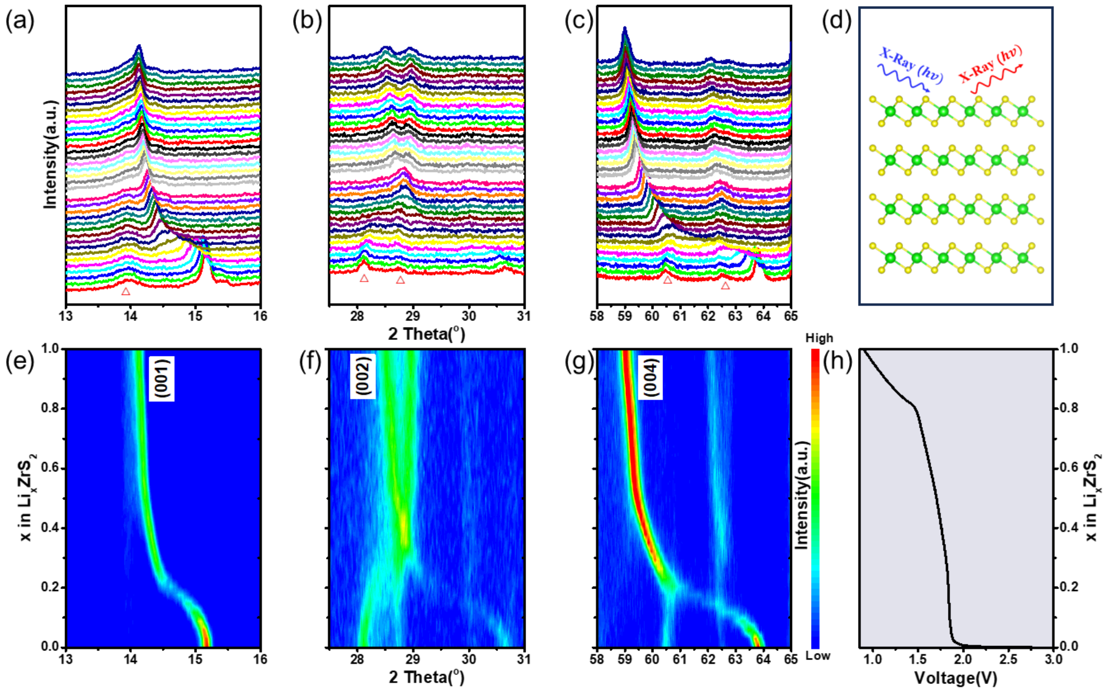

Fig. S10 | In situ XRD patterns of ZrS_2 during the electrochemical lithium intercalation. (a) Stacked XRD pattern of (*001*) diffraction peak. (b) Stacked XRD pattern of (*002*) diffraction peak. (c) Stacked XRD pattern of (*004*) diffraction peak. (d) Schematic of diffraction of X-rays in ZrS_2. (e)-(g) Countour plots corresponding to (a)-(c). The (*00l*) peaks shift to a lower angle, indicating the increase of the interlayer spacing. (h) Typical x-voltage profile.

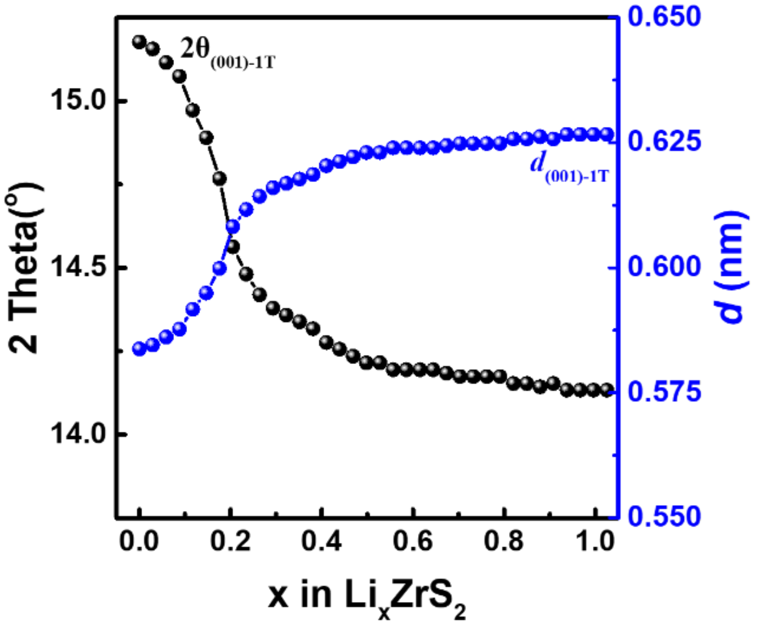

Fig. S11 | Lattice parameters evolution of Li_xZrS_2 during electrochemical lithium intercalation.

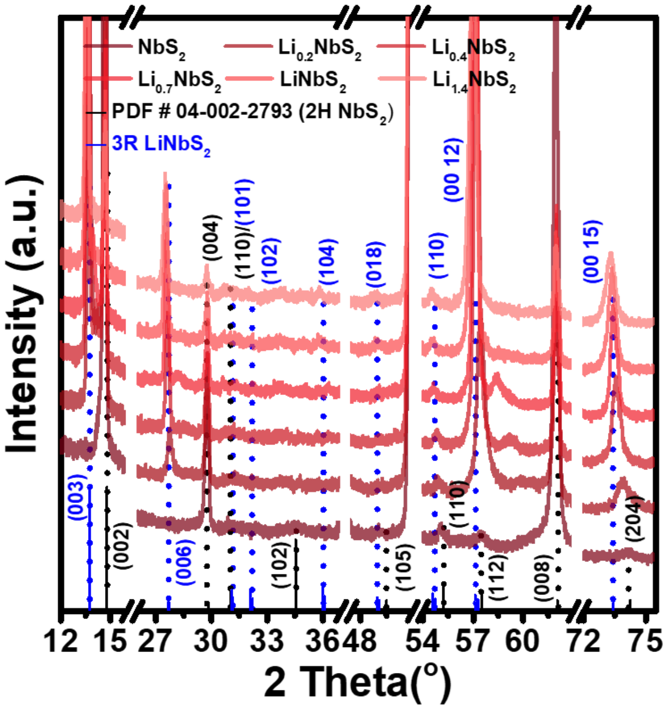

Fig. S12 | Selected XRD pattern of Li_xNbS_2 during in-situ test. During electrochemical lithium intercalation, 2H phase NbS_2 changes to 3R Li_xNbS_2. The 3R $LiNbS_2$ belongs to R3m space group with lattice parameters *a* = *b* = 0.3359 nm, and *c* = 1.9330 nm.

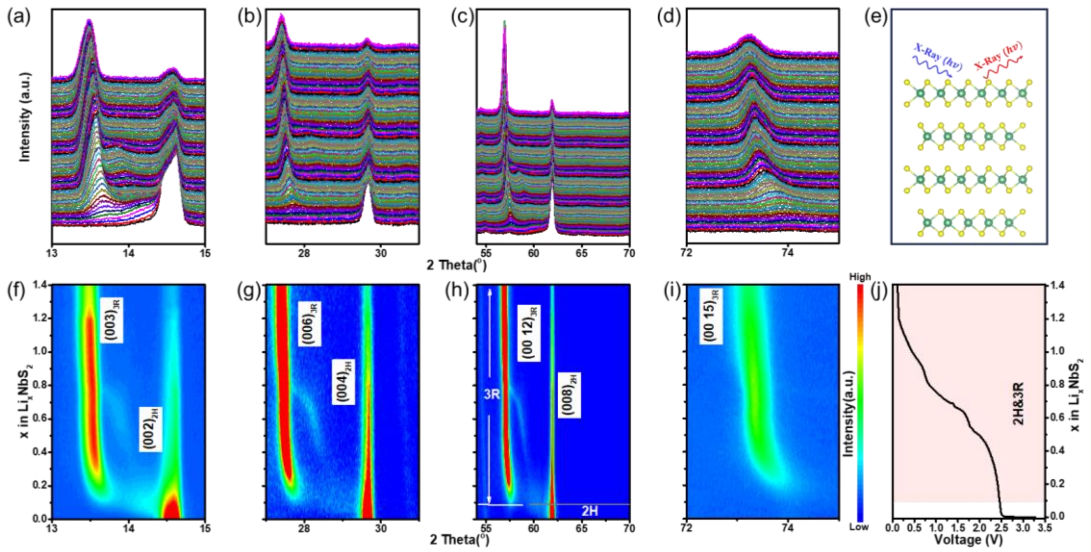

Fig. S13 | In situ XRD patterns of NbS_2 during the electrochemical lithium intercalation. (a) Stacked XRD pattern of $(002)_{2H}/(003)_{3R}$ diffraction peak. (b) Stacked XRD pattern of $(004)_{2H}/(006)_{3R}$ diffraction peak. (c) Stacked XRD pattern of $(008)_{2H}/(00\ 12)_{3R}$ diffraction peak. (d) Stacked XRD pattern of $(00\ 15)_{3R}$ diffraction peak. (e) Schematic of diffraction of X-rays in NbS_2. (f)-(i) Countour plots corresponding to (a)-(d). (j) Typical x-voltage profile.

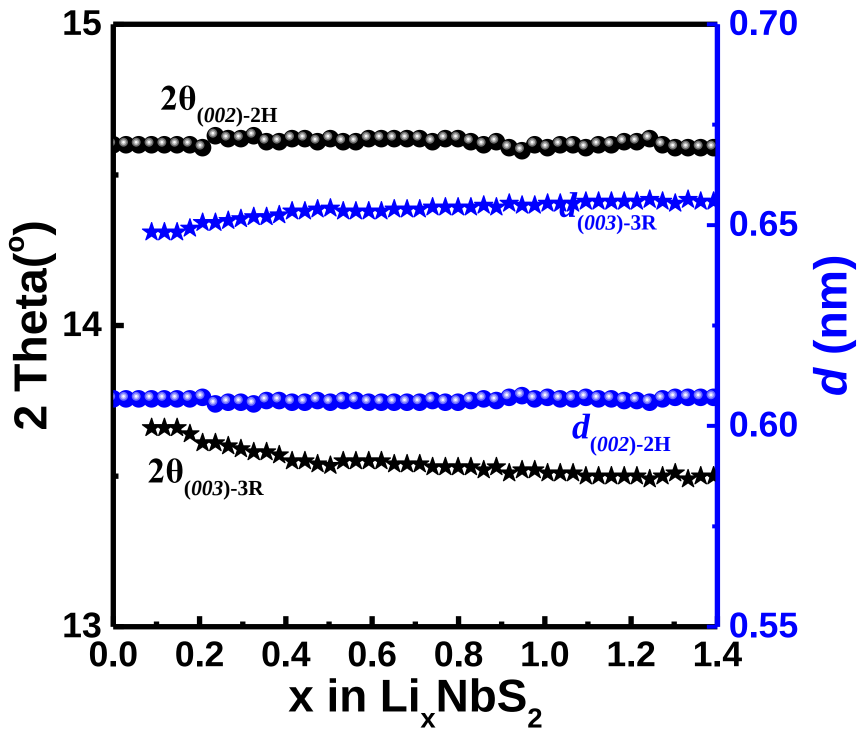

Fig. S14 | Lattice parameters evolution of Li$_x$NbS$_2$ during electrochemical lithium intercalation.

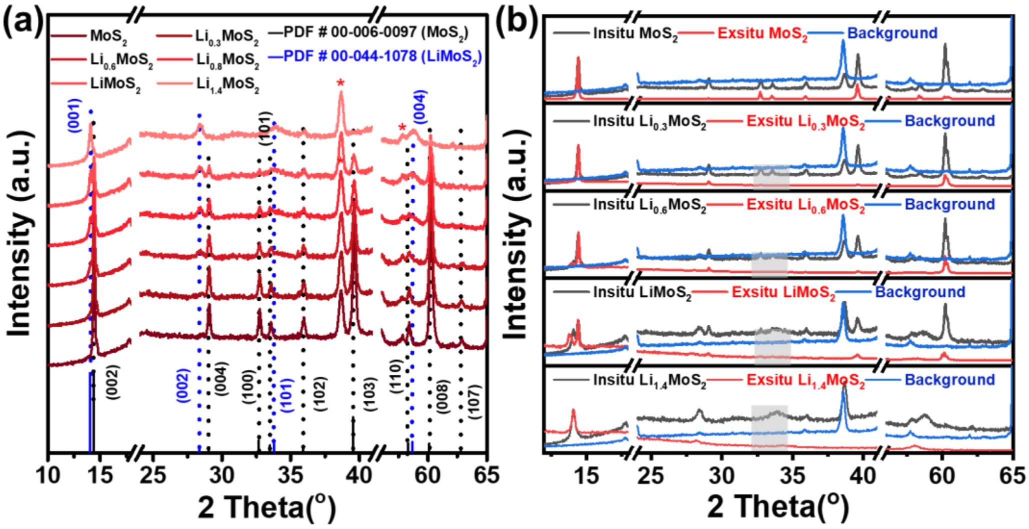

Fig. S15 | Selected XRD pattern of Li_xMoS_2 during in-situ and ex-situ test. (a) Selected in-situ XRD with index. (b) Comparison of in-situ and ex-situ XRD. During electrochemical lithium intercalation, 2H phase MoS_2 changes to 1T Li_xMoS_2.

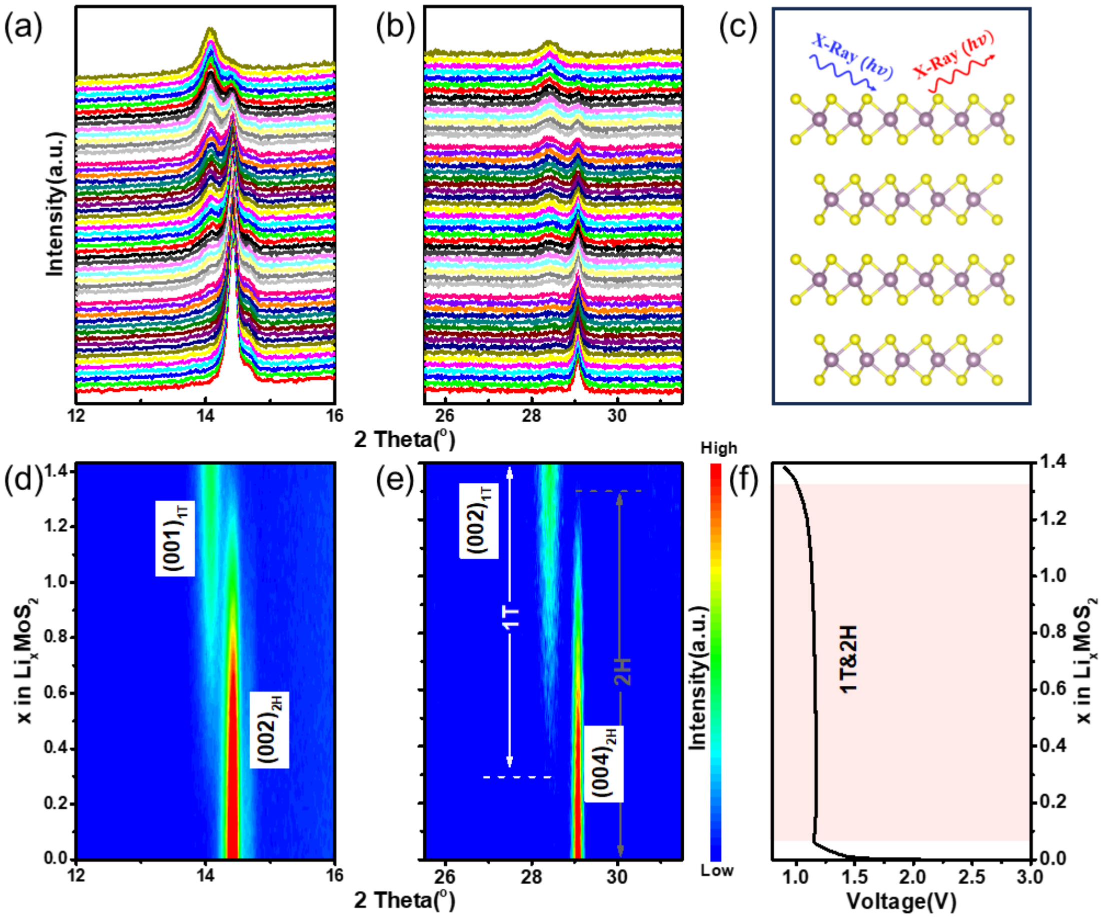

Fig. S16 | 2D in situ XRD patterns of MoS_2 during the electrochemical lithium intercalation. (a) Stacked XRD pattern of $(\mathit{002})_{2H}/(\mathit{001})_{1T}$ diffraction peak. (b) Stacked XRD pattern of $(\mathit{004})_{2H}/(\mathit{002})_{1T}$ diffraction peak. (c) Schematic of diffraction of X-rays in MoS_2. (d)-(e) Countour plots corresponding to (a)-(b). (f) Typical x-voltage profile. The 1T phase appears when around 0.3 mol lithium intercalates into 1mol MoS_2.

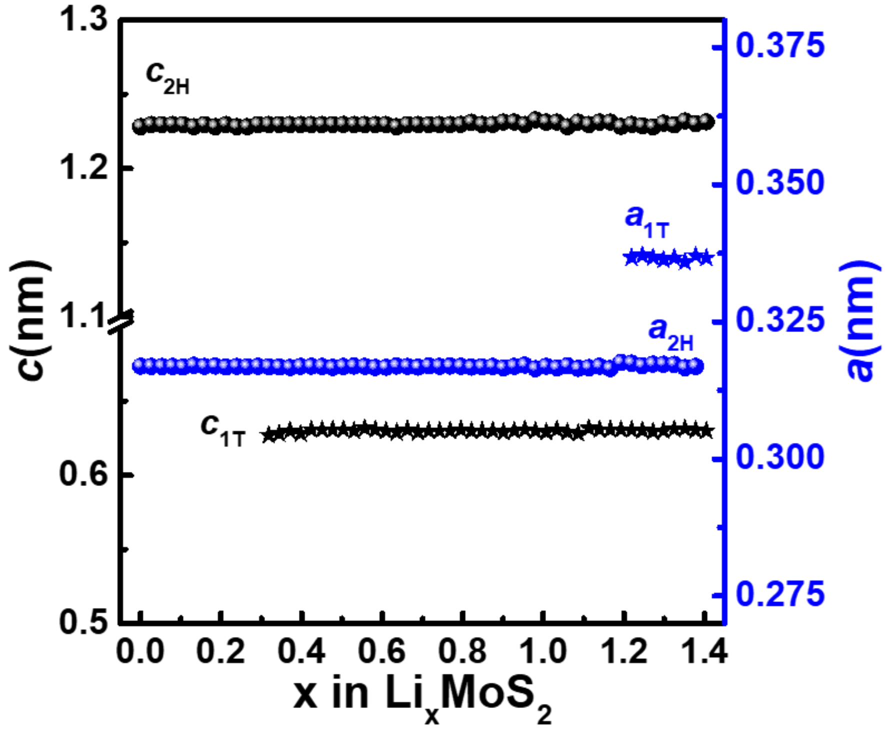

Fig. S17 | Lattice parameters evolution of Li_xMoS_2 during electrochemical lithium intercalation. Interlayer spacing of 2H MoS_2 is around 0.616 nm ($c_{2H}/2$) and of Li_xMoS_2 is around 0.630 nm (c_{1T}).

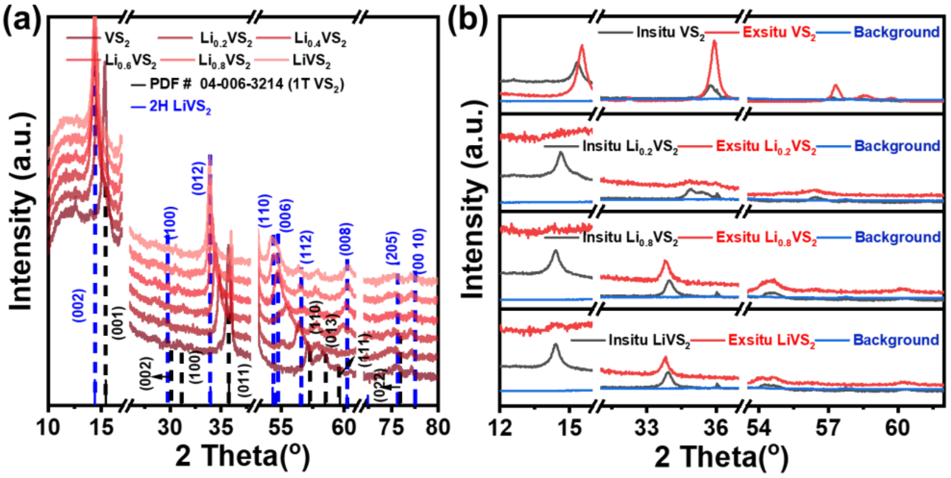

Fig. S18 | Selected XRD pattern of Li_xVS_2 during in-situ test and ex-situ test. (a) Selected in-situ XRD with index. (b) Comparison of in-situ and ex-situ XRD. During electrochemical lithium intercalation, 1T phase VS_2 changes to 2H $LiVS_2$. The 2H $LiVS_2$ belongs to P63/mmc space group with lattice parameters a =0.33741 nm and c = 1.22185 nm.

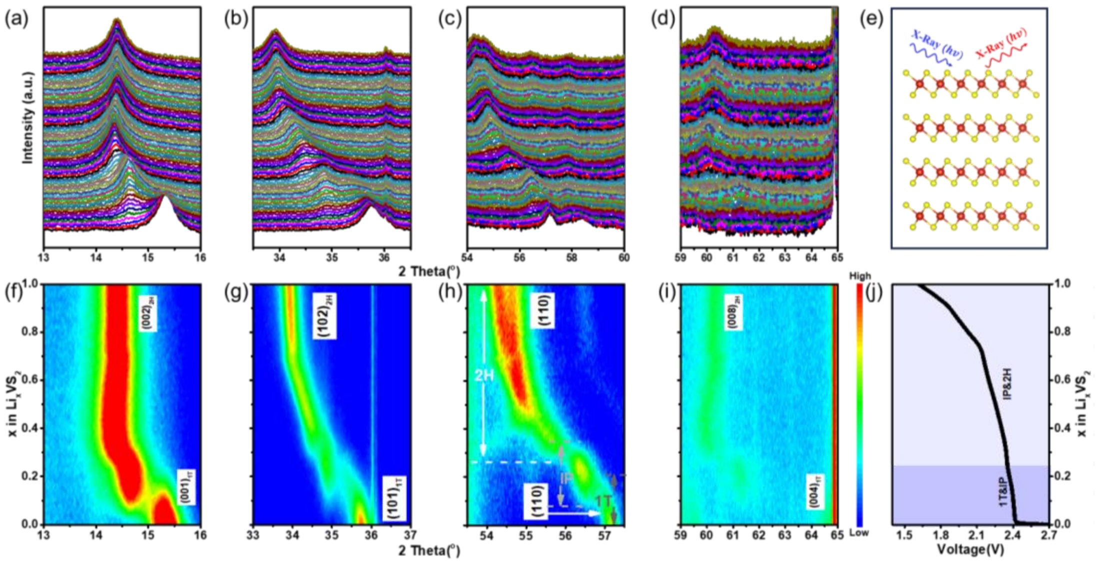

Fig. S19 | In situ XRD patterns of VS_2 during the electrochemical lithium intercalation. (a) Stacked XRD pattern of $(\mathit{002})_{2H}/(\mathit{001})_{1T}$ diffraction peak. (b) Stacked XRD pattern of $(\mathit{102})_{2H}/(\mathit{102})_{1T}$ diffraction peak. (c) Stacked XRD pattern of $(\mathit{110})_{2H}/(\mathit{110})_{1T}$ diffraction peak. (d) Stacked XRD pattern of $(\mathit{008})_{2H}/(\mathit{004})_{1T}$ diffraction peak. (e) Schematic of diffraction of X-rays in VS_2. (f)-(i) Countour plots corresponding to (a)-(d). (j) Typical x-voltage profile. An intermediate phase (IP) of Li_xVS_2 appears at the expense of VS_2 when x exceeds ~0.08, and then VS_2 disappears at x = ~0.27. As lithium intercalation proceeds, 2H Li_xVS_2 arises at x = ~0.28 and IP fades at x = ~0.37.

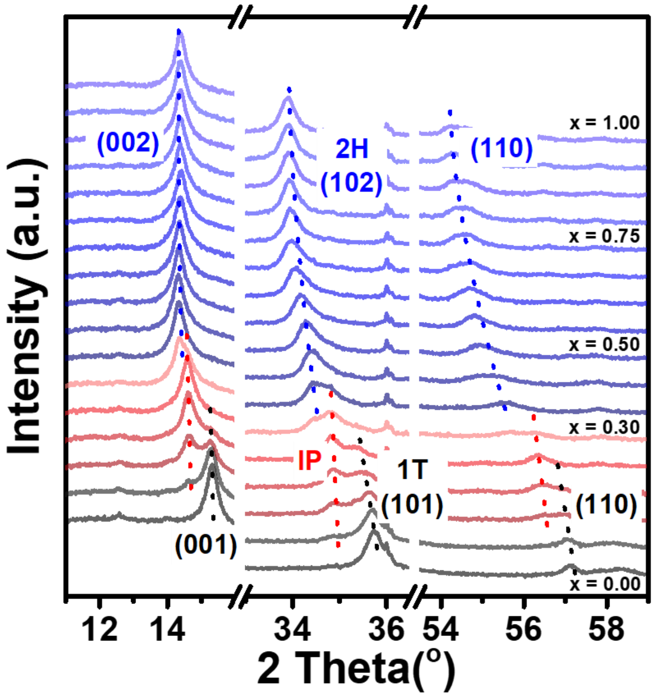

Fig. S20 | Evolution of (*001*)/(*002*), (*101*)/(*102*), and (*110*) reflections of the selected in-situ XRD patterns during the electrochemical lithium intercalation. 1T phase is the pristine phase(x = 0.0), IP is the intermediate phase, 2H $LiVS_2$ is the fully intercalated phase(x = 1.0).

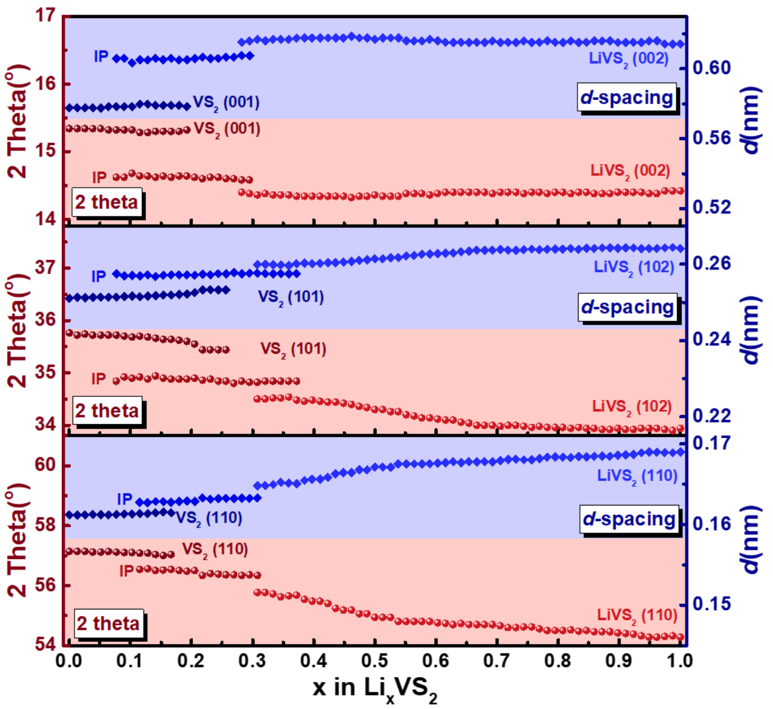

Fig. S21 | Interlayer spacing evolution of Li_xVS_2 during electrochemical lithium intercalation. The interlayer spacing of (*002*) in $LiVS_2$ is larger than the (*001*) interlayer spacing of VS_2, and it changes slightly, which means the *c* lattice does not change so much. The increase of interlayer spacing of (*102*) and (*110*) in $LiVS_2$ is attributed to the increase of *a* lattice.

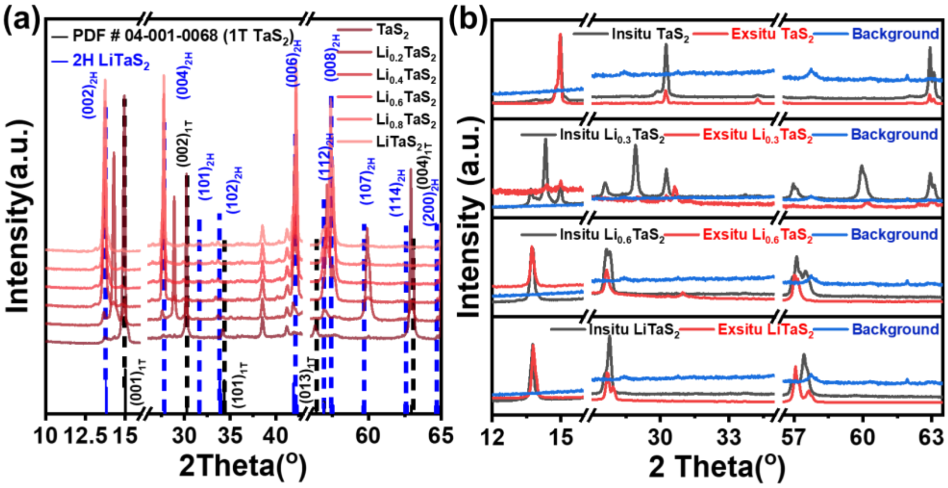

Fig. S22 | Selected XRD pattern of Li_xTaS_2 during in-situ test and ex-situ test. (a) Selected in-situ XRD with index. (b) Comparison of in-situ and ex-situ XRD. During electrochemical lithium intercalation, 1T phase TaS_2 changes to 2H $LiTaS_2$. The 2H $LiTaS_2$ belongs to $P6_3/mmc$ space group with lattice parameters a =0. 335046 nm and c = 1.28306 nm.

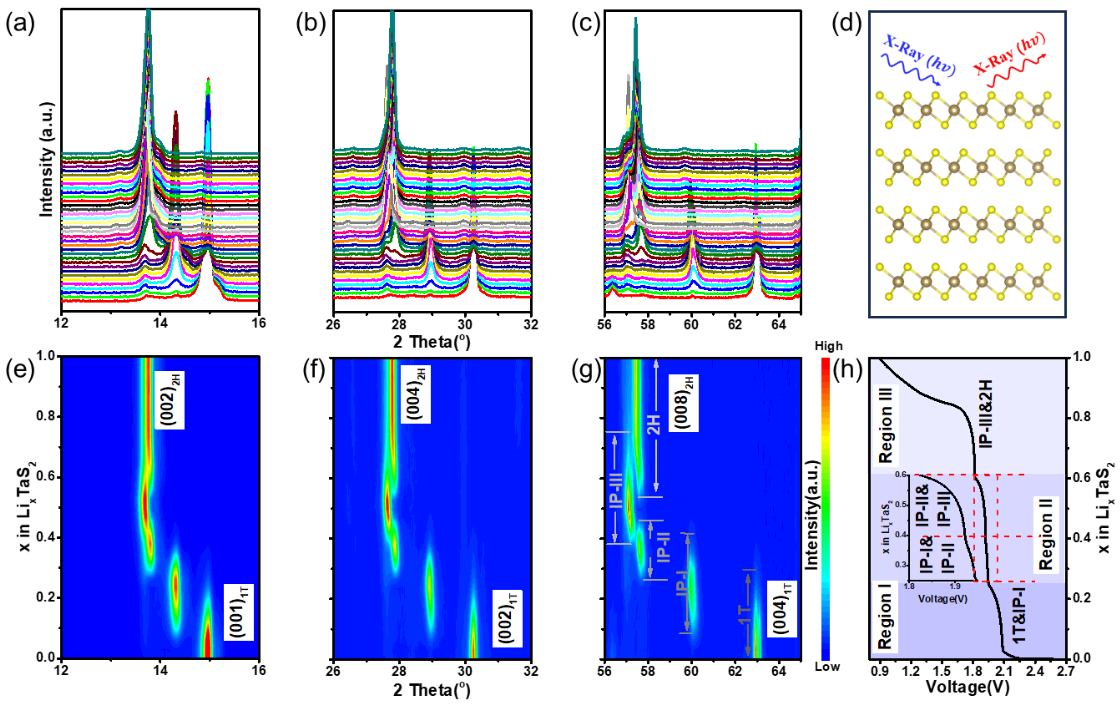

Fig. S23 | Evolution of (*00l*)(*l* = 1,2,4,8) reflections corresponding to the typical discharge profile. (a) Stacked XRD pattern of $(001)_{1T}/(002)_{2H}$ diffraction peak. (b) Stacked XRD pattern of $(002)_{1T}/(004)_{2H}$ diffraction peak. (c) Stacked XRD pattern of $(004)_{1T}/(008)_{2H}$ diffraction peak. (d) Schematic of diffraction of X-rays in TaS_2. (e)-(g) Countour plots corresponding to (a)-(c). (h) Typical x-voltage profile. 1T phase is the pristine phase(x = 0.0), IP-I~IP-III is the intermediate phase, 2H $LiTaS_2$ is the fully intercalated phase(x = 1.0). During lithiation, the diffraction intensity of the initial phase of bulk 1T TaS_2 decreases, and then an IP-I appears at the expense of 1T TaS_2 when x = ~0.06. The 1T phase disappears at x ~= 0.35. Therefore, region I involves a two-phase reaction between 1T TaS_2 and the IP-I. IP-I exists in the range of $0.05 \le x \le 0.41$. A new IP-II emerges at x ~= 0.26 and disappears at x ~= 0.44. Region II is the coexistence of IP-I and IP-II. The IP-III appears at x ~= 0.41 and dominates the sub-stage in region II, then disappears at x ~= 0.76. Finally, the IP-III and 2H Li_xTaS_2 hold region III. 2H Li_xTaS_2 exists at the range of $0.53 \le x \le 1.0$.

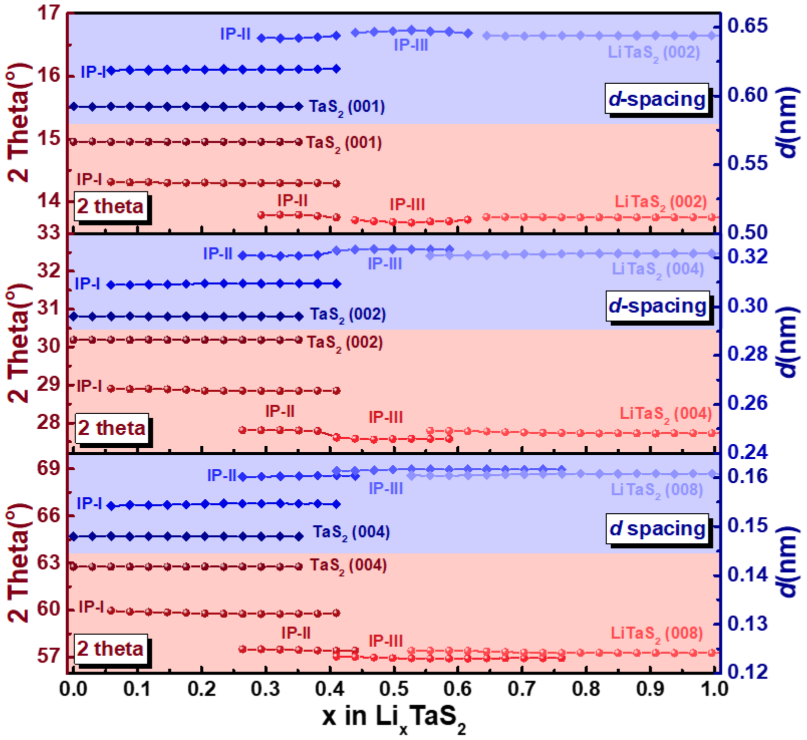

Fig. S24 | Interlayer spacing evolution of Li_xTaS_2 during electrochemical lithium intercalation. Lithium intercalation expands the interlayer spacing of 1T TaS_2. The interlayer spacings are in the order of TaS_2 < IP-I < IP-II < $LiTaS_2$ < IP-III. For each phase (intermediate phase), the interlayer spacing changes slightly or does not change.

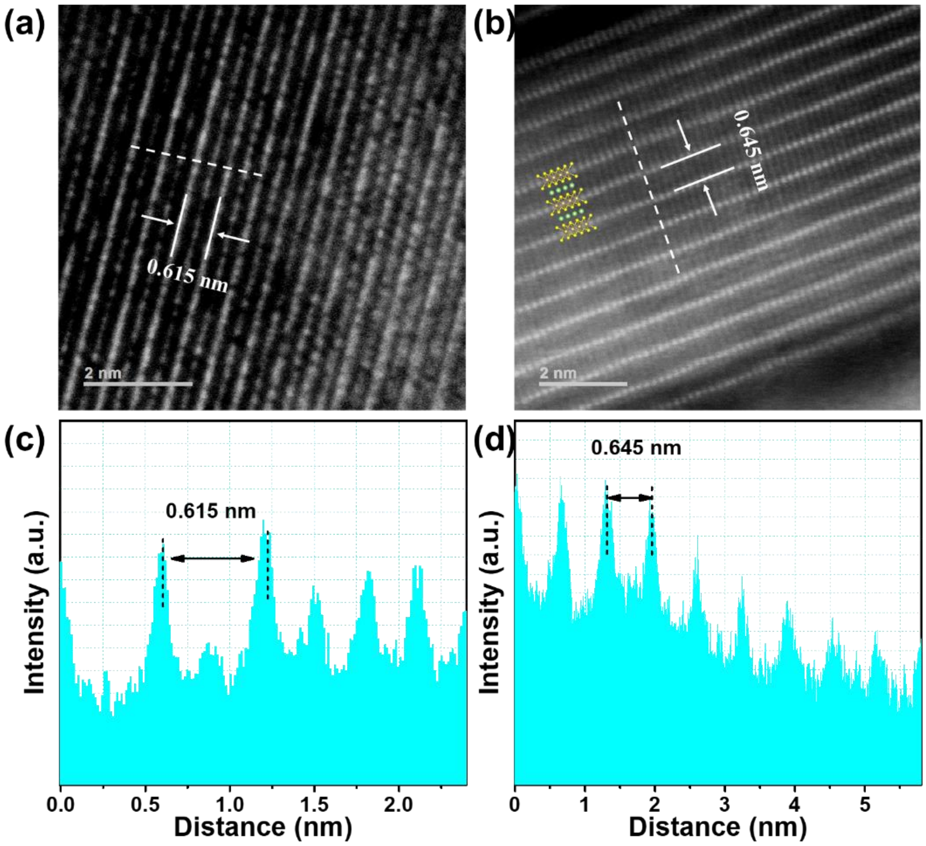

Fig. S25 | Structural characterization of $Li_{0.3}TaS_2$ and $LiTaS_2$. (a), (b) STEM image of $Li_{0.3}TaS_2$ and $LiTaS_2$, respectively. (c), (d) Atom intensity profiles along the dashed line in (a) and (b).

Table S1 | Lattice parameters of TMDs with and without Li intercalation obtained from DFT calculations.

		MS_2		$LiMS_2$	
		a (Å)	*c* (Å)	*a* (Å)	*c* (Å)
M = Ti	1T	3.397	5.724	3.398	6.098
	2H	3.335	11.995	3.378	12.440
M = Zr	1T	3.660	5.854	3.539	6.295
	2H	3.561	12.346	3.538	12.612
M = Nb	3R	3.318	17.984	3.335	19.049
	2H	3.320	12.169	3.337	12.761
M = Mo	1T	3.181	6.010	3.266	6.356
	2H	3.162	12.332	3.165	13.671
M = V	1T	3.158	5.843	3.288	6.068
	2H	3.161	12.047	3.221	12.480
M = Ta	1T	3.350	5.936	3.347	6.343
	2H	3.322	12.309	3.327	12.806

Table S2 | Bond lengths of TMDs with and without Li intercalation obtained from DFT calculations.

		MS_2	$LiMS_2$	
		$d_{Mo\text{-}S}$ (Å)	$d_{Mo\text{-}S}$ (Å)	$d_{Li\text{-}S}$ (Å)
M = Mo	1T	2.42	2.45	2.48
	2H	2.40	2.46	2.55
M = Nb	3R	2.47	2.49	2.47
	2H	2.47	2.49	2.51
M = V	1T	2.34	2.38	2.48
	2H	2.35	2.37	2.49
M = Ta	1T	2.47	2.50	2.50
	2H	2.47	2.49	2.49
M = Ti	1T	2.42	2.45	2.52
	2H	2.44	2.47	2.52
M = Zr	1T	2.57	2.59	2.56
	2H	2.59	2.60	2.56

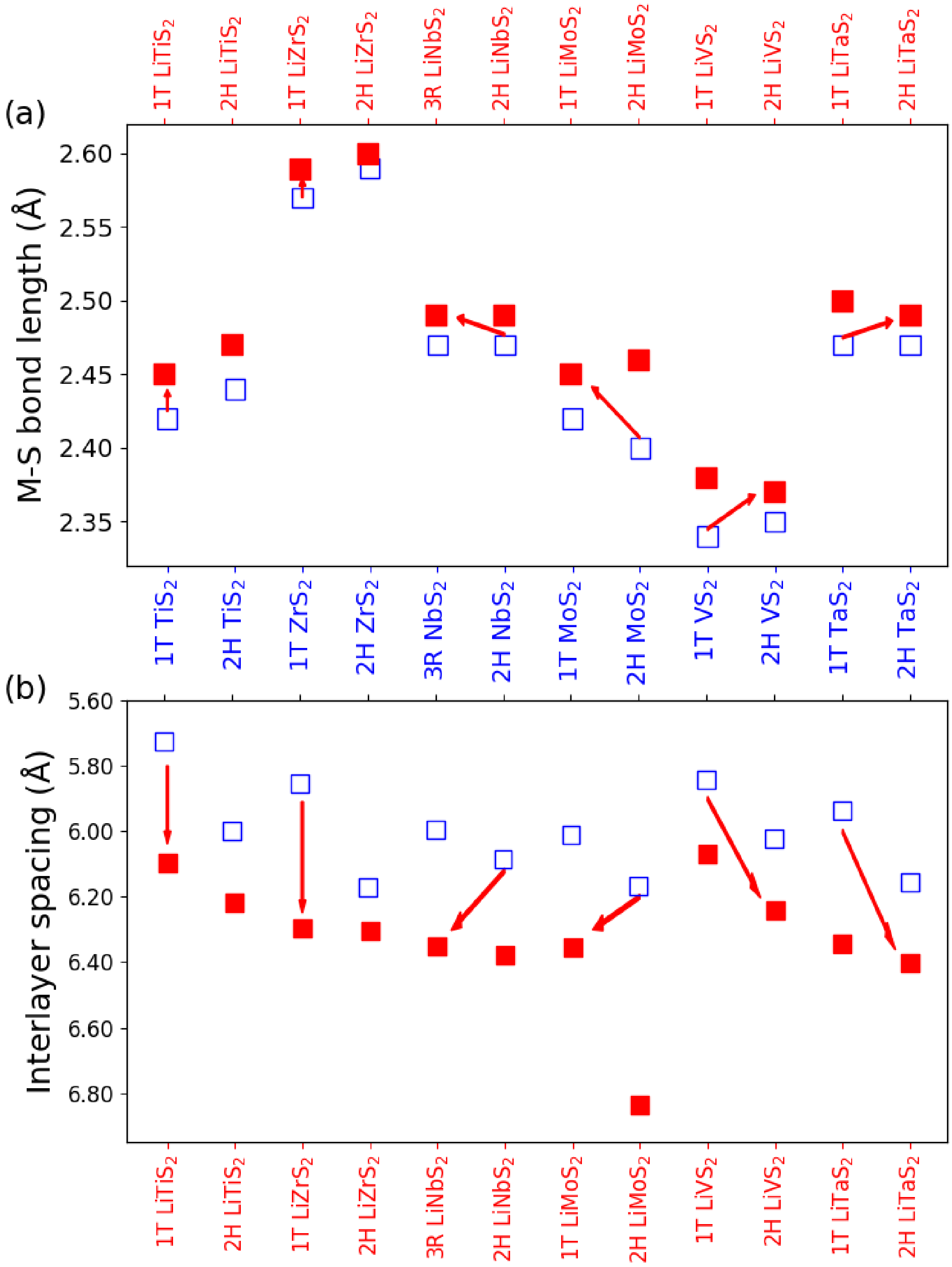

Fig. S26 | Structure change before and after electrochemical lithium intercalation. (a) Metal-Sulfur bond length. (b) Interlayer spacing. TMDs tend to transfer to the phase with a shorter M-S bond length. If the two phases have the same metal-sulfur bond length,(such as $LiNbS_2$), it tends to form a phase with smaller interlayer spacing.

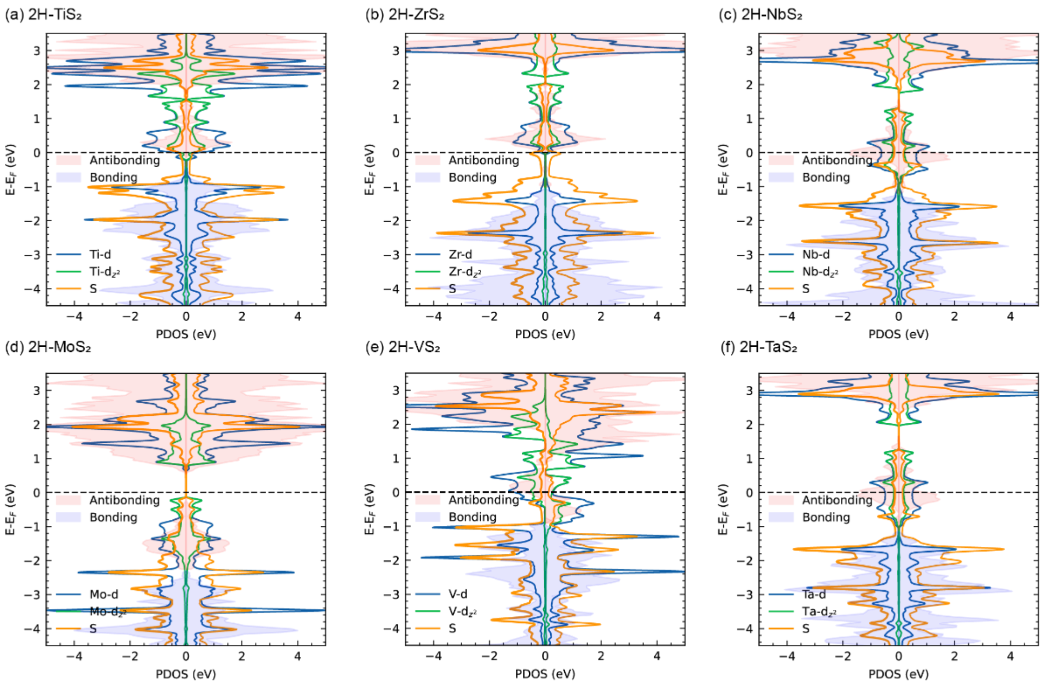

Fig. S27 | The projected density of state (PDOS) of atoms and the crystal orbital Hamilton population (–COHP) of Metal-S bonds in 2H MS_2. (a) 2H-TiS_2,(b) 2H-ZrS_2, (c) 2H-NbS_2, (d) 2H-MoS_2, (e) 2H-VS_2 and (f) 2H-TaS_2. Note that the positive and negative values of PDOS and -COHP were attributed to the contribution of spin-up and spin-down. The blue and red filled area represents the metal-S bonding and antibonding interaction based on -COHP analysis.

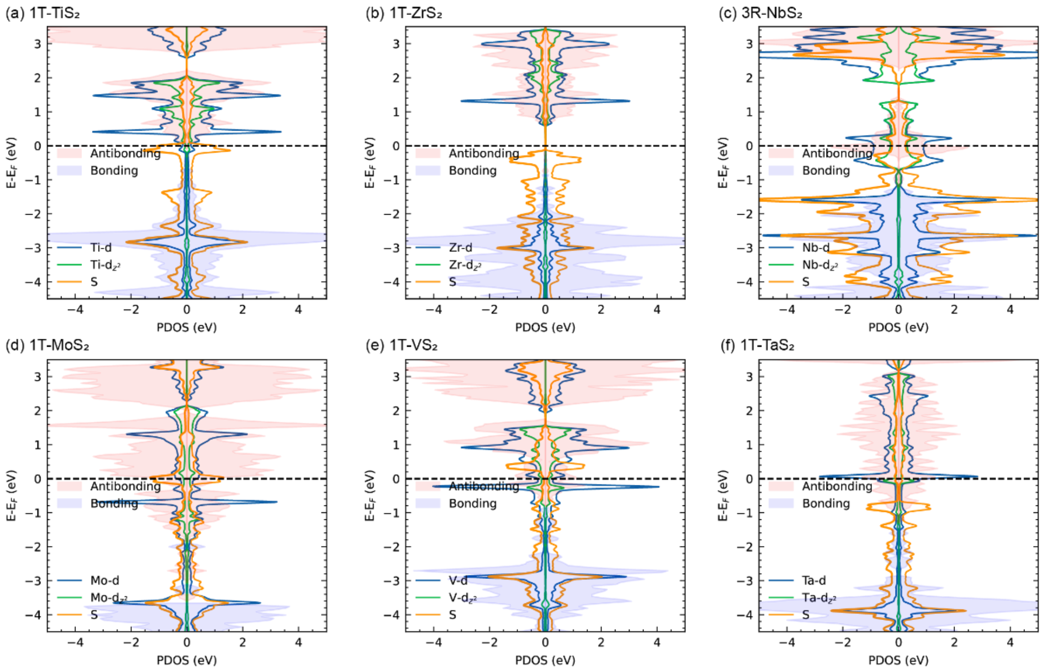

Fig. S28 | The projected density of state (PDOS) of atoms and the crystal orbital Hamilton population (–COHP) of Metal-S bonds in 1T/3R MS_2. (a) 1T-TiS_2,(b) 1T-ZrS_2, (c) 3R-NbS_2, (d) 1T-MoS_2, (e) 1T-VS_2 and (f) 1T-TaS_2. Note that the positive and negative values of PDOS and -COHP were attributed to the contribution of spin-up and spin-down. The blue and red filled area represents the metal-S bonding and antibonding interaction based on -COHP analysis.

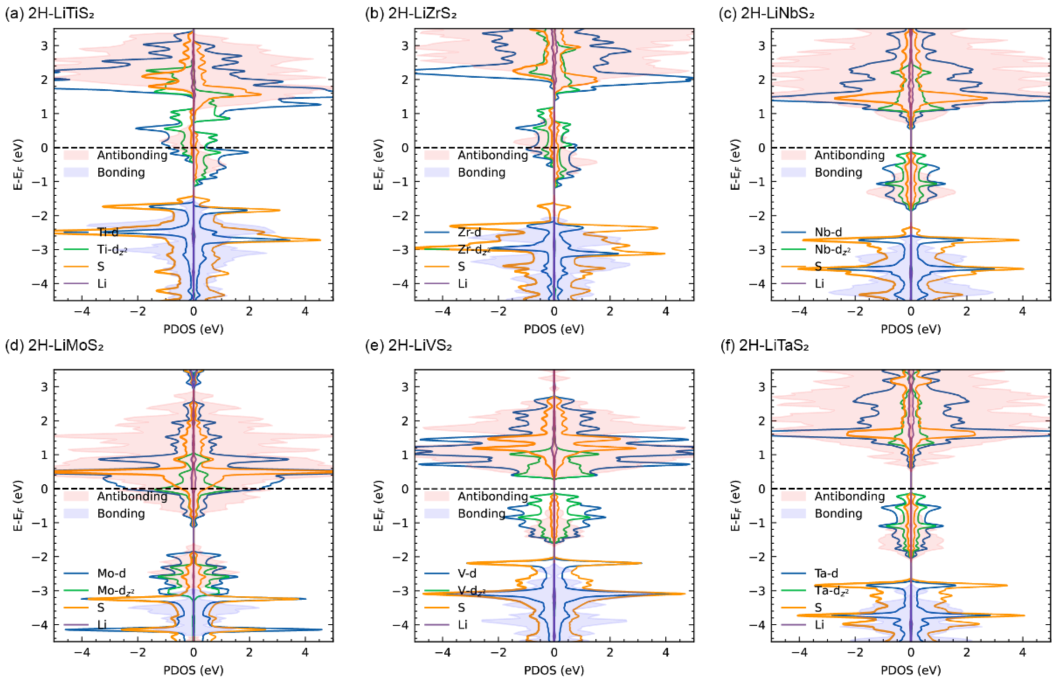

Fig. S29 | The projected density of state (PDOS) of atoms and the crystal orbital Hamilton population (–COHP) of Metal-S bonds in 2H $LiMS_2$. (a) 2H-$LiTiS_2$,(b) 2H-$LiZrS_2$, (c) 2H-$LiNbS_2$, (d) 2H-$LiMoS_2$, (e) 2H-$LiVS_2$ and (f) 2H-$LiTaS_2$. Note that the positive and negative values of PDOS and -COHP were attributed to the contribution of spin-up and spin-down. The blue and red filled area represents the metal-S bonding and antibonding interaction based on -COHP analysis.

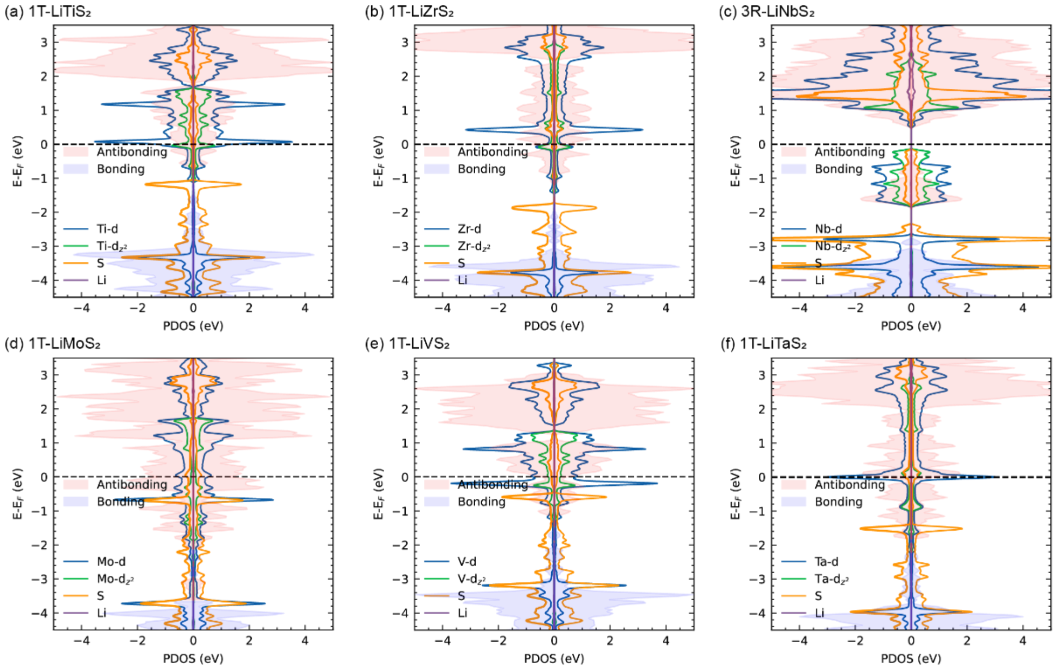

Fig. S30 | The projected density of state (PDOS) of atoms and the crystal orbital Hamilton population (–COHP) of Metal-S bonds in 1T/3R $LiMS_2$. (a) 1T-$LiTiS_2$,(b) 1T-$LiZrS_2$, (c) 3R-$LiNbS_2$, (d) 1T-$LiMoS_2$, (e) 1T-$LiVS_2$ and (f) 1T-$LiTaS_2$. Note that the positive and negative values of PDOS and -COHP were attributed to the contribution of spin-up and spin-down. The blue and red filled area represents the metal-S bonding and antibonding interaction based on -COHP analysis.

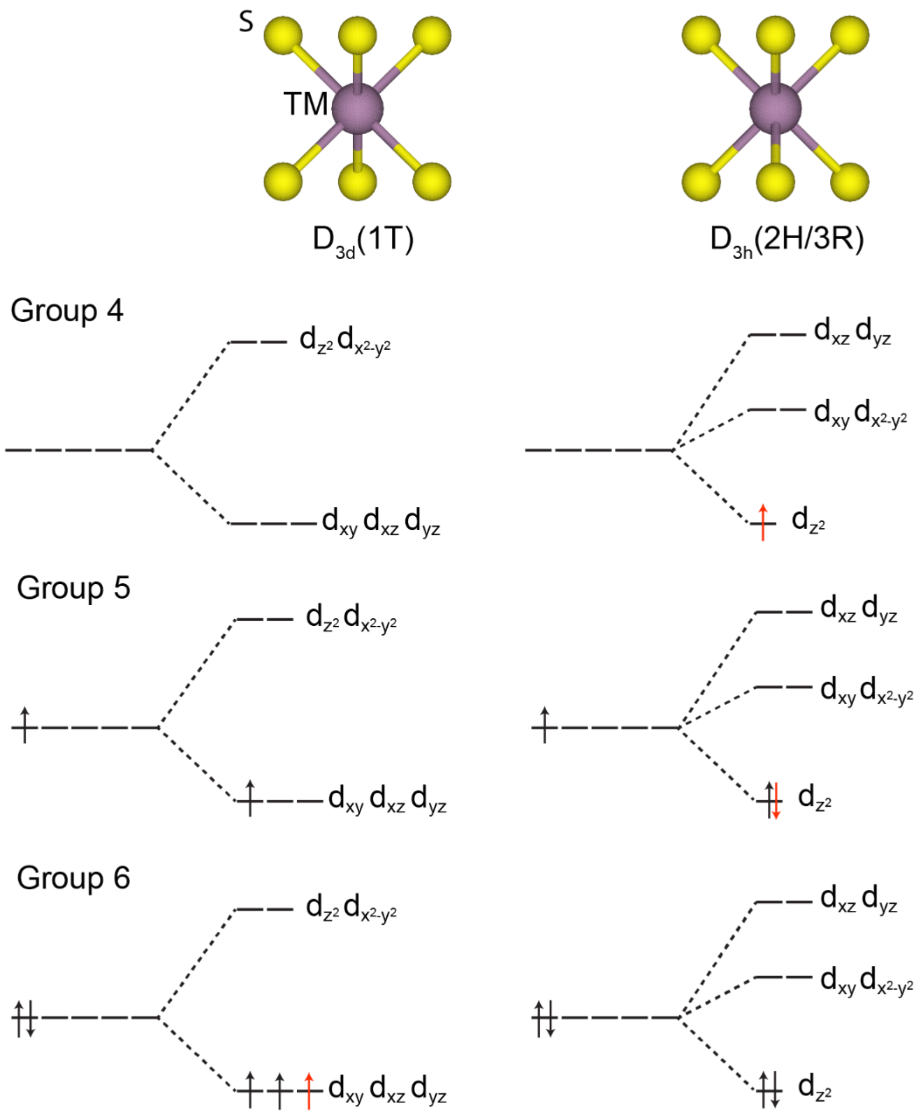

Fig. S31 | The d-orbitals for trigonal prismatic coordination (D3h symmetry) and octahedrally coordinated (D3d symmetry) in d-metal dichalcogenides. Note that the red arrow denoted the electron donated from the Li atom upon the lithium intercalation. In the 1T phase of TMDs, the transition metal centers are octahedrally coordinated (D_{3d} symmetry) and exhibit degenerated d_{z^2,x^2-y^2} (e_g) and $d_{yz,xz,xy}$ (t_{2g}) orbitals. On the other hand, in the 2H phase, the transition metal centers adopt a trigonal prismatic coordination (D_{3h} symmetry) and form three groups of orbitals: d_{z^2} (a_1), $d_{x^2-y^2,xy}$ (e), and $d_{xz,yz}$(e′). These orbital configurations play a crucial role in understanding the electronic structure variations.

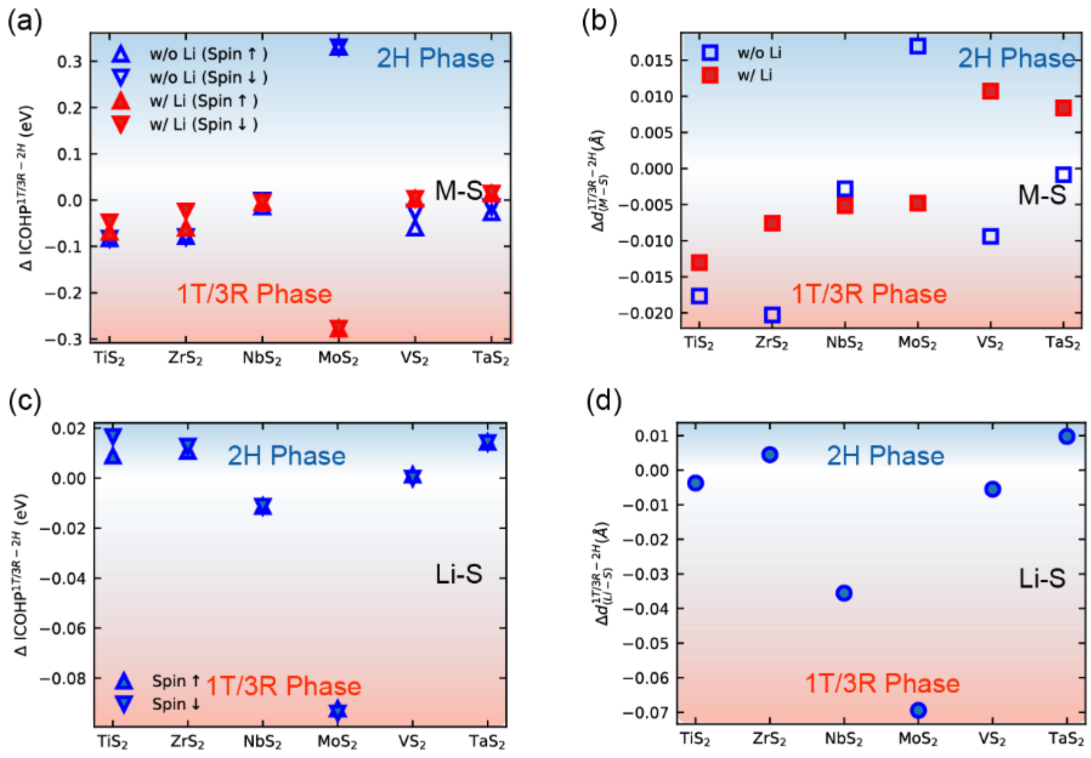

Fig. S32 | The effect of lithium intercalation on the metal-S and Li-S bond strength in TMDs. (a) The bond strength difference of the integrated COHP of Metal-S bonds between MS_2 and $LiMS_2$ (b) The difference of the bond length of Metal-S bonds between MS_2 and $LiMS_2$. (c) The bond strength difference of the integrated COHP of Li-S bonds in $LiMS_2$ (d) The difference of the bond length of Li-S bonds in $LiMS_2$. Note that $\Delta ICOHP^{1T/3R-2H} = ICOHP^{1T/3R} - ICOHP^{2H}$ and $\Delta d^{1T/3R-2H} = d^{1T/3R} - d^{2H}$.

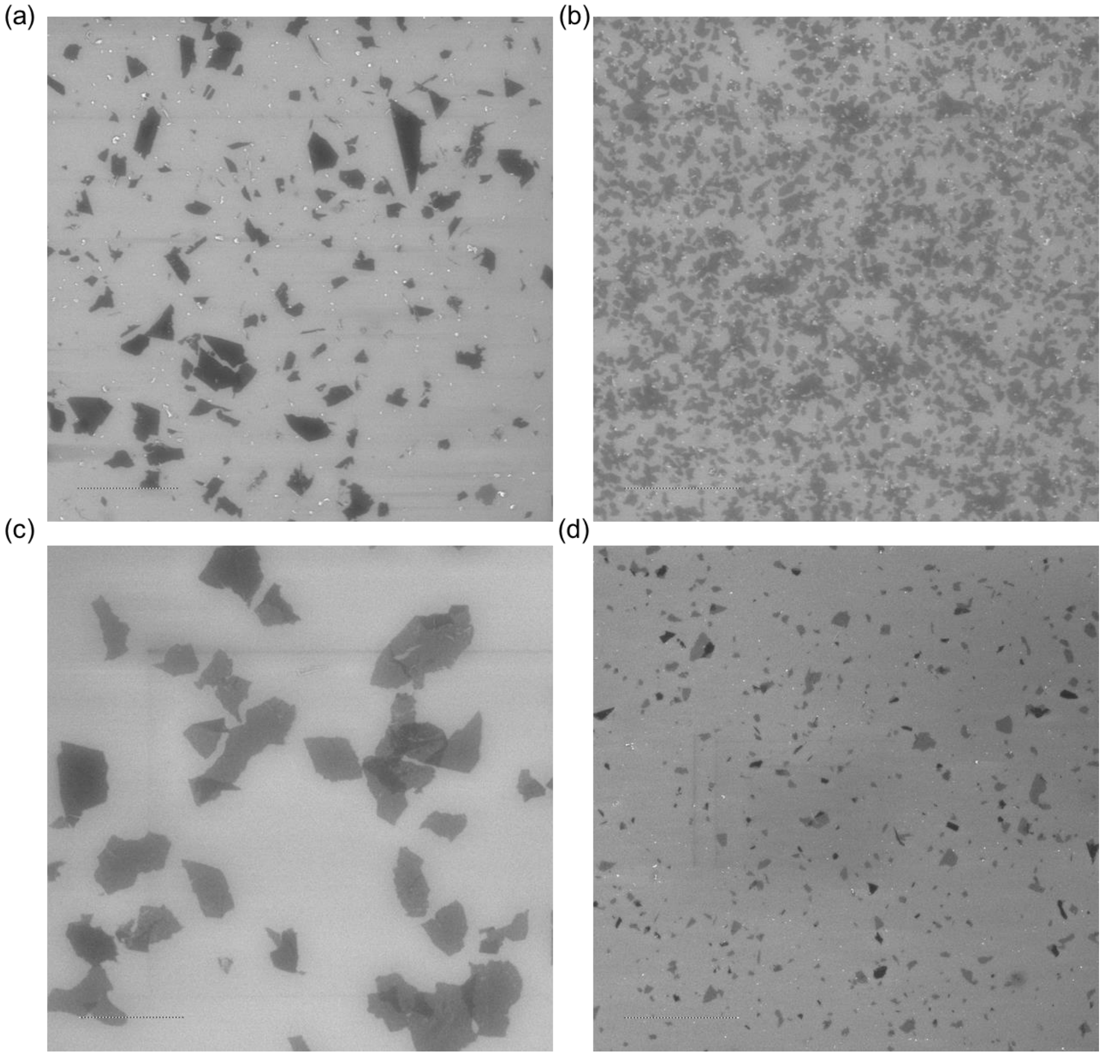

Fig. S33 | SEM images of exfoliated TMDs. (a) TiS_2, (b) ZrS_2, (c) MoS_2 and (d) TaS_2.

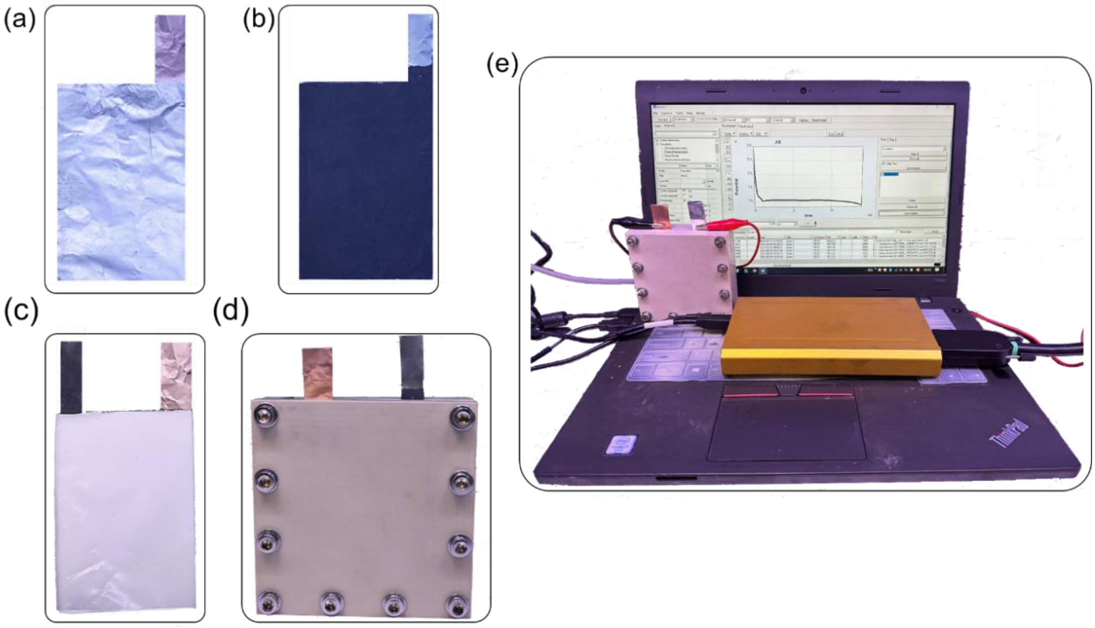

Fig. S34 | Scalable production of TMDs. (a) Lithium electrode with copper tab. (b) TMD electrode with Aluminum tab. (c) Pouch of lithium electrode and TMDs electrode. (d) The battery used to produce lithium intercalated TMDs. (e) Discharge of battery with TMDs electrode.

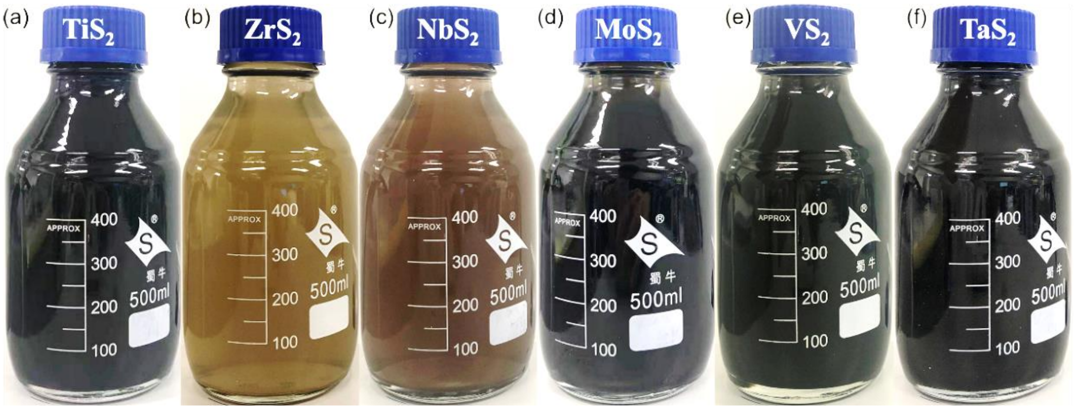

Fig. S35 | Dispersion of MS_2 nanosheets prepared by electrochemical lithium intercalation-assisted exfoliation of TMDs. (a) Dispersion of TiS_2. (b) Dispersion of ZrS_2. (c) Dispersion of NbS_2. (d) Dispersion of MoS_2. (e) Dispersion of VS_2. (f) Dispersion of TaS_2.

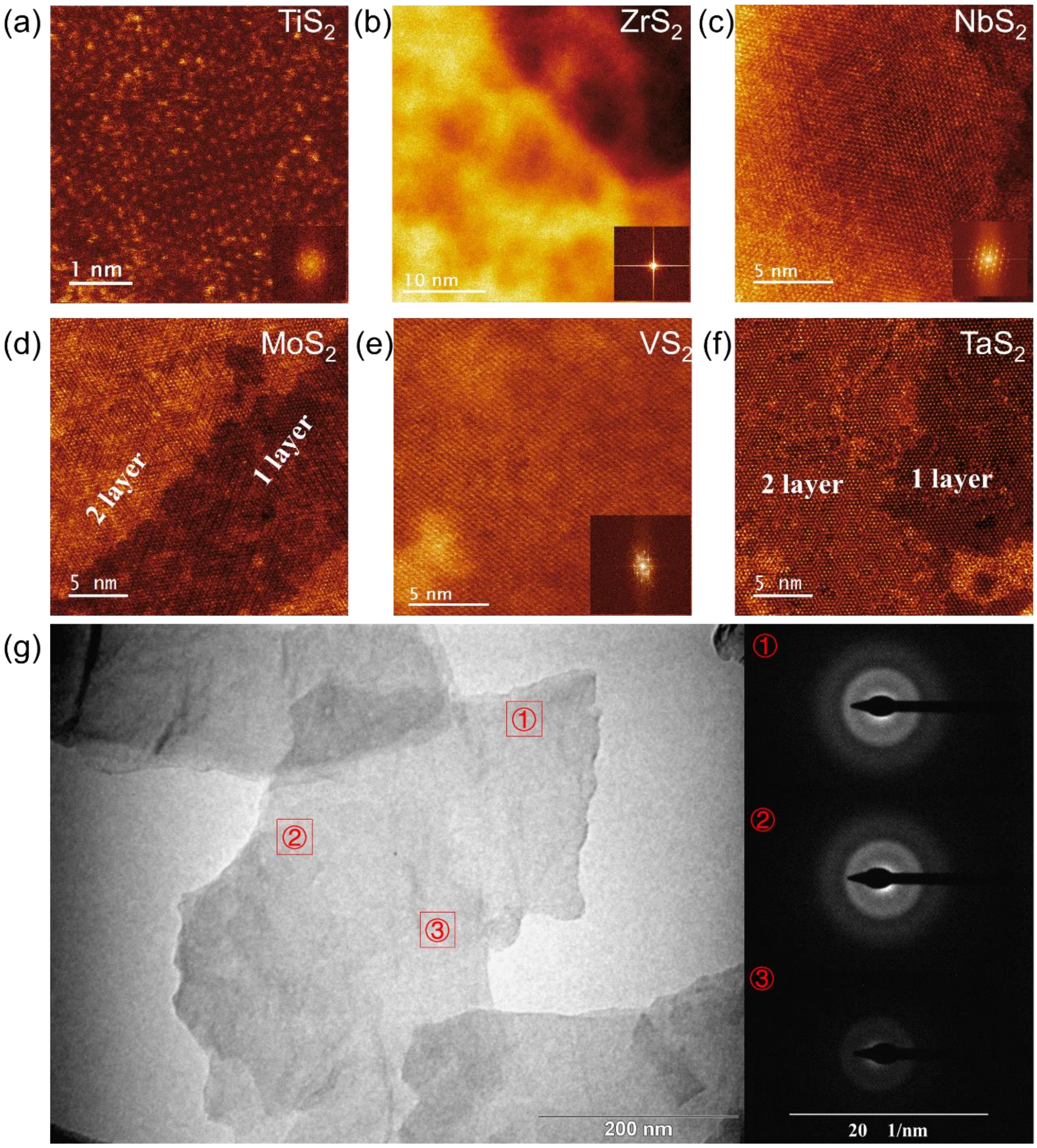

Fig. S36 | Structures of single/few-layer MS_2. (a) 1T phase TiS_2 along with corresponding FFT pattern, indicating a relatively poor crystallinity. (b) Amorphous ZrS_2 along with corresponding FFT pattern. (c) H phase NbS_2 with corresponding FFT pattern. (d) MoS_2 with one and double layers. (e) VS_2, insets are corresponding FFT patterns. (f) 1T TaS_2 with one and double layers. (g) ZrS_2, left panel: TEM image, right panel: SAED of the corresponding region in left panel.

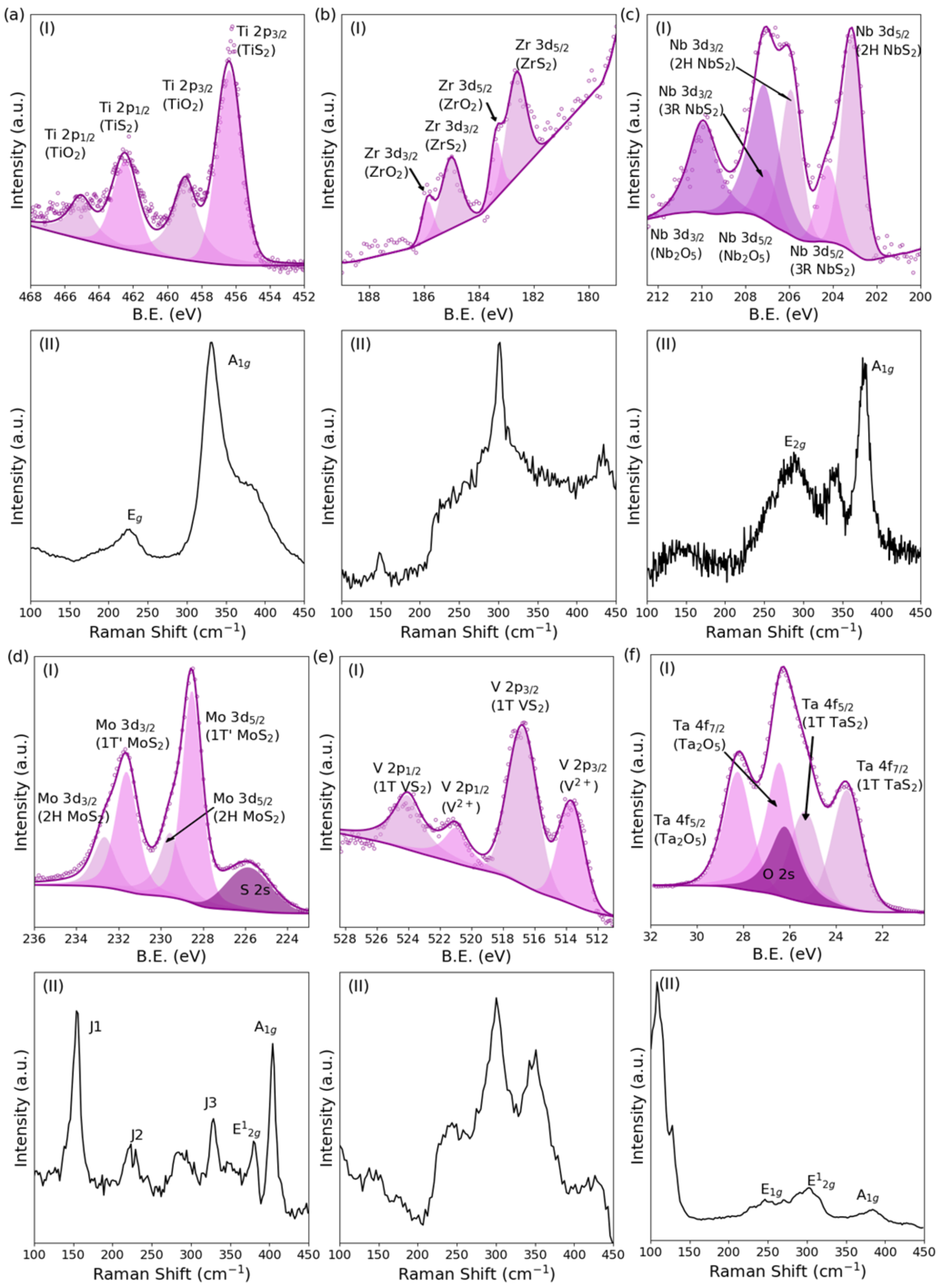

Fig. S37 | Raman and XPS of single/few-layer MS₂. (a) XPS of Ti 2p (I) with spectra of TiS_2 and TiO_2, and Raman of 1T TiS_2 with A_{1g} and E_g modes (II), (b) XPS of Zr 3d (II), and Raman of ZrS_2 (II). (c) XPS of Nb 3d with 2H, 3R doublets and doublet of Nb 3d from Nb_2O_5 (I), and Raman of single/few-layer NbS_2 with A_{1g} and E_{2g} modes of 2H phase and the peak between the A_{1g} and E_{2g} ascribed to the 3R (II). Given the identical structure of monolayer (H phase) in 2H and 3R phases, we deduced that the exfoliated NbS_2 holds the H phase. (d) XPS of Mo 3d with 2H, 1T' doublets (I) and Raman of MoS_2 with characteristics of 1T' and 2H phase (II). (e) XPS of V 2p with doublets from VS_2 and V^{2+} (I), and Raman of VS_2 (II). (f) XPS of Ta 4f with doublets from TaS_2 and Ta_2O_5 (I), and Raman of 1T TaS_2 (II).

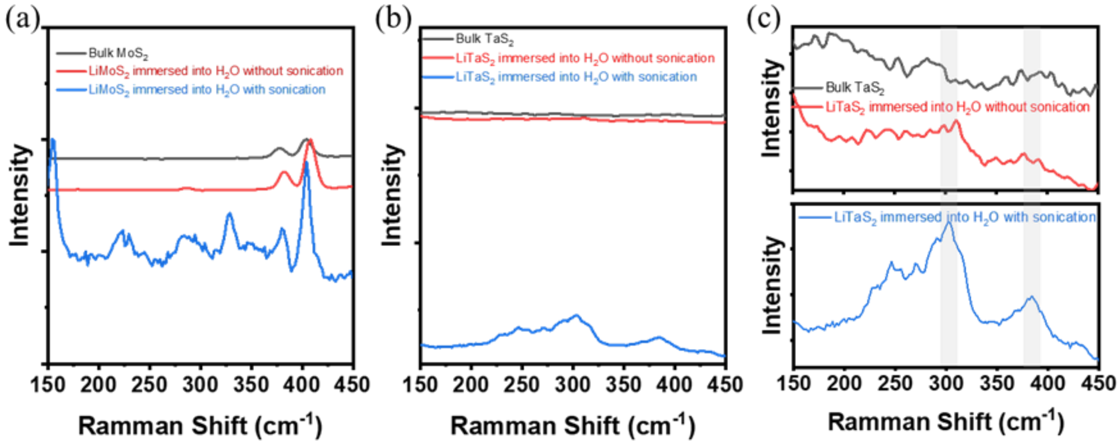

Fig. S38 | Raman of 2D MoS_2 and TaS_2 exfoliation from $LiMoS_2$ and $LiTaS_2$ in H_2O with or without ultrasonication. (a) MoS_2. (b) TaS_2. (c) Enlarged view of (b) .

Table S3 | Phase of MS_2 before electrochemical intercalation (Bulk MS_2), after intercalation ($LiMS_2$), after exfoliation (Exfoliated MS_2), and the potential applications of the atomically thin MS_2.

	TMDs	Bulk MS_2	$LiMS_2$	Exfoliated MS_2	Potential Applications
Phase Transition and Exfoliation	TiS_2	1T (semiconducting)	1T (semiconducting)	1T (semiconducting)	Sensors,[33] Batteries,[34] Thermoelectric generator,[35, 36]
	ZrS_2	1T (semiconducting)	1T (semiconducting)	Amorphous	Semiconductor,[37] Optoelectronics[37, 38]
	MoS_2	2H (semiconducting)	1T (metallic)	1T'&2H (semi-metallic& semiconducting)	Transistor,[39] OER,[40] Sensors,[41, 42] Batteries,[43] Semiconductor[44]
	NbS_2	2H (metallic)	3R (metallic)	H (metallic)	Batteries,[45] Sensors,[46] Superconductor[47]
	VS_2	1T	2H	1T	Supercapacitor,[48] HER,[49]

		(metallic)	(semiconducting)	(metallic)	Batteries[50]
	TaS_2	1T<br>(metallic)	2H<br>(metallic)	1T<br>(metallic)	HER,[51, 52] OER,[40]<br>Photocatalysis,[53]<br>Supercapacitor[54]

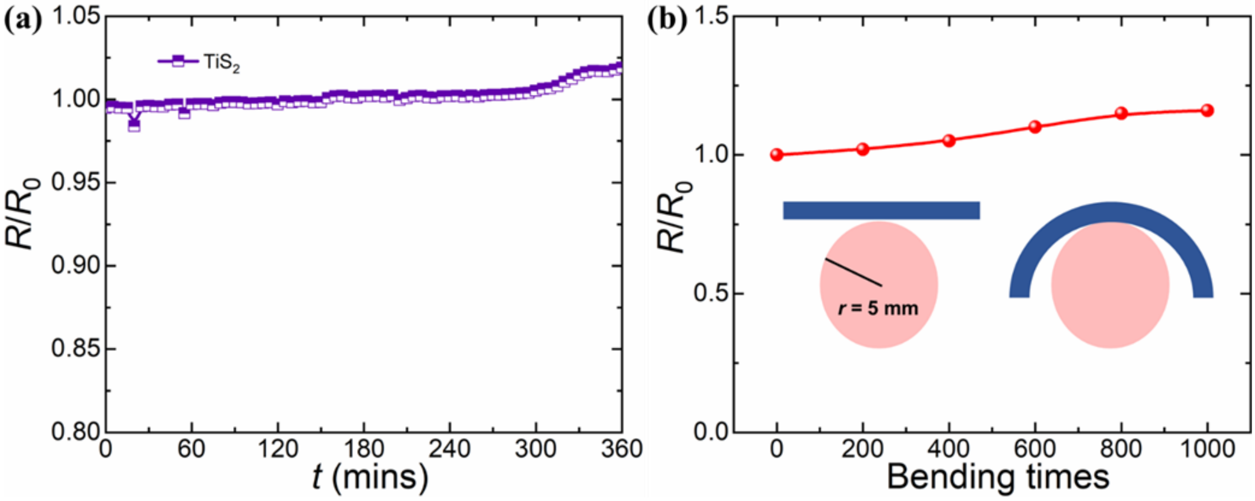

Fig. S39 | The time-dependent stability of TiS_2 films. (a) Resistance evolution under ambient atmospheric conditions. (b) Resistance ratio (R/R_0) evolution versus the bending time.

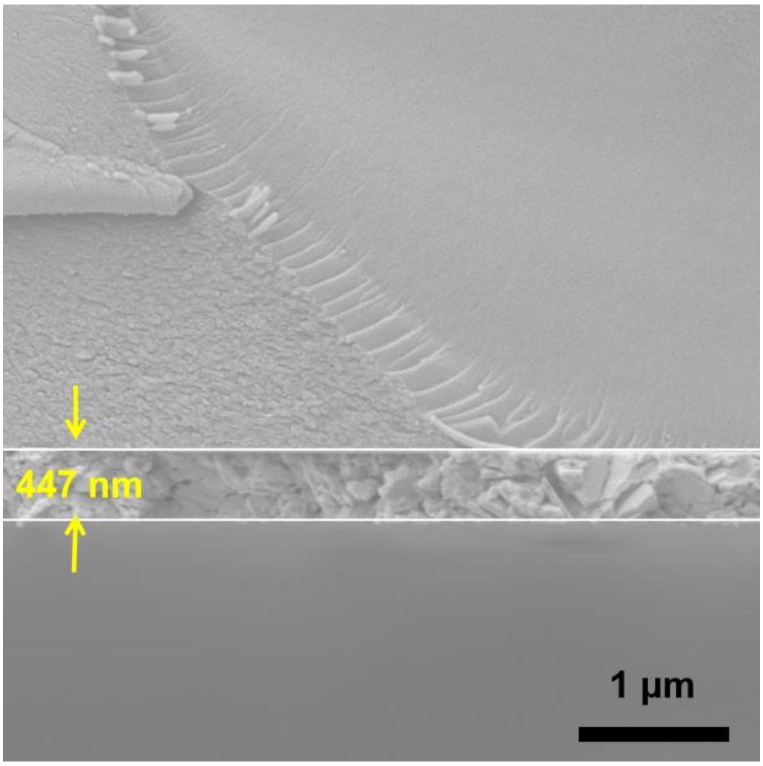

Fig. S40 | BST film. Cross-section SEM image of the BST film.

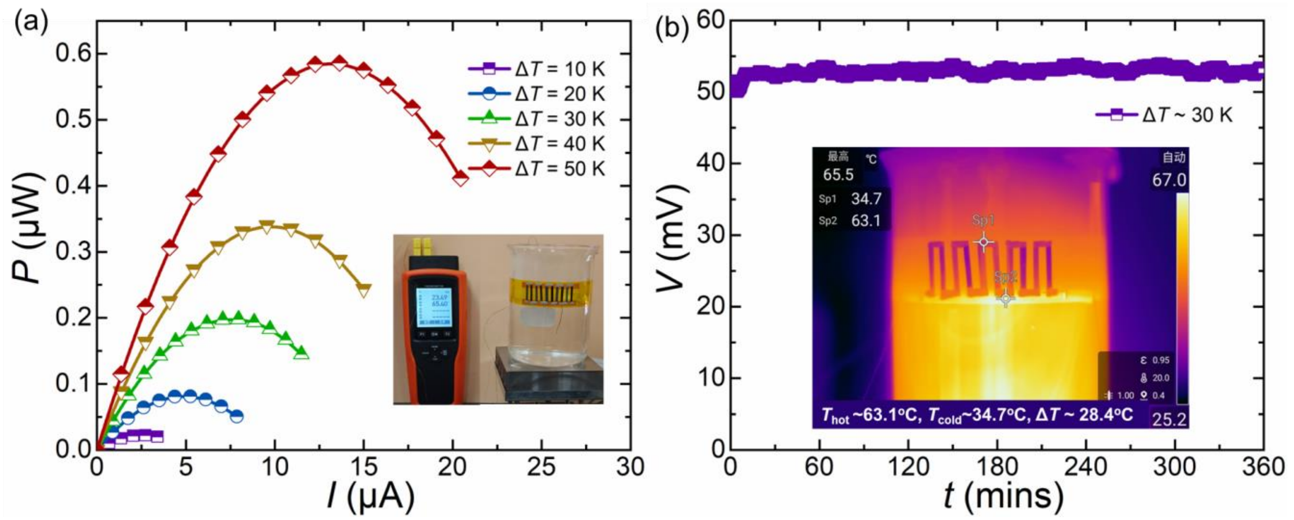

Fig. S41 | Performance of flexible thermoelectric devices. (a) The output power (P) versus temperature different (ΔT = 10K, 20K, 30K, 40K, 50K) of the flexible p-type $Bi_{0.4}Sb_{1.6}Te_3$ (BST) and n-type TiS_2 FTEG with hot water as hotside. The insert was the photo of the prototype flexible thermoelectric device. (b) The time-dependent output voltage of the flexible p-type BST and n-type TiS_2 device with a sustained ΔT of ~30 K. The insert was the infrared image of the prototype flexible thermoelectric device.

Table S4 | Comparison of the Room Thermoelectric Performance of the TMDs based composites.

Materials	Conduction Type	Seebeck Coefficient (μV/K)	Electrical Conductivity (10^4 S/m)	Power Factor(μW/(m·K^2))
1T-TiS_2 nanosheets (This work)	n	-129	4.3	709.4
$Cu_{0.1}TiS_2$ bulk[55]	n	-142.0	5.24	1060.0
TiS_2 single crystals[56]	n	-205	1.2	~540
1T-TiS_2 nanosheets[36]	n	-42.5	2.0	36.1
Al:[TiS2 ns][36]	n	-60.1	6.0	216.7
$TiS_2[(HA)_{0.08}(H_2O)_{0.22}(DMSO)_{0.03}]$[57]	n	-78	7.9	450
$TiS_2(HA)_{0.025}$[58]	n	-155.1	3.56	904
1T MoS_2 nanosheets[59]	p	85.6	0.99	73.1
MoS_2/20 wt.% CNT[60]	p	68±2	2.35±0.05	108.7
SCCM-TaS_2[61]	n	-6.6±0.3	61	340
TaS_2/TiS_2 vdW stack heterostructure (wt.% = 3:1)	n	-38.3	5.96	87.6
TaS_2/TiS_2 vdW stack heterostructure (wt.% = 1:1)	n	-28.6	6.39	52.3
TaS_2/TiS_2 vdW stack heterostructure (wt.% = 1:3)	n	-21.9	7.91	37.6
TaS_2/HA/NMF[62]	n	-11.7	8.76	12.2
1T&2H-WS_2 nanosheets[35]	n	-72	0.1	5-7
1T&2H-$NbSe_2$ nanosheets[35]	p	13	15	26-34

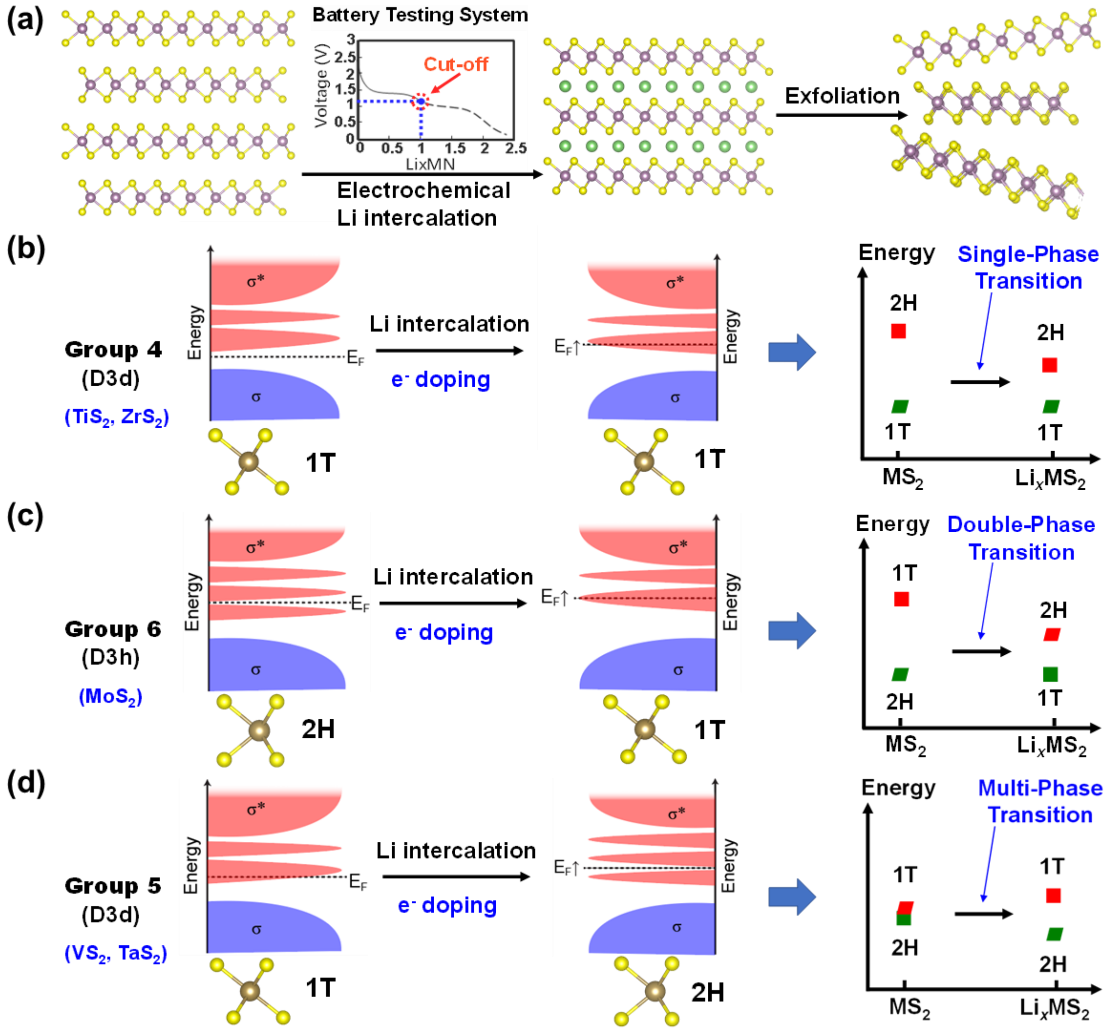

Fig. S42 | Schematic of the phase transition mechanism of TMDs. (a) Electrochemical lithium intercalation-assisted exfoliation of TMDs. (b) Schematic illustration showing filling of d orbitals that are located within the bandgap of bonding (σ) and anti-bonding states (σ*) in group 4 TMDs before and after lithium intercalation (left panel), and the total energy change before and after lithium intercalation (right panel). D3d represents the point group associated with the octahedral coordination of the transition metals. (c) Schematic illustration showing filling of d orbitals that are located within the bandgap of bonding (σ) and anti-bonding states (σ*) in group 6 TMDs before and after lithium intercalation (left panel), and the total energy change before and after lithium intercalation (right panel). D3h represents the point group associated with the trigonal prismatic coordination of the transition metals. (d) Schematic illustration showing filling of d orbitals that are located within the bandgap of bonding (σ) and anti-bonding states (σ*) in group 5 TMDs before and after lithium intercalation (left panel), and the total energy change before and after lithium intercalation (right panel).

Supplementary References

1. Wang H, Si J, Zhang T *et al.* Exfoliated metallic niobium disulfate nanosheets for enhanced electrochemical ammonia synthesis and Zn-N_2 battery. *Appl Catal, B* 2020; **270**: 118892.
2. Shang H, Dun C, Deng Y *et al.* $Bi_{0.5}Sb_{1.5}Te_3$-based films for flexible thermoelectric devices. *J Mater Chem A* 2020; **8**: 4552-61.
3. Kresse G, Furthmuller J. Efficient iterative schemes for ab initio total-energy calculations using a plane-wave basis set. *Phys Rev B* 1996; **54**: 11169-86.
4. Mortensen JJ, Hansen LB, Jacobsen KW. Real-space grid implementation of the projector augmented wave method. *Phys Rev B* 2005; **71**: 035109.
5. Kresse G, Furthmiiller J. Efficiency of ab-initio total energy calculations for metals and semiconductors using a plane-wave basis set. *Comput Mater Sci* 1996; **6**: 15-50.
6. Perdew JP, Burke K, Ernzerhof M. Generalized Gradient Approximation Made Simple. *Phys Rev Lett* 1996; **77**: 3865-8.
7. Grimme S, Antony J, Ehrlich S *et al.* A consistent and accurate ab initio parametrization of density functional dispersion correction (DFT-D) for the 94 elements H-Pu. *J Chem Phys* 2010; **132**: 154104.
8. Monkhorst HJ, Pack JD. Special points for Brillouin-zone integrations. *Phys Rev B* 1976; **13**: 5188-92.
9. Sheppard D, Xiao P, Chemelewski W *et al.* A generalized solid-state nudged elastic band method. *J Chem Phys* 2012; **136**: 074103.
10. Nelson R, Ertural C, George J *et al.* LOBSTER: Local orbital projections, atomic charges, and chemical-bonding analysis from projector-augmented-wave-based density-functional theory. *J Comput Chem* 2020; **41**: 1931-40.
11. Deringer VL, Tchougreeff AL, Dronskowski R. Crystal orbital Hamilton population (COHP) analysis as projected from plane-wave basis sets. *J Phys Chem A* 2011; **115**: 5461-66.
12. Steinberg S, Dronskowski R. The Crystal Orbital Hamilton Population (COHP) Method as a Tool to Visualize and Analyze Chemical Bonding in Intermetallic Compounds. *Crystals* 2018; **8**: 225.
13. Sam RT, Umakoshi T, Verma P. Probing stacking configurations in a few layered MoS_2 by low frequency Raman spectroscopy. *Sci Rep* 2020; **10**: 21227.
14. Manzeli S, Ovchinnikov D, Pasquier D *et al.* 2D transition metal dichalcogenides. *Nat Rev Mater* 2017; **2**: 17033.
15. Chhowalla M, Shin HS, Eda G *et al.* The chemistry of two-dimensional layered transition metal dichalcogenide nanosheets. *Nat Chem* 2013; **5**: 263-75.
16. Ding W, Hu L, Dai J *et al.* Highly Ambient-Stable 1T-MoS_2 and 1T-WS_2 by Hydrothermal Synthesis under High Magnetic Fields. *ACS Nano* 2019; **13**: 1694-702.
17. Voiry D, Mohite A, Chhowalla M. Phase engineering of transition metal dichalcogenides. *Chem Soc Rev* 2015; **44**: 2702-12.
18. Lin C, Zhu X, Feng J *et al.* Hydrogen-incorporated TiS_2 ultrathin nanosheets with ultrahigh conductivity for stamp-transferrable electrodes. *J Am Chem Soc* 2013; **135**: 5144-51.
19. Wróblewska ADA, Judek J, Wilczyński K *et al.* Temperature-induced phonon behavior in titanium disulfide TiS_2 nanosheets. *J Raman Spectrosc* 2019; **50**: 1114-9.
20. Zhu J, Wang Z, Yu H *et al.* Argon Plasma Induced Phase Transition in Monolayer MoS_2. *J Am Chem Soc* 2017; **139**: 10216-9.
21. Tan SJR, Abdelwahab I, Ding Z *et al.* Chemical Stabilization of 1T' Phase Transition Metal Dichalcogenides with Giant Optical Kerr Nonlinearity. *J Am Chem Soc* 2017; **139**: 2504-11.
22. Li QQ, Guo YL, Tian Y *et al.* Activating VS_2 basal planes for enhanced NRR electrocatalysis: the

synergistic role of S-vacancies and B dopants. *J Mater Chem A* 2020; **8**: 16195-202.
23. Wang J, Luo N, Wu J *et al.* Hierarchical spheres constructed by ultrathin VS_2 nanosheets for sodium-ion batteries. *J Mater Chem A* 2019; **7**: 3691-6.
24. Zhang L, Zhu J, Mai L *et al.* Understanding the electrochemical reaction mechanism of VS_2 nanosheets in lithium-ion cells by multiple in situ and ex situ x-ray spectroscopy. *J Phys D: Appl Phys* 2018; **51**: 494001.
25. Zhao X, Wan D, Song P *et al.* Engineering covalently bonded 2D layered materials by self-intercalation. *Nature* 2020; **581**: 171-7.
26. Peng J, Wu J, Li X *et al.* Very Large-Sized Transition Metal Dichalcogenides Monolayers from Fast Exfoliation by Manual Shaking. *J Am Chem Soc* 2017; **139**: 9019-25.
27. Wen W, Zhu Y, Dang C *et al.* Raman Spectroscopic and Dynamic Electrical Investigation of Multi-State Charge-Wave-Density Phase Transitions in 1 T-TaS_2. *Nano Lett* 2019; **19**: 1805-13.
28. Fan S, Neal S, Won C *et al.* Excitations of Intercalated Metal Monolayers in Transition Metal dichalcogenides. *Nano Lett* 2021; **21**: 99-106.
29. Zhao M, Yang M, Chen D *et al.* Synergism on Electronic Structures and Active Edges of Metallic Vanadium Disulfide Nanosheets via Co Doping for Efficient Hydrogen Evolution Reaction in Seawater. *ChemCatChem* 2021; **13**: 2138-44.
30. Hossain M, Wu J, Wen W *et al.* Chemical Vapor Deposition of 2D Vanadium Disulfide and Diselenide and Raman Characterization of the Phase Transitions. *Adv Mater Interfaces* 2018; **5**: 1800528.
31. Yamada A, Koizumi H, Nishimura S-I *et al.* Room-temperature miscibility gap in Li_xFePO_4. *Nat Mater* 2006; **5**: 357-360.
32. Padhi AK, Nanjundaswamy KS, Goodenough JB. Phospho-olivines as Positive-Electrode Materials for Rechargeable Lithium Batteries. *J Electrochem Soc* 1997; **144**: 1188.
33. Sakhuja N, Jha RK, Chaurasiya R *et al.* 1T-Phase Titanium Disulfide Nanosheets for Sensing H_2S and O_2. *ACS Appl Nano Mater* 2020; **3**: 3382-94.
34. Hu Z, Tai Z, Liu Q *et al.* Ultrathin 2D TiS_2 Nanosheets for High Capacity and Long-Life Sodium Ion Batteries. *Adv Energy Mater* 2019; **9**: 1803210.
35. Oh JY, Lee JH, Han SW *et al.* Chemically exfoliated transition metal dichalcogenide nanosheet-based wearable thermoelectric generators. *Energy Environ Sci* 2016; **9**: 1696-705.
36. Zhou Y, Wan J, Li Q *et al.* Chemical Welding on Semimetallic TiS_2 Nanosheets for High-Performance Flexible n-Type Thermoelectric Films. *ACS Appl Mater Interfaces* 2017; **9**: 42430-7.
37. Tian Y, Cheng Y, Huang J *et al.* Epitaxial growth of large area ZrS_2 2D semiconductor films on sapphire for optoelectronics. *Nano Res* 2022; **15**: 6628-35.
38. Mattinen M, Popov G, Vehkamäki M *et al.* Atomic Layer Deposition of Emerging 2D Semiconductors, HfS_2 and ZrS_2, for Optoelectronics. *Chem Mater* 2019; **31**: 5713-24.
39. Lin Z, Liu Y, Halim U *et al.* Solution-processable 2D semiconductors for high-performance large-area electronics. *Nature* 2018; **562**: 254-8.
40. Wu J, Liu M, Chatterjee K *et al.* Exfoliated 2D Transition Metal Disulfides for Enhanced Electrocatalysis of Oxygen Evolution Reaction in Acidic Medium. *Adv Mater Interfaces* 2016; **3**: 1500669.
41. Hau HH, Duong TTH, Man NK *et al.* Enhanced NO_2 gas-sensing performance at room temperature using exfoliated MoS_2 nanosheets. *Sens Actuators, A* 2021; **332**: 113137.
42. Parra-Alfambra AM, Casero E, Vázquez L *et al.* MoS_2 nanosheets for improving analytical performance of lactate biosensors. *Sens Actuators, B* 2018; **274**: 310-7.
43. Bang GS, Nam KW, Kim JY *et al.* Effective liquid-phase exfoliation and sodium ion battery application of MoS_2 nanosheets. *ACS Appl Mater Interfaces* 2014; **6**: 7084-9.
44. Mak KF, Lee C, Shan JH *et al.* Atomically thin MoS_2: a new direct-gap semiconductor. *Phys Rev Lett*

2010; **105**: 136805.
45. Zhou J, Shen Y, Lv F *et al.* Ultrathin Metallic NbS_2 Nanosheets with Unusual Intercalation Mechanism for Ultra-Stable Potassium-Ion Storage. *Adv Funct Mater* 2022; **32**: 2204495.
46. Kim Y, Kwon KC, Kang S *et al.* Two-Dimensional NbS_2 Gas Sensors for Selective and Reversible NO_2 Detection at Room Temperature. *ACS Sens* 2019; **4**: 2395-402.
47. Wang Z, Cheon C-Y, Tripathi M *et al.* Superconducting 2D NbS_2 Grown Epitaxially by Chemical Vapor Deposition. *ACS Nano* 2021; **15**: 18403-10.
48. Feng J, Sun X, Wu CZ *et al.* Metallic few-layered VS_2 ultrathin nanosheets: high two-dimensional conductivity for in-plane supercapacitors. *J Am Chem Soc* 2011; **133**: 17832-8.
49. Kumar GM, Ilanchezhiyan P, Cho HD *et al.* Ultrathin VS_2 nanodiscs for highly stable electro catalytic hydrogen evolution reaction. *Int J Energy Res* 2019; **44**: 811-20.
50. Salavati M, Rabczuk T. Application of highly stretchable and conductive two-dimensional 1T VS_2 and VSe_2 as anode materials for Li-, Na- and Ca-ion storage. *Comput Mater Sci* 2019; **160**: 360-7.
51. Yu Q, Luo Y, Qiu S *et al.* Tuning the Hydrogen Evolution Performance of Metallic 2D Tantalum Disulfide by Interfacial Engineering. *ACS Nano* 2019; **13**: 11874-81.
52. Li H, Tan Y, Liu P *et al.* Atomic-Sized Pores Enhanced Electrocatalysis of TaS_2 Nanosheets for Hydrogen Evolution. *Adv Mater* 2016; **28**: 8945.
53. Kovalska E, Roy PK, Antonatos N *et al.* Photocatalytic activity of twist-angle stacked 2D TaS_2. *npj 2D Mater Appl* 2021; **5**: 68.
54. Wu J, Peng J, Yu Z *et al.* Acid-Assisted Exfoliation toward Metallic Sub-nanopore TaS_2 Monolayer with High Volumetric Capacitance. *J Am Chem Soc* 2018; **140**: 493-8.
55. Guilmeau E, Bréard Y, Maignan A. Transport and thermoelectric properties in Copper intercalated TiS_2 chalcogenide. *Appl Phys Lett* 2011; **99**: 052107.
56. Salah N, Abdullahi S, Baghdadi N *et al.* High Thermoelectric Power Generation below Room Temperature by TiS_2 Compact Pellet. *ACS Appl Electron Mater* 2023; **6**: 2839-50.
57. Wan C, Gu X, Dang F *et al.* Flexible n-type thermoelectric materials by organic intercalation of layered transition metal dichalcogenide TiS_2. *Nat Mater* 2015; **14**: 622-7.
58. Wan C, Tian R, Kondou M *et al.* Ultrahigh thermoelectric power factor in flexible hybrid inorganic-organic superlattice. *Nat Commun* 2017; **8**: 1024.
59. Huang H, Cui Y, Li Q *et al.* Metallic 1T phase MoS_2 nanosheets for high-performance thermoelectric energy harvesting. *Nano Energy* 2016; **26**: 172-9.
60. Li JH, Shi QW, Röhr JA *et al.* Flexible 3D Porous MoS_2/CNTs Architectures with ZT of 0.17 at Room Temperature for Wearable Thermoelectric Applications. *Adv Funct Mater* 2020; **30**: 2002508.
61. Wang S, Yang X, Hou L *et al.* Organic covalent modification to improve thermoelectric properties of TaS_2. *Nat Commun* 2022; **13**: 4401.
62. Li WH, Zhang XF, Liu H *et al.* Two-dimensional van der Waals stack heterostructures for flexible thermoelectrics. *Nano Energy* 2024; **125**: 109605.
